# Bio-friendly long-term subcellular dynamic recording by self-supervised image enhancement microscopy

**DOI:** 10.1038/s41592-023-02058-9

**Published:** 2023-11-13

**Authors:** Guoxun Zhang, Xiaopeng Li, Yuanlong Zhang, Xiaofei Han, Xinyang Li, Jinqiang Yu, Boqi Liu, Jiamin Wu, Li Yu, Qionghai Dai

**Affiliations:** 1 Department of Automation, Tsinghua University, Beijing, China; 2 Institute for Brain and Cognitive Sciences, Tsinghua University, Beijing, China; 3 State Key Laboratory of Membrane Biology, Tsinghua University–Peking University Joint Center for Life Sciences, Beijing Frontier Research Center for Biological Structure, School of Life Sciences, Tsinghua University, Beijing, China; 4 Tsinghua Shenzhen International Graduate School, Tsinghua University, Shenzhen, China; 5 Shanghai AI Laboratory, Shanghai, China

**Keywords:** Cellular imaging, Fluorescence imaging, Image processing

## Abstract

Fluorescence microscopy has become an indispensable tool for revealing the dynamic regulation of cells and organelles. However, stochastic noise inherently restricts optical interrogation quality and exacerbates observation fidelity when balancing the joint demands of high frame rate, long-term recording and low phototoxicity. Here we propose DeepSeMi, a self-supervised-learning-based denoising framework capable of increasing signal-to-noise ratio by over 12 dB across various conditions. With the introduction of newly designed eccentric blind-spot convolution filters, DeepSeMi effectively denoises images with no loss of spatiotemporal resolution. In combination with confocal microscopy, DeepSeMi allows for recording organelle interactions in four colors at high frame rates across tens of thousands of frames, monitoring migrasomes and retractosomes over a half day, and imaging ultra-phototoxicity-sensitive *Dictyostelium* cells over thousands of frames. Through comprehensive validations across various samples and instruments, we prove DeepSeMi to be a versatile and biocompatible tool for breaking the shot-noise limit.

## Abstract (web summary)

DeepSeMi is a self-supervised denoising framework that can enhance SNR over 12 dB across diverse samples and imaging modalities. DeepSeMi enables extended longitudinal imaging of subcellular dynamics with high spatiotemporal resolution.

## Main

The magnificence of harmonically orchestrated systems, organs, tissues and cells attracts people to explore the mystery of life^1,2^. In the complex milieu of the cell, organelles collaborate and interact with the cytoskeleton, orchestrating an array of physiological functions that underpin the vitality of organisms. Such gorgeous patterns reflect how organelles interplay in highly dynamic yet organized interactions capable of orchestrating complex cellular functions^3^. Visualizing the functionality and complexity of organelles in their native states requires high spatiotemporal resolution observation without perturbing this physiologically presented regulations in the long term.

Standing in the center of approaches dedicated to probing and deciphering the micro world is the noninvasive fluorescent microscope, which is capable of high spatiotemporal resolution^4^ and good protein specificity^5^. Combined with fluorescent proteins^6,7^ and indicators^8^, remarkable advances in enriched fluorescence microscopy^1,9–12^ have enabled discoveries across many disciplines, including cell biology^13^, immunology^14^ and neuroscience^15^. However, a fundamental challenge associated with fluorescence microscopy is the limited photon budget, leading to insufficient signal-to-noise ratio (SNR)^16^. The low quantum yield of fluorescent indicators and the stochastic nature of noise make the contamination inevitable^6^, aggravating measurement uncertainty and impairing downstream quantitative analysis, including cell segmentation^17^, cell tracking^18^ and signal extraction^19^. Overcoming this limitation physically requires increasing excitation dosage^20^ or increasing the expression of indicators^21^, but these options can cause artifacts in living systems, altering the morphological and functional interpretations that follow. Such a condition is even worse in long-term imaging that necessitates repeated illumination over the same sample hundreds and thousands of times to observe pivotal processes like cell proliferation^22^, migration^13,23^, organelle interactions^24,25^ and neuronal firing^26^. To mitigate noise contamination without excessive light-exposure-induced photobleaching and phototoxicity, which perturbs the sample in its native state, microscopists have to sacrifice imaging speed, resolution or dimension^27^.

Despite limited advances achieved across physical approaches, numerous algorithmic approaches have been proposed to break the shot-noise limit by using statistics of the noise^28^. Traditional denoising methods that exploit canonical properties of the noise (such as Gaussianity^29^ and structures in the signal^30^) achieve great success in photographic denoising^30^ but have limited performance in complex, turbulent and dynamic living systems and come at marked computational cost. In contrast, supervised learning methods utilizing a data-driven prior learned from paired noisy and clean measurements have proven valid as long as samples are drawn from the same distribution^31,32^. To extend the generalization, the requirement of clean data can be further replaced by additional independent noisy measurements^33^, fertilizing breakthroughs in interpolating noise-contaminated functional data^34,35^. However, for many reasons, neither of these supervised methods circumvents the denoising of videographic high-resolution recording, with both intensity fluctuations and deformations of living organisms or organelles. First, since the same physiological phenomenon would not repeat twice for each cell or organism, the requirement of clean or ‘groundtruth’ data by supervised methods can be satisfied only through simulations, which have marked gaps between training and inferring domains^36^. Second, even only the paired noisy data is required in interpolation-based methods like DeepInterpolation^35^ and DeepCAD^34^, the precondition of interframe continuity probably limits visualizing rapid transformations of living organisms or organelles. Third, the imperfect blind-spot techniques employed in these self-supervised methods curtail denoising performance, thereby necessitating a compromise between preserving accurate visualization and maintaining the safety of the organism or, alternatively, risking the health of the sample through excessive captures to ensure quality visualization.

Here, we overcome the aforementioned limitations and propose a deep self-supervised learning enhanced microscope (DeepSeMi)—an open-source tool that readily and veritably increases the SNR over 12 dB across various conditions and systems, and catalyzes noise-free videography of diverse structures and functional signals with minimized photodamage in the long term. DeepSeMi explores noise priors that root in data itself through concatenating newly designed eccentric convolution filters and eccentric blind convolution filters with intentionally limited receptive fields across both spatial and temporal dimensions (Supplementary Fig. 1; Methods). DeepSeMi outperforms other methods in both performance and generalization, and computationally amplifies the photon budget of several instruments in long-term tracking of organellar and organismal activities without the burden of the higher light doses used in traditional approaches. Through DeepSeMi, organelle interactions in their native states inside four-color-labeled L929 cells were recorded over 30 min and 14,000 timepoints in high SNR on a confocal microscope—a widely used instrument that offers high resolution, often with the cost of photodamage. Aided by DeepSeMi, sensitive structures such as migrasomes and retractosomes were frequently tracked in a half-day-long session, uninterrupted and without measurable photobleaching, and several organelles in these images could be segmented free of false positives due to noise contamination. Even fragile and photosensitive samples like *Dictyostelium* cells were also clearly recorded over 36,000 shots in multicolor, attributed to DeepSeMi enhancement. Not limited to cultured cells and organisms, the capability and generality of DeepSeMi are also demonstrated in a series of photon-limited imaging experiments over various species, including nematodes, zebrafish and mice, all intravitally.

## Results

### DeepSeMi accomplishes single-flow high-fidelity denoising

**Fig. 1:**
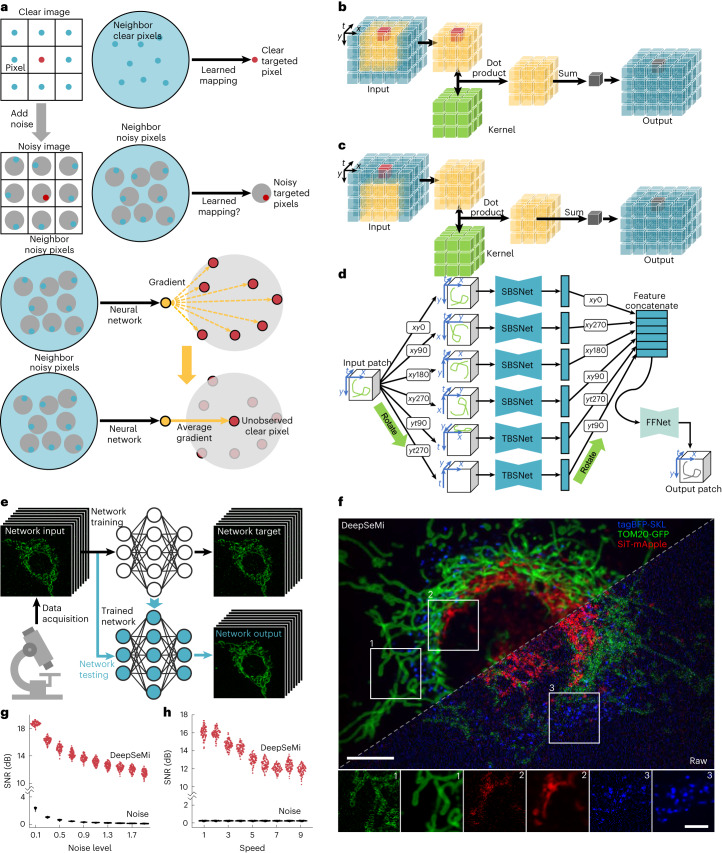
DeepSeMi accomplishes self-supervised video denoising based on the statistical characteristics of noise. **a**, Statistical principle of DeepSeMi. In pristine conditions, a well-defined mapping from neighboring pixels to a central pixel can be established, owing to local structural continuity (first row). However, when neighboring pixels are corrupted by noise (second row), and a neural network is tasked to establish the learned mapping (third row), it ultimately results in averaged gradients on the target pixel. In this context, the assumption of noise contaminations having a zero mean ensures that the averaged gradients can retrieve clean information from the target pixel, which was initially unobserved (fourth row). This rationale forms the basis of the operation of DeepSeMi. **b**, Schematics of the 3D eccentric convolution. In a 3D (*x*, *y*, *t*) patch (blue), an eccentric neighborhood (yellow) surrounding the target pixel (red) is multiplied with a learnable kernel (green), and the dimension-reduced summation forms an output pixel (gray) in the output patch; in eccentric convolution, the eccentric neighborhood still contains the target pixel. **c**, Schematics of the 3D eccentric blind-spot convolution. Symbols as in **b**; in eccentric blind-spot convolution, the eccentric neighborhood does not contain the target pixel, and thereby the output pixel (gray) excludes the information of the target pixel (red). **d**, Structure of the proposed spatiotemporal hybrid 3D blind-spot convolutional neural network. The neural network consists of six subnetworks with the same structure and a final feature FFnet. Among six subnetworks, four spatial 3D blind-spot convolutional neural networks (SBSnet, top four) and two temporal 3D blind-spot convolutional neural networks (TBSnet, bottom two) share the same network architecture. The input patch is rotated and fed into each subnetwork, and the output features are rotated accordingly to match each other’s size before feature fusion (Methods). **e**, DeepSeMi enables SNR enhancement with only experimental data through a single shot. Low-SNR recordings can be used to train the proposed self-supervised neural network in situ, which enables the trained network to enhance low SNR recordings itself. **f**, Raw (right) and DeepSeMi denoised (left) images of mitochondria (green), peroxisomes (blue) and Golgi (red) in a L929 cell at 1,800 frames per channel during 180 s. The lower images show enlarged views of the regions enclosed in white boxes in the upper image. Scale bar, 10 µm (upper) and 4 µm (lower); *n* = 9 cells examined over three independent experiments. **g**,**h**, DeepSeMi denoising performance indicated by the SNR over different noise levels (Supplementary Figs. 7 and 8) and content speeds (Supplementary Fig. 9).

The innovation of DeepSeMi is rooted in a full exploitation of noise statistics. Studies show that mutual mappings from neighbors to a centered pixel can be well established, even excluding the pixel itself, due to local structure continuity^37^. Under noisy conditions, although those mappings are significantly degraded, the average of the degraded mappings relocate the clear pixel information, facilitating estimation of each clear pixel from the surrounding noisy spatiotemporal neighborhood^38^ (Fig. 1a). Based on that observation, DeepSeMi thereby establishes mappings between each pixel of the noisy videography and its surrounding pixels to effectively denoise videography. The utility of pixel-level noise statistics makes DeepSeMi robust, even over a single noisy shot, and consequently eliminates the need for excessive captures to ensure performance compared with previous techniques^34,35^ (Fig. 1e).

To establish these special mappings, two new convolution kernels were developed to convey the aforementioned thought with optimized efficiency in DeepSeMi. The first convolutional kernels receive both the inferred pixel and its eccentrically surrounded neighbors to keep the DeepSeMi efficient in both restoring structures and eliminating noise (Fig. 1b and Supplementary Fig. 1b), and are named accordingly as eccentric convolution. The second convolution kernels resemble the blind-spot property by receiving only the eccentrically surrounded neighbors of the inferred pixel to achieve an even stronger noise cleanse ability (Fig. 1c and Supplementary Fig. 1c), and are named accordingly as eccentric blind-spot convolution. A single flow across the blind-spot convolution thereby facilitates each input noisy pixel to be synthesized only by the neighbors, without itself, accomplishing denoising in a self-supervised learning manner very efficiently (Supplementary Figs. 2–4). The rationale for combining both filters in DeepSeMi is to achieve an appropriate balance between the preservation of detail and noise robustness with the assistance of the pixel-level blind-spot technique (Methods). Six branches composed of these two convolutional filters deliver permutational receptive-limited fields of both spatial and temporal dimensions, and are further merged by a feature fusion network (FFnet) to form representations of the output video block (Fig. 1d). Computation losses are therefore differentiated between the input and output to guide the updates of the network parameters through backpropagation (Supplementary Fig. 5). Through ablation studies (Supplementary Fig. 6), we indeed confirmed that the multibranched structure of DeepSeMi is vital for achieving high-performance denoising. The comprehensively optimized DeepSeMi also leverages a time-to-feature folding operation, which feeds more temporal information without increasing additional computational cost to increase performance (Methods).

We benchmarked the denoising capability of DeepSeMi through extensive simulations compared with various mainstream methods^34,35,39–43^. To fully emulate real experiments in complex situations, we evaluated those methods in Moving MNIST (Modified National Institute of Standards and Technology) datasets where both the noise level and the movement speed of the contents are varied over a large range of values. Among all methods tested, DeepSeMi achieved the best denoising results across all noise levels, achieving 15 dB higher SNR compared with raw capture under extremely noisy conditions (Fig. 1g and Supplementary Figs. 7 and 8). While most of the literature compares SNR merely in static scenes, we further evaluated the denoising ability of those methods, encountering swift content across various speeds. With increasing moving speed of content, DeepSeMi stayed in the top tier in terms of restoration quality over other methods, with SNR improvement of at least 12 dB (Fig. 1h and Supplementary Fig. 9), where techniques using frame-level noise statistics (DeepCAD^34^ and DeepInterpolation^35^) lowered their performance quickly due to the frame interpolation nature (Supplementary Figs. 10 and 11). In more complicated Poisson noise contamination, where noise scale correlates with image intensity (Supplementary Fig. 12), DeepSeMi still outperformed all other methods. Furthermore, DeepSeMi has been demonstrated to effectively handle mixed Poisson and Gaussian noise (Supplementary Fig. 13) and to preserve spatial resolution (Supplementary Fig. 14).

Although DeepSeMi was trained at a moderate content speed (Supplementary Fig. 15a–c), performance remained high as content speed varied. We further tested the generalization of DeepSeMi in experiments where DeepSeMi was trained for the modality of mitochondrial membrane but tested with colabeled cell membrane and mitochondrial matrix data (Supplementary Figs. 16a and 17). We found the noise-contaminated mitochondrial matrix were cleaned by DeepSeMi, in both clustered forms close to the cell center and scattered forms at the cell edge (Supplementary Figs. 16b–e and 17). Composited interactions of both membranes and mitochondrial matrix were clearly shown after DeepSeMi enhancement that was trained only on third and unimodal data (Supplementary Fig. 16f–h and 17). Colabeled mitochondrial images were used to examine the self-consistency of DeepSeMi (Supplementary Fig. 18a). Here, we observed that the denoised results were highly aligned between the two channels, where each was labeled by a distinct fluorescent indicator (Supplementary Fig. 18c). The demonstrated generalization and self-consistency of DeepSeMi ensure the fidelity of observation across complicated microenvironments during long-term cellular imaging, accomplishing apparent enhancement in recovering both structural and functional diversity (Fig. 1f, Supplementary Fig. 19 and Supplementary Video 1).

### Experimentally corroborating DeepSeMi denoising performance

**Fig. 2:**
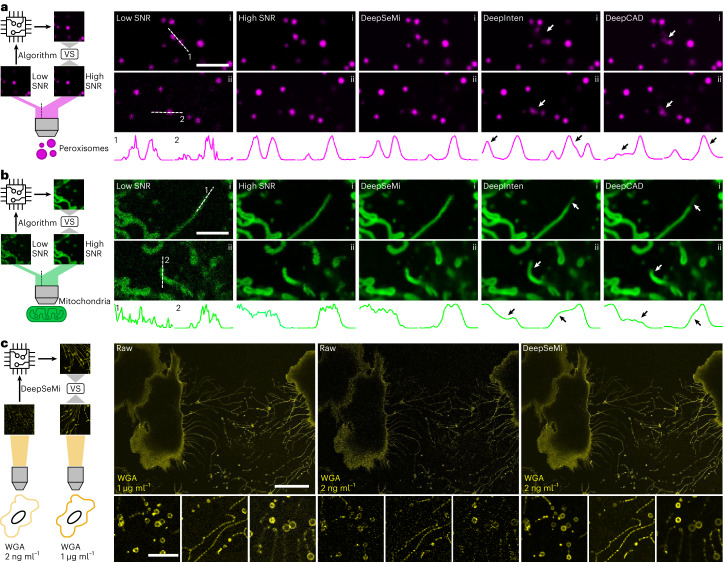
Experimental verification of DeepSeMi across various samples. **a**, Benchmarking denoising performance on mOrange2-SKL labeled peroxisomes. Left: peroxisomes were imaged through our simultaneous high- and low-SNR confocal imaging system (Supplementary Fig. 20; Methods). VS, versus. Right: raw captures in low SNR as algorithm input, raw captures in high SNR as reference and recovered captures by algorithms including DeepSeMi, DeepIntern and DeepCAD. Two representative frames (i and ii) are shown for each method. Intensity profiles along the white dashed lines are plotted underneath. White arrows in each capture label apparent artifacts after algorithm recovery with high-SNR captures as reference. Black arrows in intensity panels mark apparent artifacts along the white dashed lines of each method with high-SNR captures as reference. Scale bar, 3 μm. *n* = 30 cells examined over three independent experiments. **b**, Benchmarking denoising performance on Tom20-GFP labeled mitochondria; layouts, symbols and markers as in **a**. Scale bar, 3 μm. *n* = 62 cells examined over three independent experiments. **c**, Quantification of DeepSeMi enhancement through enriched fluorescent dye concentration. Left: schematic diagram of the quantification experiment, where captures of cell labeled by 1 μg ml^–1^ WGA647 were approximated as the groundtruth (right side). WGA, wheat germ agglutinin. Right, raw captures of cells with dyes diluted 500 times (2 ng ml^–1^, middle image) were sent for DeepSeMi enhancement (rightmost image). Upper, global views (scale bar, 30 μm); lower: enlarged views. Scale bar, 10 μm. *n* = 27 cells examined over three independent experiments.

To perform a direct and quantitative validation of performance and accuracy of DeepSeMi, we modified a commercial confocal system for acquiring simultaneous high- and low-SNR cell images (Supplementary Fig. 20; Methods). By bias splitting the emission spectrum into two portions for two photomultiplier tubes (PMTs), we acquired paired images with 18.4-fold SNR differences for green fluorescent protein (GFP), 20.3-fold for mOrange2 and 15.5-fold for Fluor 657 (Supplementary Fig. 21). We found that DeepSeMi appropriately removed shot-noise in imaging peroxisomes, mitochondria and membranes, and recovered accurate organelle structures compared with the high-SNR groundtruth (Fig. 2a and Supplementary Fig. 22). We noted the imaging SNR was improved more than 15-fold, considering that the noise of DeepSeMi-enhanced recordings were even more negligible than the corresponding high-SNR reference. We next benchmarked our DeepSeMi with other denoising technologies through the built simultaneous high- and low-SNR imaging system (Supplementary Figs. 23–25). We found frame-interpolation-based methods (DeepIntern^35^ and DeepCAD^34^) generated apparent artifacts that were highly similar to the natural morphologies of peroxisomes (Fig. 2a and Supplementary Fig. 23a) or mitochondria (Fig. 2b and Supplementary Fig. 23b), which might strongly alter potential biological conclusions. On the other hand, utilizing the pixel-level blind-spot technique, DeepSeMi consistently retrieved accurate organelle structures without exhibiting discernible artifacts regardless of apparent complicated morphology deformations (Supplementary Fig. 23a–c). In summary, DeepSeMi became top tier among the tested denoising techniques in terms of noise suppression (Supplementary Figs. 7, 8 and 12), artifact rejection (Supplementary Figs. 23–25) and complicated motion compatibility (Supplementary Figs. 9–11), as evidenced via various simulations (Supplementary Figs. 7–12) and experiments (Supplementary Figs. 23–25). Our paired high- and low-SNR datasets, extensively covering various organelles, SNRs and structural complexity, have been made available as open-source tools to the research community (Data availability). Moreover, we have substantiated that DeepSeMi maintains the linearity of the intensities of the examined structures throughout the denoising process (Supplementary Fig. 26), while preserving high fidelity across a wide range of imaging speeds (Supplementary Fig. 27).

After corroborating the accuracy of DeepSeMi in removing noise contaminations, we next proved that DeepSeMi computationally amplifies the photon budget in long-term imaging of organelles and organisms without the burden of exacerbating sample health seen in traditional approaches. Photon budget is the eventual bottleneck of observing swift intracellular organelle interactions, cell migration and multicellular interactions over the long term^44,45^ which results in insufficient and scant data in most conditions due to the compromising effects of photobleaching and phototoxicity. To illustrate this, we conducted extensive evaluations to investigate imaging conditions in which light-sensitive mitochondria can be recorded in their native state (Supplementary Fig. 28). We found that healthy mitochondria can withstand only 45.3 μW laser power (2%, 488 nm) (Supplementary Fig. 29) for a 3-min session at 30 frames per second (fps) in a commercial confocal microscope without apparent photobleaching (Supplementary Fig. 28; Methods). Higher laser dosage quickly bleached the fluorescence, defiling the imaging process due to missing mitochondrial structural information. Although using such a low power dosage seems to be expedient for long-term cellular observation, it exacerbated the noise contamination associated with the observations and yielded barely characterized structures (Supplementary Fig. 28d); observation was even more difficult when the mitochondria were densely clustered. On the other hand, using DeepSeMi, under even 14.6 μW (0.5%, 488 nm) power dosage, mitochondria can be denoised faithfully, with intact and natural form restored (Supplementary Figs. 30 and 31). Under that excitation power, the fluorescent intensity drop was undetectable, suggesting that DeepSeMi enhancement not only accomplished high-fidelity recording but even eliminated potential photobleaching (Supplementary Video 2). From other perspectives, the computational enhancement of DeepSeMi increases the available photon budget of optical instruments. Considering that DeepSeMi achieves even higher visualization quality of mitochondrial structures at 23.1 μW (1%, 488 nm) (Supplementary Fig. 28b) than raw captures at 537 μW (32%, 488 nm) (Supplementary Fig. 28h), the available photon budget was enlarged at least tenfold.

We verified the photon budget enlargement of DeepSeMi quantitatively across three dimensions. In the first dimension, we approximated the photon budget enlargement as the multiplication of excitation power in raw captures through which the same or similar SNR of DeepSeMi enhancement can be achieved (Supplementary Fig. 32). We found that at least 15-fold greater power dosage in raw frames was required to produce the same level of imaging quality as DeepSeMi enhancement across various noisy conditions, verifying that DeepSeMi enlarges the photon budget by at least 15-fold (Supplementary Fig. 32). In the second dimension, we investigated the photon budget enlargement as the excessive concentration of dyes in raw captures to approach the DeepSeMi-enhanced SNR. We proved that DeepSeMi achieved no-compromise results across migrasomes, lysosomes and mitochondria using dye concentrations diluted over 50 times, and the resulting captures were comparable with nondiluted captures (Fig. 2c and Supplementary Fig. 33). In the third dimension, we investigated the excessive recording duration brought by DeepSeMi over imaging FM4-64-labeled cells. We found a 476-min recording length could be achieved through DeepSeMi enhancement over an imaging power of 0.5% with comparable quality and SNR compared with those achieved with 10% imaging power (Supplementary Fig. 34). On the other hand, necrosis due to phototoxicity appeared ∼42 min after the start of imaging session in 10% imaging power, precluding investigation of any long-term cellular activities, such as migration, division and autophagy. Through the above validations, we demonstrated that the photon budget as multiplied by DeepSeMi greatly extends the capacity of the optical microscope to pursue higher spectral complexity, higher frame rate and longer recording sessions, eliminating the risks of higher power dosage and dye concentration inducing greater cytotoxicity and perturbation than native regulation.

### DeepSeMi unlocks high-speed long-term imaging with minimized photobleaching

**Fig. 3:**
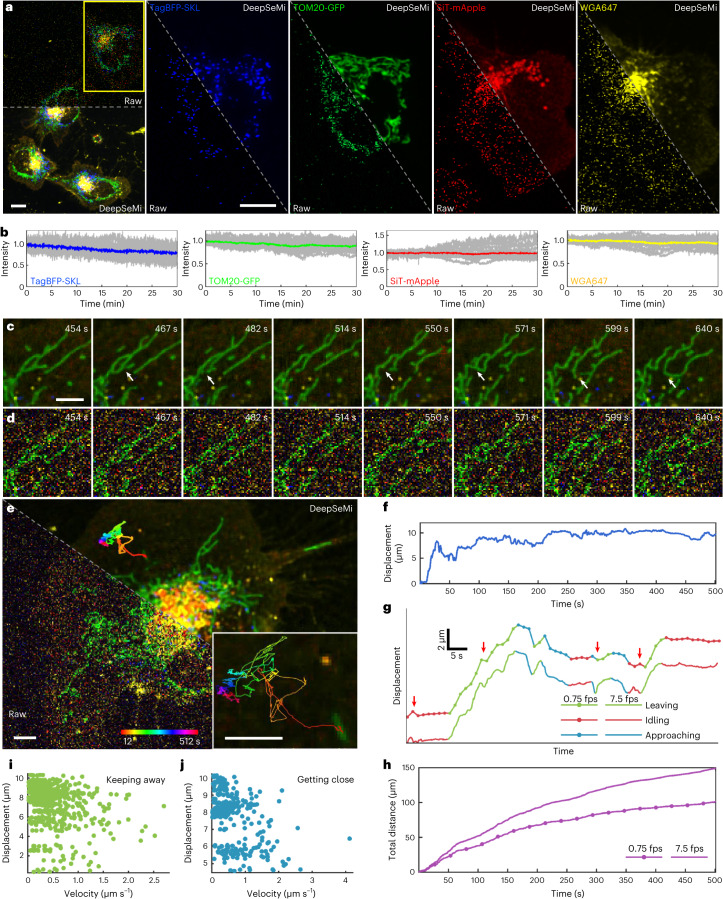
Long-term, high-temporal resolution and low phototoxicity imaging of organelle interactions by DeepSeMi. **a**, Left: raw (top) and DeepSeMi-enhanced (bottom) micrographs of an L929 cell expressing fluorescent proteins (TOM20-GFP, TagBFP-SKL and SiT-mApple) and labeled by WGA647. Right: individual channels of the yellow box marked in the left panel are displayed separately. Scale bar, 10 μm for both global and enlarged views. *n* = 14 cells examined over four independent experiments. **b**, Fluorescence intensity fluctuations (*n* = 10) of four channels during a 30-min imaging session (13,500 frames) at 2% light intensity. Fluorescence intensity curves were normalized to initial values. **c**,**d**, DeepSeMi-enhanced (**c**) and raw (**d**) timelapse images that reflect mitochondrial morphological changes during low-light recording. White arrows mark the process of mitochondrial fission and fusion. Scale bar, 5 µm. *n* = 14 cells examined over four independent experiments. **e**, Raw (left) and DeepSeMi-enhanced (right) four-color cellular imaging in low-light conditions, with trajectories of a rod-shaped mitochondria tracked and enlarged (inset); color-coded timestamps are labeled at the bottom. Scale bar, 5 µm. *n* = 14 cells examined over three independent experiments. **f**, Displacement of the rod-shaped mitochondria plotted as a function of time. **g**, Inferred mitochondria displacements versus time under different imaging frame rates. Different colors represent different relative states of rod-shaped mitochondria to the cell body. Red arrows mark differences between displacement inferences of full sampling rate (7.5 Hz) and tenfold subsampling rate (0.75 Hz). **h**, Tracked drifting distances of mitochondria during 500 s with full sampling rate (7.5 Hz) and tenfold subsampling rate (0.75 Hz). **i**,**j**, Distributions of the moving rates and displacements of tracked rod-shaped mitochondria during leaving (**i**) and approaching (**j**) states, respectively.

Encouraged by the apparent SNR enhancement of DeepSeMi under sample-friendly power dosage across thousands of captures, we performed imaging at 7.5 fps on L929 cells with four structures labeled by four colors (tagBFP-SKL, TOM20-GFP, SiT-mApple and WGA647 for peroxisomes, mitochondria, Golgi and migrasomes, respectively) on a commercial confocal microscope (Fig. 3a; Methods) for 30 min and over 13,500 timepoints. Excitation power was set at 2% to avoid photobleaching and keep live cells healthy (Fig. 3b), at the expense of the extensive noise and ruptured structures that marred the raw captures. In contrast, the enhancement of DeepSeMi clearly revealed delicate structures of punctate peroxisomes, threadlike mitochondria and fluctuated membranes (Supplementary Video 3). Mitochondrial fission and fusion were clearly distinguished (Fig. 3c,d), highlighting the importance of combining minimization of illumination photon dose with SNR enhancement of DeepSeMi.

Together with its high temporal resolution and long-term capability, DeepSeMi opens up new possibilities in tracking the subtle movements of mitochondria. An individual rod-shaped mitochondrion was tracked based on DeepSeMi-enhanced recordings over 500 s, unveiling complicated trajectories and nonlinear movements (Fig. 3e,f). Sampling the data at full temporal resolution revealed brief transitions between mitochondria leaving and approaching, and quick motions were seen when the leaving or approaching mitochondria paused temporally^45^ (Fig. 3g). Such transient processes cannot be captured if the sampling frequency drops by tenfold to 0.75 Hz, which was the compromised frame rate for a standard confocal microscope without DeepSeMi enhancement. We thereby demonstrated that the high temporal resolution enabled by DeepSeMi is indispensable to characterizing the true trajectories as complex movements between frames were likely to be missed when temporal resolution dropped (Fig. 3h). We measured mitochondria leaving and approaching rates of 0.53 μm s^–1^ and 0.46 μm s^–1^, respectively. Furthermore, when analyzing these rates as a function of the displacement of each leaving or approaching event (Fig. 3i,j), we found that long displacing events correlated with slow rates of leaving or approaching. There was a broader range of leaving rates compared with approaching rates during short displacing events, leading to diverse fluctuations in mitochondria displacement. Overall, the SNR enhancement of DeepSeMi markedly enlarged the available photon budget of an optical instrument without compromising visual quality for downstream analysis. DeepSeMi allowed us to quantify not only dynamic mitochondrial displacement but also alteration of other organelles on a much finer temporal scale than that achieved in previous methods.

### DeepSeMi enables monitoring migrasomes and retractosomes over a half day in their native states

The migrasome was recently recognized as an extracellular organelle that plays a significant role in various physiological processes, including mitochondrial quality control, organ morphogenesis and cell interaction^46,47^. Despite fruitful results related to migrasome regulation revealed by light microscopy, observing migrasomes without interruption during cell migration in a half-day-long period remains challenging, being limited by continuous imaging-induced photobleaching and phototoxicity (Supplementary Fig. 35).

**Fig. 4:**
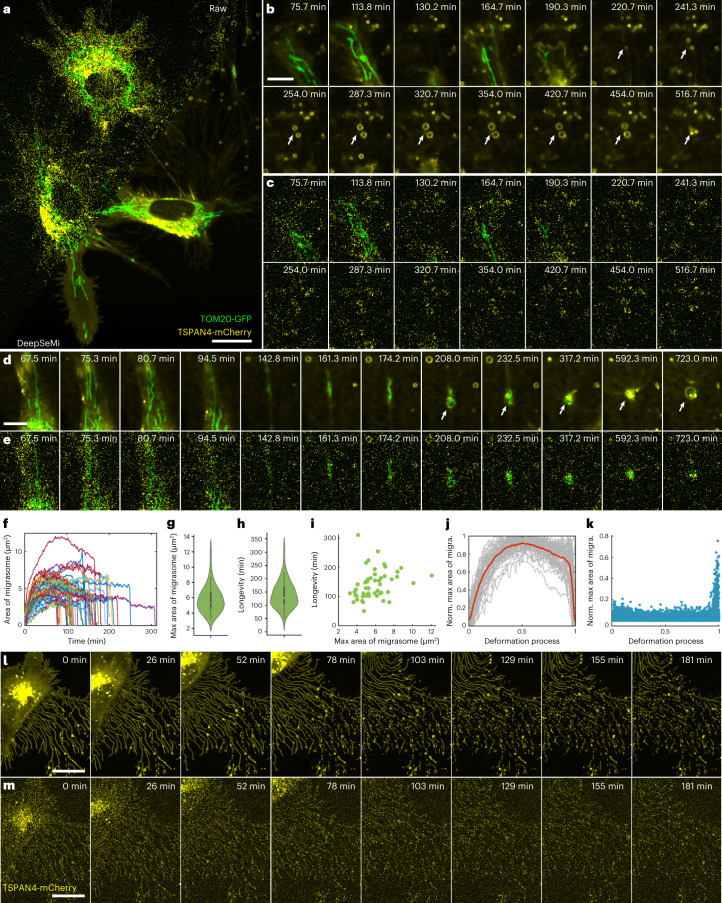
DeepSeMi enables half-day-long observations of migrasomes and retractosomes with low phototoxicity. **a**, Raw (top left) and DeepSeMi-enhanced (bottom right) micrograph of L929 cells expressing both TOM20-GFP and TSPAN4-mCherry. Scale bar, 20 μm. *n* = 5 cells examined over five independent experiments. **b**,**c**, Higher magnification panels visualizing extracellular migrasome generation and displacement by raw (**b**) and DeepSeMi-enhanced (**c**) recordings. Migrasomes marked by white arrows burst at the end of their lives. Scale bar, 10 μm. *n* = 5 cells examined over five independent experiments. **d**,**e**, Higher magnification panels visualizing mitocytosis and displacement by raw (**d**) and DeepSeMi-enhanced (**e**) recordings, respectively. Scale bar, 10 μm. **f**, Areas of extracellular migrasomes changing with time in DeepSeMi-enhanced videos. Different colors represent different migrasomes (*n* = 51). **g**, Violin plot of the maximum area of extracellular migrasomes in DeepSeMi-enhanced videos. White circle, median; thin vertical lines, upper and lower proximal values. Violin-shaped area: kernel density estimates of data distribution. *n* = 51 datapoints. **h**, Violin plot of the longevity of extracellular migrasomes in DeepSeMi-enhanced videos. Symbols as in **g**; *n* = 51 datapoints. Central black mark, median; bottom and top edges, 25th and 75th percentiles; whiskers extend to extreme points excluding outliers (1.5 times above or below the interquartile range). **i**, Scatter plot of longevity and maximum area of extracellular migrasomes in DeepSeMi-enhanced videos; *n* = 51 datapoints. **j**, Statistics of the normalized migrasome area changing across the migrasomes lifespan. Gray curves, trend of each migrasome (*n* = 51); red curve, average. **k**, Histogram of the area changing rate of migrasomes across *n* = 51 migrasomes. **l**,**m**, Generation of retractosomes in regions through which cells have migrated. A global view where the first row represents images enhanced by DeepSeMi and the second row represents the raw images. Scale bar, 20 μm. *n* = 3 cells examined over three independent experiments.

Here, through DeepSeMi enhancement, we accomplished high-resolution 2 fps imaging of the generation, growth and rupture of migrasomes in a half-day-long term with more than 86,000 timepoints with only 2% power shots (45.3 μW of 488 nm, 49.8 μW of 561 nm). A representative two-color image frame from a video of the mitochondria and migrasomes clearly showed the enormous SNR enhancement by DeepSeMi compared with raw capture (Fig. 4a and Supplementary Video 4). Near the cell body, DeepSeMi enabled us to find migrasomes that presented the entire generation and growth procedure across ∼300 min of imaging windows, which was 41% of the whole imaging session (Fig. 4b). The DeepSeMi-enhanced results clearly show that some mitochondria were expelled by the cell and kept inside a migrasomes (Fig. 4d,e), known as the mitocytosis^46^. Compared with barely recognized migrasomes in the raw images (Fig. 4c), 51 migrasomes were segmented from the whole DeepSeMi-enhanced capture (Methods), with color-coded area and longevity statistics summarized in Fig. 4f. We measured an averaged maximum area of 5.81 μm^2^ (Fig. 4g) during an average 141-min migrasome lifespan (Fig. 4h), which were weakly correlated with each other (Fig. 4i). We noticed a general pattern of the maximum area across those migrasomes consisting of a quick increase representing growth, a slightly declined plateau and a sharp drop representing rupture (Fig. 4j). The dynamics of rupture were much faster than the other two processes (Fig. 4k), necessitating DeepSeMi-enabled high temporal resolution and uninterrupted capture across a long time period to catch these features.

The retractosome was reported recently as a new type of small extracellular vesicle that is generated from broken-off retraction fibers and related closely to cell migrations^48^. Since uninterrupted cell migrations can be imaged continuously, benefiting from DeepSeMi-enabled low phototoxicity, high-SNR and long-term recording ability, retractosomes that were transformed from broken-off retraction fibers were clearly recognized (Fig. 4l,m). Although the beads-on-a-string features were indistinguishable in the raw captured video, retractosomes were clearly recognized when they moved along with the wobbly retraction fibers (Supplementary Video 5). After the cell migrated away, plenty of retraction fibers and retractosomes were left behind, forming a complicated network structure that appeared fractured due to signal noise. DeepSeMi reunited the network by wiping out noise contamination, thus delivering the potential to study the physiological functions of retractosomes in the future.

### DeepSeMi facilitates automated analysis of cellular structures from massive data

**Fig. 5:**
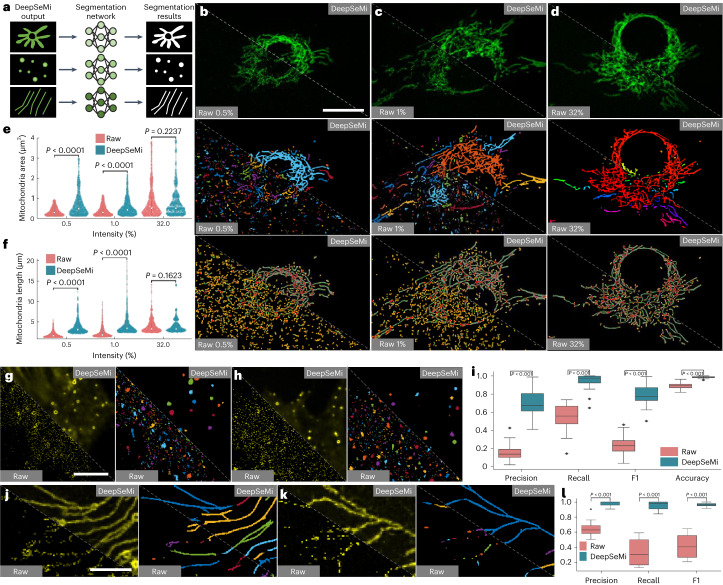
DeepSeMi facilitates accurate automated analysis of cellular structures with low light dosage. **a**, Schematic diagram illustrating the segmentation of mitochondria, migrasomes and retraction fibers through three neural networks (Methods). **b**–**d**, Differences in mitochondrial analysis based on raw images (bottom left) and DeepSeMi-enhanced (top right) images decrease as power dosage increases (**b** for 0.5% power, **c** for 1% power and **d** for 32%). The first row represents the raw captures (bottom left) and the DeepSeMi-enhanced fluorescence images (top right). The second row represents the instance segmentation of the raw captures (bottom left) and the enhanced images (top right). The third row represents the skeletonization of the raw captured mitochondria (bottom left) and the enhanced mitochondria (top right). Scale bar, 20 μm. **e**, Statistics of mitochondria area based on the instance segmentation before (red) and after (blue) DeepSeMi enhancement. White dots, median; thin vertical lines, upper and lower proximal values; violin-shaped area, kernel density estimates of data distribution. Two-sided Wilcoxon signed-rank test; *n* = 1,000 images per intensity. *n* = 10 cells examined over two independent experiments. **f**, Statistics of branch length of mitochondria based on the skeletonization before (red) and after (blue) DeepSeMi enhancement. Symbols as in **e**. Two-sided Wilcoxon signed-rank test; *n* = 1,000 images per intensity. **g**,**h**, Instance segmentation of migrasomes before (bottom left) and after (top right) DeepSeMi enhancement. Scale bar, 20 μm. **i**, Segmentation precision, recall, F1 score and accuracy scores of the migrasomes before (red) and after (blue) DeepSeMi enhancement. Groundtruth data were annotated manually (Methods). Two-sided Wilcoxon signed-rank test; *n* = 32 images. **j**,**k**, Instances (**j**,**k**) segmentation of retraction fibers before (bottom left) and after DeepSeMi enhancement (top right). Scale bar, 10 μm. **l**, Segmentation precision, recall, F1 scores of the retraction fibers before (red) and after DeepSeMi enhancement (blue). Groundtruth data is manually annotated (Methods). Two-sided Wilcoxon signed-rank test; *n* = 12 images. In **i** and **l**, central black mark, median; bottom and top edges, 25th and 75th percentiles; whiskers extend to extreme points excluding outliers (1.5 times above or below the interquartile range).

Uncovering the peculiarities of important life-preserving and disease-driving organelles requires robust and unbiased segmentation and tracking tools. Given the growing requirement for long-term recordings and attendant generation of considerable amounts of cellular imaging data measured in hundreds of gigabytes^49^, automated cellular analysis is becoming indispensable for new physiological discoveries. Here, we verify the compatibility of DeepSeMi with cutting-edge automated segmentation tools^50^. We trained three segmentation networks for mitochondria, migrasomes and retraction fibers (Fig. 5a; Methods). We found that raw captures of mitochondria under 14.6 μW (0.5% of 488 nm)—a bio-friendly power dosage—suffered pronounced segmentation errors due to noise contamination (Fig. 5b–d and Supplementary Fig. 36). Incorrect segmentation fragments in the background were eliminated only when the power dosage was pushed into 537.6 μW (32% of 488 nm), at a cost of significant photobleaching (Fig. 5b–d and Supplementary Fig. 28h). By contrast, DeepSeMi enhancement enabled the segmentation model to produce reasonable and gap-free results even at 14.6 μW (0.5% of 488 nm) (Fig. 5b and Supplementary Fig. 36), permitting reliable segmentation during long-term imaging thanks to heavily reduced photobleaching. Through additionally performing mitochondrial skeletonization and keypoint detection based on instance segmentation^17^ (Supplementary Fig. 37), we found that the markedly noisy areas in raw captures were recognized as endpoints and junctions of broken skeletons (Fig. 5b and Supplementary Fig. 36). These false positives were well avoided in DeepSeMi-enhanced results, and the skeletonization result produced by DeepSeMi at 14.6 μW (0.5% of 488 nm) is comparable with that in the raw image at 537.6 μW (32% of 488 nm). Quantitively, DeepSeMi-enhanced videography achieved significantly larger mitochondrial area (*P* < 0.0001, two-sided Wilcoxon rank sum test; Fig. 5e and Supplementary Fig. 36; Methods) and longer branch length (*P* < 0.0001, two-sided Wilcoxon rank sum test; Fig. 5f and Supplementary Fig. 36; Methods) compared with those based on raw data at a sample-friendly power dosage (14.6 μW (0.5% of 488 nm). These statistics were comparable only when the power reaches the harmful level of 537.6 μW (32% of 488 nm; *P* > 0.1, two-sided Wilcoxon rank sum test). The >15-fold power reduction of DeepSeMi in achieving high-quality subcellular segmentation validated with >15 times greater photon budget compared with a previous photobleaching study Supplementary Fig. 28), together indicate the strong advantages of DeepSeMi over optical instrument in terms of being bio-friendly, resolving ability and data fidelity.

To further evaluate the improvement of segmentation accuracy brought by DeepSeMi enhancement, we segmented migrasomes and retraction fibers manually as the groundtruth and compared the results with automated segmentations on DeepSeMi-enhanced videography (Methods). DeepSeMi apparently achieved much clearer micrographs and hence cleaner segmentations (Fig. 5g,h). Statistically, DeepSeMi enhancement achieved 0.9449 ± 0.0782 recalls (*n* = 32 images) in migrasome segmentations, holding a safe advantage compared with raw-video-based segmentation (0.5522 ± 0.1359 recalls, *n* = 32 images). The same advantages were held in segmenting string-like retraction fibers (Fig. 5j,k), where DeepSeMi enhancement achieved 0.9493 ± 0.0618 recalls (*n* = 12 images) compared with 0.3391 ± 0.1848 recalls by raw video (*n* = 12 images; Fig. 5l). We subsequently substantiated the enhancement in segmentation accuracy conferred by DeepSeMi using our simultaneous high- and low-SNR imaging system. We observed that DeepSeMi outperformed other benchmarked denoising methodologies, as evident from several segmentation metrics including accuracy, F1 score, intersection over union and recall (Supplementary Fig. 38). Furthermore, DeepSeMi demonstrated an unwavering consistency in delivering high-performance cellular segmentation across various imaging speeds (Supplementary Fig. 39). The high segmentation accuracy enabled by DeepSeMi under sample-friendly power dosage would be the key to massive data analysis through automated algorithms after long-term recordings.

### DeepSeMi accomplishes SNR enhancement across various samples

**Fig. 6:**
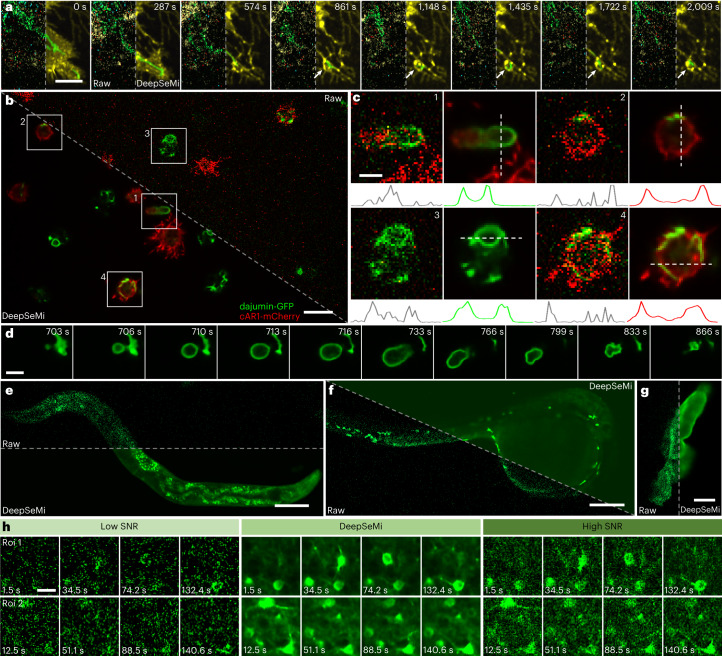
DeepSeMi seamlessly improves SNRs over various species. **a**, Generation of a migrasome from the L929 cell with four organelles labeled with different colors (TOM20-GFP, WGA647, TagBFP-SKL and SiT-mApple; Supplementary Fig. 40) after treatment with lat-A (0.5 μg ml^–1^; Methods). For each panel, the right part represents DeepSeMi-enhanced results and the left panel represents the raw image. Scale bar, 10 μm. *n* = 12 cells examined over three independent experiments. **b**, Raw (top right) and DeepSeMi-enhanced (bottom left) long-term high-speed imaging of photosensitive *Dictyostelium* cells. Scale bar, 10 μm. *n* = 36 cells examined over four independent experiments. **c**, Enlarged images of the white boxes marked 1–4 in **b** representing contractile vacuoles and membranes. Intensity profiles along the white dashed lines are plotted at the bottom. Scale bar, 3 μm. *n* = 36 cells examined over four independent experiments. **d**, Timelapse imaging of expansion and contraction of the contractile vacuole enhanced by DeepSeMi. Scale bar, 4 μm. *n* = 36 cells examined over four independent experiments. **e**, In vivo imaging of *C.* *elegans* in a millimeter-scale field-of-view by raw (top) and DeepSeMi-enhanced (bottom) captures. Scale bar, 100 μm. *n* = 8 examined over two independent experiments. **f**, In vivo imaging of zebrafish larvae in a millimeter-scale field-of-view by raw (bottom left) and DeepSeMi-enhanced (top right) captures. Scale bar, 200 μm. *n* = 12 examined over four independent experiments. **g**, Observation of macrophage in zebrafish larvae in vivo by raw (left) and DeepSeMi-enhanced (right) images, respectively. Scale bar, 5 μm. *n* = 12 examined over four independent experiments. **h**, Low-SNR (left), DeepSeMi-restored (middle) and high-SNR (right) images recorded by tenfold higher photon flux as references. Low-SNR and high-SNR images were recorded through a hybrid multi-SNR two-photon system for validation^34^. Eight timepoints are displayed for each modality. Roi 1 and Roi 2 were two regions from a recording of GGaMP6f-labeled neurons in in vivo mouse cortex. Scale bar, 20 μm. *n* = 12 examined over six independent experiments.

Last, we demonstrated that DeepSeMi effectively increases SNRs across various samples, including cultured cells, unicellular organisms, nematodes, nonmammalian vertebrates and mammals. We have demonstrated DeepSeMi-enabled high-temporal-resolution imaging of mitochondria, low phototoxicity half-day-long imaging of migrasomes and retractosomes, and facilitated automated analysis in massive data under biofriendly illumination dosage, but the power of DeepSeMi could be extended further. We next demonstrated that DeepSeMi can be used to study the rearrangement of organelles after disrupting the cytoskeleton and other organelle-related structures. By dosing an appropriate concentration of latrunculin-A (lat-A) to induce the depolymerization of the intracellular actin cytoskeleton, a new spatial distribution of intracellular organelles was formed (Supplementary Fig. 40). We found the migrasomes were generated following the rapid contraction of the cell membrane after depolymerization of the cytoskeleton (Fig. 6a). All those observations relied on the enhancement of DeepSeMi, which restored mitochondria and other organelles of diverse morphologies from noise. Similar improvements happened in the study of vesicle fission (Supplementary Fig. 19h and Supplementary Video 1), where kymographs (*x*–*t* projections) clearly presented the enhancements of DeepSeMi (Supplementary Fig. 19i), and also in the study of migrating cell interacting with a migrasome (Supplementary Fig. 41b), producing migrasomes (Supplementary Fig. 41c) and expelling mitochondria in low light dosage (Supplementary Fig. 41d and Supplementary Video 6).

DeepSeMi also ena bled high-SNR, half-hour-long imaging of cells from *Dictyostelium*—an important amoeba-like eukaryote model for studying genetics, cell biology and biochemistry^51^. Despite the great value of *Dictyostelium* cells in research, it is ultrasensitive to photodamage; 215 μW of laser dosage at 638 nm and 50.6 μW of laser dosage at 561 nm killed 30% of *D.* *discoideum* after 30-min imaging, preventing high-SNR long-term imaging using conventional approaches (Supplementary Figs. 42 and 43). We applied DeepSeMi to circumvent the problem, which enabled dual-color and high-SNR imaging at the 45.3 μW dosage at 488 nm and the 49.8 μW dosage at 561 nm over 30 min without apparent photodamage (Fig. 6b and Supplementary Figs. 42 and 44). Contractile vacuoles and membranes of *Dictyostelium* cells were easily recognized with clear boundaries through DeepSeMi enhancement (Fig. 6c and Supplementary Fig. 45), and the uninterrupted videography enabled by DeepSeMi unveiled startling images of *Dictyostelium* cell motions such as contracting (Fig. 6d and Supplementary Video 7). The ability of DeepSeMi to greatly improve SNR without increasing power dosage will shed new light on photodamage-sensitive but valuable animal models like *Dictyostelium*.

*Caenorhabditis elegans* and zebrafish are used as central model systems across many biological disciplines^52,53^. The rather scattered tissues of *C.* *elegans* exaggerate noise contamination even more than cultured cells (Fig. 6e and Supplementary Fig. 46a); DeepSeMi substantially improved the contrast and sharpness of cell images (Supplementary Fig. 46b–f). Although utilizing a higher numerical aperture (NA) objective results in even greater scattering, DeepSeMi restores delicate structures with sharp edges and high contrast from noise (Supplementary Fig. 46g–j). On the other hand, the transparency of zebrafish larvae not only helps better observation of structures and functions of cells and organisms in vivo, but also eliminates the protective barrier to photodamage during optical observation^54^. Thereby, imaging zebrafish larvae necessitates low illumination power to avoid affecting the health and normal physiological regulation of the sample, which inevitably raises challenges from noise contamination (Fig. 6f and Supplementary Fig. 47a). We proved that DeepSeMi enhancement solved this dilemma and provided a clear view of macrophage in zebrafish larvae under a mild power dosage (45.3 μW; Fig. 6g and Supplementary Fig. 47b,c), showing the potential for long-term observations for studying development and function in a highly complex vertebrate model system.

DeepSeMi is also demonstrated to be operative in functional imaging in mice, which are widely used in systems and evolutionary neuroscience. We tested the generalization of DeepSeMi in nonlinear microscopy, where neurons were excited sequentially by a focused femtosecond laser in vivo. DeepSeMi readily enhanced visualization of morphologies of neuronal structures (Fig. 6h and Supplementary Figs. 48a–c and 49a–i) from barely recognizable noisy captures, and also demonstrably increased the temporal contrast of calcium transients (Supplementary Figs. 48d and 49j). Videos denoised by DeepSeMi found 1.5 times more neurons, which could impel potential interrogation of neuronal circuits (Supplementary Figs. 48e and 49k). For observing even smaller structures, such as wobbled neuronal dendrites and axons in vivo in the mouse brain, the enhancement brought by DeepSeMi has no equal (Supplementary Fig. 50).

## Discussion

The ability to image live biological specimens over time with high spatiotemporal resolution and low photodamage will be of great scientific value. To improve such imaging, we present DeepSeMi, a versatile self-supervised paradigm capable of enhancing SNR over 12 dB, improving photon budget 15-fold and reducing fluorescent dye concentration 50-fold across various samples and instruments with only noisy images required as input. DeepSeMi features specially designed receptive field-limited convolutional filters that readily accomplish noise contamination removal without clean data reference or interframe interpolations, achieving superior performance over other methods, especially in data with complicated transformation. The computationally enhanced photon budget produced by DeepSeMi enabled high-frame-rate four-color organelle recordings across tens of thousands of frames, allowing the tracking of migrasomes and retractosomes over a half day, and the long-term imaging of ultra-photodamage-sensitive *D.* *discoideum* with high fidelity. Moreover, DeepSeMi was proven to help the automated analysis of cells and organelles, which is a strong aid in processing massive imaging data. The performance of DeepSeMi on various species, including nematodes, zebrafish and mice, on both widefield and two-photon microscopes was also validated both qualitatively and quantitatively. In conclusion, DeepSeMi offers a combination of high-resolution, high-speed, multicolor imaging and low photobleaching and phototoxicity that makes it well suited to studying intracellular dynamics and more.

As a fundamental limitation in fluorescence imaging, stochastic noise determines the upper boundary of imaging quality and compromises speed, resolution and sample health across any instrument. The proposed DeepSeMi can be extended seamlessly to various devices that most suffer from noise, including the three-photon microscope, which has as ultra-small absorption cross-section^55^, and the Raman microscope with critical excitation conditions^56^. In other devices, such as widefield and lightfield microscopes where background contaminates more to scattering tissues than noise, DeepSeMi can collaborate with computational background elimination methods^57^ to jointly improve imaging quality with rejected background and increased SNR.

The rearrangement of computationally multiplied photon budgets by DeepSeMi can be more diverse. We have shown the benefits of shortened exposure, which supports a higher frame rate for interrogating fast dynamics (Fig. 3), and reduced frame rate, which enables longer recording time for investigating long-term variations (Fig. 4). Furthermore, the temporal resolution of an optical system can be further enhanced without losing spatial resolution through combination with multiplexing techniques^58^, and DeepSeMi readily mitigates the photodamage caused by excessive power dosage. When pushing the frame rate to the limit, a standard device may be capable of imaging ultrafast phenomena like spiking^59,60^ and flagellar locomotion^61^ without losing fidelity by using DeepSeMi.

Although basic exploration of DeepSeMi has been examined in this manuscript, continued diverse research could further increase its accessibility. Combining DeepSeMi with advanced model compression and pruning techniques^59^, will further compress the computation time of DeepSeMi for high-speed data inference. Training DeepSeMi across a large range of conditions with varied noise and transformations over several samples forms a general model and, in specific conditions, DeepSeMi can be distributed swiftly from the basic system to one with fine-tuning and better performance^62^.

In short, we believe DeepSeMi provides a robust solution to overcome the shot-noise limitation in fluorescent microscopy. With the computational enhancement of DeepSeMi, various organelles and organisms can be recorded safely over long periods at high spatiotemporal resolution, bringing fresh insight to new physiological discoveries.

## Methods

### Network structure

DeepSeMi consisted of six three-dimensional (3D) hybrid blind-spot neural networks (four spatial blind-spot networks and two temporal blind-spot networks) and one FFnet (Supplementary Fig. 5). All six hybrid blind-spot networks had the same U-net-like structure for extracting features from input videos. Each hybrid blind-spot network consisted of 14 3D convolution layers. The first two layers were 3D eccentric blind-spot convolutional layers with 3 × 3 × 3 sized kernels (Fig. 1c). The encoding path of DeepSeMi was composed of alternate 3D eccentric blind-spot convolutional layers (3 × 3 × 3 sized kernels) and MaxPooling layers (2 × 2 × 2). Similarly, the decoding path was implemented by alternate 3D eccentric convolutional layers (3 × 3 × 3 sized kernels) and Upsampling layers (2 × 2 × 2). The numbers of input and output features in each layer were set to 32 to accommodate single-graphics-processing-unit training. The FFnet consisted of three 3D convolutional layers with 1 × 1 × 1 kernels. The number of input channels of the FFnet was 32 × 6 = 192 to match the size of concatenated features of the six branch networks, whereas the number of output channels of the FFnet matches the real image and depends on the experiment. The loss function of DeepSeMi was a summation of l1 norm and l2 norm, and the learning rate was set to 0.0001.

We usually picked up 1,000 patches from noisy videos to form the training set, and the size of each patch was 128 × 128 × 32. Good convergence could be obtained usually after 30–50 epochs of training. The entire training process took about 6 h on an NVIDIA 3090 Ti graphics card.

### Eccentric blind-spot convolution and eccentric convolution

Eccentric blind-spot convolution stemming from traditional convolutions plays a significant role in DeepSeMi. Here, we illustrate the concept of eccentric blind-spot convolution through derivations. To simplify the description, all following operations are derived in two dimensions, while 3D operations can be extended easily.

The traditional discrete convolution (Supplementary Fig. 1a) can be formulated as:

$${y}_{m,n}=\mathop{\sum}\limits_{i=-h}^{h}\mathop{\sum}\limits_{j=-h}^{h}{x}_{m-i,n-j} {k}_{h-i+1,h-j+1}$$

where *y* is the output of the convolution, *x* is the input image, *k* is the kernel of convolution with a size of [2*h* + 1, 2*h* + 1], *m* and *n* are the two-dimensional (2D) index of a pixel in the image, *h* is used to describe the size of the convolution kernel, and *i* and *j* are variables of discrete convolution. Note the information of input pixel *x*_*m*,*n*_ will be transmitted to the output pixel *y*_*m*,*n*_ in the above traditional convolution process when *i* = 0 and *j* = 0, resulting the noise of input pixel *x*_*m*,*n*_ will also be kept at the output pixel *y*_*m*,*n*_. Training a neural network composed of such convolutional layers in noise-only data will generate trivial results with the identified mapping, and only noisy-clean data pairs or sequential noisy acquisitions can fuel that neural network with the deficiency of self-supervision. To give the neural network the ability to self-supervise denoising, we construct an eccentric blind-spot convolution kernel (Supplementary Fig. 1c), which can be formulated as:

$${y}_{m,n}=\mathop{\sum}\limits_{i=-h}^{h}\mathop{\sum}\limits_{j=-h}^{h}{x}_{m-i+h+1,n-j} {k}_{h-i+1,h-j+1}$$

where the symbols are the same as the above equation. With the proposed eccentric blind-spot convolution, the noisy information of input pixel *x*_*m*,*n*_ will not be conserved in the output pixel *y*_*m*,*n*_, and information of the output pixel *y*_*m*,*n*_ can be estimated only from local pixels around the input pixel *x*_*m*,*n*_.

Next, we derive the proposed eccentric convolutional filter and explain why it is important to DeepSeMi. We found that, when directly combining the aforementioned eccentric blind-spot convolution kernels with traditional convolutional kernels, the blind-spot properties that are key to ensuring self-supervision would be lost. To illustrate that, we concatenate a 2D eccentric blind-spot convolution and a 2D traditional convolution:

$${y}_{m,n}=\mathop{\sum}\limits_{i=-h}^{h}\mathop{\sum}\limits_{j=-h}^{h}{x}_{m-i+h+1,n-j} {k}_{h-i+1,h-j+1}^{1}$$

$${z}_{m,n}=\mathop{\sum}\limits_{i=-h}^{h}\mathop{\sum}\limits_{j=-h}^{h}{y}_{m-i,n-j} {k}_{h-i+1,h-j+1}^{2}$$

where *x* is the input, *y* is the intermediate variable from the eccentric blind-spot convolutional kernel *k*^1^ and *z* is the output from the traditional convolutional kernel *k*^2^. Both kernels are with size [2*h* + 1, 2*h* + 1]. It can be easily found that, when *h* > 0, if

$${k}_{a,b}^{1}=\left\{\begin{array}{l}1,\,a=1{\rm{and}}\; {\rm{b}}=h+1\\ \quad 0,{\rm{others}}\end{array}\right.$$

and

$${k}_{a,b}^{2}=\left\{\begin{array}{l}1, \, a={h\; {\rm{and}}\; {\rm{b}}}=h+1\\ \quad 0, \, {\rm{others}}\end{array}\right.$$

the above formula can be simplified to:

$${y}_{m,n}={x}_{m+1,n}$$

$${z}_{m,n}={y}_{m-1,n}$$

This is equivalent to:

$${z}_{m,n}={y}_{m-1,n}={x}_{m,n}$$

In other words, the original noise pixel *x*_*m*,*n*_ is mapped directly onto an output pixel *z*_*m*,*n*_ with the same position, indicating that the blind-spot properties are dropped. The above examples are illustrated in Supplementary Figs. 2 and 3. In the extreme condition *h* = 0, such blind-spot properties can be still held, explaining why we utilized 3D convolutions with kernel size 1 × 1 × 1 in the FFnet.

To circumvent this shortage, we designed another eccentric convolution which can be formulated as:

$${y}_{m,n}=\mathop{\sum}\limits_{i=-h}^{h}\mathop{\sum}\limits_{j=-h}^{h}{x}_{m-i+h,n-j} {k}_{h-i+1,h-j+1}^{1}$$

Following similar derivations as shown above, it can be proved that the blind-spot properties are retained in the combination of fully blind convolutions and eccentric convolutions.

Although the introduction of blind-spot convolutional kernels enabled the neural network to learn denoising without excessive data, the receptive field is limited to only one direction for both the kernels and kernels composited networks (Supplementary Fig. 2). We thus established the hybrid blind-spot network as several branches to extract features from different directions, and then fuse these features by FFnet to achieve the all-direction-received output result.

### Time-to-feature operation

We inserted a time-to-feature operation at the beginning of the input of the neural network for inputting more temporal information but without noticeably increasing computing time. To achieve that, twice as many input frames were added to the network and stacked in the channel dimensions instead of temporal dimensions, which can be squeezed quickly after interacting with the next convolutional kernel. As an example, when a video block with a size of C × (T + 2 × F) × H × W was desired to be input, we realigned it to a tensor of size (2 × F × C + C) × T × H × W by multiplexing some frames as the real input of the DeepSeMi, where C is the channel number of each frame from the video block, T is the length of the video block output by the neural network, F is the number of additional frames fed into the neural network and H is the height of the video block. W is the width of the video block.

### Generation of simulated motion datasets

To fully compare the denoising performance of different algorithms on the video denoising task, we utilized the Moving MNIST dataset, which is used widely in the field of computer vision, as the simulated dataset. The images from the MNIST handwritten digit database served as the main moving contents in generated videos, while each frame is 256 × 256 pixels in size. In the beginning, we randomly selected ten handwritten digits to form the basic content, and generated random motions for each of the digits. Then, the whole video was generated frame by frame by keeping shifting the digits in predefined tracks. To keep the handwritten digits within the bounds of the video frame, the handwritten digit bounced at the edges of the video frame. The size of the video we usually generate was 256 × 256 × 1,000 pixels.

### Noise simulation and analysis

We evaluated the performance of DeepSeMi in both Gaussian noise and Poisson noise. Gaussian noise was simulated by dataset by the *getExperimentNoise* function derived from the blind denoising method BM3D with varied noise scales. The Poisson noise was simulated by the *MPG_model* function derived from DeepCAD^34^. We utilized several indicators to evaluate the noise scale. Peak SNR is used widely for measuring the similarity between recovered images and paired groundtruth images. Peak SNR (in dB) is calculated as:

$${\rm{PSNR}}=10\times {\log }_{10}\left(\frac{{{\rm{MAX}}}_{I}^{2}}{\tfrac{1}{{n}_{1}{n}_{2}}{\sum }_{i}^{{n}_{1}}{\sum }_{j}^{{n}_{2}}{({I}_{i,\,j}-{X}_{i,\,j})}^{2}}\right)$$

where *X* is a *n*_1_ × *n*_2_ recovered image, I is the paired noise-free image. *MAX*_*I*_ is set to 65,535 for 16-bit unsigned integer images. SNR was also selected to quantify the image quality after denoising. SNR (in dB) is calculated as:

$${\rm{SNR}}=10\times {\log }_{10}\left(\frac{{\sum }_{i}^{{n}_{1}}{\sum }_{j}^{{n}_{2}}{{X}_{i,\,j}}^{2}}{{\sum }_{i}^{{n}_{1}}{\sum }_{j}^{{n}_{2}}{({I}_{i,\,j}-{X}_{i,\,j})}^{2}}\right)$$

### Evaluation of photobleaching

Photobleaching represents the inability of a fluorescent protein to emit photons after continuous excitation. To evaluate photobleaching under different power dosage conditions, we averaged all pixel intensities from the acquired image. To eliminate the influence of sensor background noise even without the input of fluorescence photons, we calculated the averaged intensity in a sample-free area, and updated the averaged intensity accordingly across the whole image such that it represents net fluorescence photon flux. We then quantified the speed of photobleaching by fitting the photobleaching curve using an exponential function.

### Training of organelle segmentation network

As the demand for studying cell biology through microscopic fluorescence imaging increases, it is necessary to utilize automated analysis tools to process massive imaging data in a relatively short time to enrich quick experiment iterations. We demonstrated that DeepSeMi enhances automated analysis of organelles with high precision and low phototoxicity. We utilized a physics-based machine learning method for organelle segmentation^50^. We simulated both optical imaging results and segmented groundtruth of mitochondria, migrasomes and retraction fibers based on the morphological characteristics. A total of 1,500 paired images were prepared for each organelle. We then built and trained a traditional 2D U-net using the simulated datasets, with the size of the input image of 256 × 256 pixels. It took about 10 min on an NVIDIA 3080 Ti graphics card to achieve good convergence results in about four to ten epochs. The learning rate was set to 0.0001.

We utilized merits of precision, recall, F1 score and accuracy for segmentation evaluation of the network:

$${\rm{Precision}}=\frac{{\rm{TP}}}{{\rm{TP}}+{\rm{FP}}}$$

$${\rm{Recall}}=\frac{{\rm{TP}}}{{\rm{TP}}+{\rm{FN}}}$$

$$\rm{F1}\,{\rm{score}}=\frac{2{\rm{TP}}}{2{\rm{TP}}+{\rm{FN}}+{\rm{FP}}}$$

$${\rm{Accuracy}}=\frac{{\rm{TP}}+{\rm{TN}}}{{\rm{TP}}+{\rm{TN}}+{\rm{FP}}+{\rm{FN}}}$$

where TP is true positive, TN is true negative, FP is false positive and FN is false negative.

### Mitochondrial analysis

After mitochondrial segmentation through the methods described above, the connected regions from the segmented binary masks were detected using the bwlabel function in MATLAB to accomplish mitochondrial instance segmentation. The mitochondrial area of each connected region was calculated, and the skeletons and key points of mitochondria were picked up through the bwmorph function in MATLAB. According to the different topological positions, the key points were classified into junctions or end points. We tracked the mitochondria with Imaris (Oxford Instruments) across recording sessions to indicate the movement state of mitochondria.

### Cell culture and imaging system

L929 cells and NRK cells were cultured in DMEM (Gibco) medium supplemented with 10% FBS (Biological Industries), 2 mM GlutaMAX and 100 U ml^–1^ penicillin-streptomycin in 5% CO_2_ at 37 °C. The PiggyBac Transposon Vector System was used to generate the stably expressing cell line. For L929 cells, Vigofect was used for cell transfection according to the manufacturer’s manual. NRK cell transfection was via Amaxa nucleofection using solution T and program X-001. Confocal dishes (35 mm) were precoated with fibronectin (10 mg ml^–1^) at 37 °C for 1 h. Cells were cultured in fibronectin-precoated confocal dishes for 4 h before imaging. AX2 axenic strain cells were provided by the Jeffrey G. Williams laboratory (University of Dundee). AX2 wild-type cells and the derived cell line were cultured in HL5 medium (Formedium, catalog no. HLF2), supplemented with antibiotics, at 22 °C. Plasmids pDM323 and pDM451 were provided by the Huaqing Cai laboratory (Chinese Academy of Sciences). DNA fragments encoding dajumin and cAR1 were PCR-amplified and cloned into the overexpressing plasmids.

*C.* *elegans* stably overexpressing OSM-3-GFP were provided by the Guangshuo Ou laboratory (Tsinghua University). We cultivated *C.* *elegans* on nematode growth medium agar plates seeded with the *Escherichia coli* OP50 at 20 °C. For live-cell imaging, worms were anesthetized with 1 mg ml^–1^ levamisole and mounted on 3% agarose pads at 20 °C.

*Tg(mpeg1.1:PLMT-eGFP-caax)* transgenic zebrafish were provided by B. Liu. All adult zebrafish were kept in a water-circulating system at 28.5 °C. Fertilized eggs were raised at 28.5 °C in Holtfreter’s solution. The embryos were embedded in 1% low-melting-point agarose for live-cell imaging. The use of all zebrafish adults and embryos was conducted according to the guidelines from the Animal Care and Use Committee of Tsinghua University.

All imaging experiments in this research were based on a Nikon A1 confocal microscope (Tsinghua University). All cellular imaging was conducted by a ×100 objective (NA 1.45, oil immersion). A ×10 objective (×10, NA 0.45, air) was used to capture the global image of *C.* *elegans* and zebrafish. Two-photon imaging was conducted with a customized two-photon imaging system under a commercial objective (×25, NA 1.05, XLPLN25XWMP2, Olympus).

### Calibration of the high- and low-SNR confocal system

For certifying the fluorescence intensity ratio between the images captured by the high-SNR and low-SNR detection paths, we imaged three kinds of fixed cell samples, labeled with Tom20-GFP, mOrange2-SKL and WGA647, respectively, for calibrating the system. To fairly compare the difference of the photon number collected by the two PMTs, we set the two PMTs at the same gain value to maintain equal photoelectric conversion efficiency. An imaging region was continuously scanned 200 times to obtain several imaging results of the same scene. To eliminate the influence of detection noise on calibration results, we averaged 200 frames to acquire a noise-free image of each PMT. We labeled signal and background regions manually on the final noise-free image. The net photon number was calculated by subtracting the background intensity from the total signal intensity. Based on our analysis, the photon number of the high-SNR detection path was about 15 times higher than that of low-SNR detection path.

### Compared methods

We compared denoising performance against six other blind denoising methods: bm3d, vbm3d, Noise2Self, UDVD, DeepInten and DeepCAD. For bm3d and vbm3d, we downloaded the Matlab code from https://webpages.tuni.fi/foi/GCF-BM3D/. For each denoising image, we searched the best hyperparameters for denoising by traversal. We set the sequence length of vbm3d to 32. For Noise2Self, we obtained the Python code from https://github.com/czbiohub/noise2self. The training set size is 10,000 and the learning rate is 0.00005. We selected the best denoised results from all epochs as the final result. For UDVD, we acquired the Python code from https://github.com/sreyas-mohan/udvd. The training set size is 2,000 and the learning rate is 0.0001. The input sequence length is 15. We selected the best denoised results from all epochs as the final result. For DeepInten, we obtained the Python code from https://zenodo.org/record/5165320. The training set size is 2,000 and the learning rate is 0.0001. The input sequence length is 33. We selected the best denoised results from all epochs as the final result. For DeepCAD, we obtained the Python code from https://github.com/cabooster/DeepCAD. The training set size is 1,000 and the learning rate is 0.0005. The input sequence length is 64. We selected the best denoised results from all epochs as the final result.

### Reporting summary

Further information on research design is available in the Nature Portfolio Reporting Summary linked to this article.

## Online content

Any methods, additional references, Nature Portfolio reporting summaries, source data, extended data, supplementary information, acknowledgements, peer review information; details of author contributions and competing interests; and statements of data and code availability are available at 10.1038/s41592-023-02058-9.

### Supplementary information

Supplementary Information Supplementary Figs. 1–50.

Reporting Summary

Supplementary Video 1 Evaluation and segmentation of DeepSeMi enhancement over a triple-color labeled L929 cell in low light.

Supplementary Video 2 Evaluation of photobleaching of mitochondria under different laser dosages.

Supplementary Video 3 Evaluation of DeepSeMi enhancement in a quadruple-color labeled L929 cell in low light over 13,000 frames.

Supplementary Video 4 Evaluation of DeepSeMi enhancement in observation of cell migrations in low light over 12 h.

Supplementary Video 5 Evaluation of DeepSeMi enhancement in observation of retractosomes generation.

Supplementary Video 6 Evaluation of DeepSeMi enhancement in observation of intercell interactions in low light over 2 h.

Supplementary Video 7 Evaluation of DeepSeMi enhancement in observation of *Dictyostelium* cells in low light.

## Data Availability

Our DeepSeMi datasets can be found at https://drive.google.com/drive/folders/1knd5Dpgl8C0zuHpgdkKkhev6lA-SN09t?usp=share_link.

## Code availability

Our DeepSeMi can be found at https://github.com/GuoxunZhang-PhD/DeepSeMi.
