# Supplementary material for: Bio-friendly long-term subcellular dynamic recording by self-supervised image enhancement microscopy
Source: Nat Methods. 2023 Nov 13;20(12):1957–70. doi: 10.1038/s41592-023-02058-9 (PMC10703694; doi:10.1038/s41592-023-02058-9)
Supplement: Supplementary file 1 — Supplementary Figs. 1–50. [file 41592_2023_2058_MOESM1_ESM.pdf]

nature methods

Article

https://doi.org/10.1038/s41592-023-02058-9

Bio-friendly long-term subcellular dynamic recording by self-supervised image enhancement microscopy

In the format provided by the authors and unedited

Bio-friendly long-term subcellular dynamic recording by self-supervised image enhancement microscopy

Guoxun Zhang[1,2*], Xiaopeng Li[3*], Yuanlong Zhang[1,2*], Xiaofei Han[1,2], Xinyang Li[1,2,4], Jinqiang Yu[3], Boqi Liu[3], Jiamin Wu[1,2†], Li Yu[3†] & Qionghai Dai[1,2†]

[1]*Department of Automation, Tsinghua University, Beijing, 100084, China*

[2]*Institute for Brain and Cognitive Sciences, Tsinghua University, Beijing 100084, China*

[3]*State Key Laboratory of Membrane Biology, Tsinghua University–Peking University Joint Centre for Life Sciences, Beijing Frontier Research Centre for Biological Structure, School of Life Sciences, Tsinghua University, Beijing, 100084, China*

[4]*Tsinghua Shenzhen International Graduate School, Tsinghua University, Shenzhen, China*

[*]*These authors contributed equally to this work*

[†]Correspondence: wujiamin@tsinghua.edu.cn (J.W.), liyulab@mail.tsinghua.edu.cn (L.Y.), qhdai@tsinghua.edu.cn (Q.D.)

Supplementary Figures

**Supplementary Figure 1**	Comparison of the proposed blind-spot convolutional filter with the traditional 2D convolution.
**Supplementary Figure 2**	Receptive field visualization of networks with various branches.
**Supplementary Figure 3**	Receptive field visualization of networks with various convolutional filters and various branches.
**Supplementary Figure 4**	Analysis of network characteristics with varying blind spots by evaluating their inability to learn a trivial identity map.
**Supplementary Figure 5**	The network structure of DeepSeMi.
**Supplementary Figure 6**	Ablation study of DeepSeMi in Moving MINIST datasets over different noise scales.
**Supplementary Figure 7**	The denoising benchmark of DeepSeMi and other methods on Gaussian-noise corrupted Moving MNIST datasets over different noise scales.
**Supplementary Figure 8**	Denoising benchmark of DeepSeMi and other methods on Gaussian-noise corrupted Moving MNIST datasets over different noise scales, part II.
**Supplementary Figure 9**	The denoising benchmark of DeepSeMi and other methods on Gaussian-noise corrupted Moving MNIST datasets over different content speeds.

**Supplementary Figure 10**	Comparison of DeepSeMi with DeepCAD and DeepInterpolation on Gaussian-noise corrupted Moving MNIST datasets over different content speeds.
**Supplementary Figure 11**	DeepSeMi corrects motion artifacts that defile DeepCAD.
**Supplementary Figure 12**	The denoising benchmark of DeepSeMi and other methods on Poissionian-noise corrupted Moving MNIST datasets.
**Supplementary Figure 13**	Evaluating denoising performance of DeepSeMi on mixed Gaussian and Poisson-noise corrupted scales.
**Supplementary Figure 14**	Characteristics of DeepSeMi in preserving spatial resolution and contrast during denoising.
**Supplementary Figure 15**	Evaluation of generalization ability of DeepSeMi on simulated datasets.
**Supplementary Figure 16**	Mitochondrial membrane-trained DeepSeMi effectively works on mitochondrial matrix and plasma membrane imaging.
**Supplementary Figure 17**	Corroborating the efficacy of mitochondrial membrane-trained DeepSeMi on mitochondrial matrix and cell membrane imaging through simultaneous low- and high-SNR confocal imaging system.
**Supplementary Figure 18**	Denoising of Tom20-GFP-mCherry-labeled mitochondria via DeepSeMi.

**Supplementary Figure 19**	DeepSeMi effectively enhances SNR of triple-color labeled multi organelles.
**Supplementary Figure 20**	Simultaneous low- and high-SNR confocal imaging system.
**Supplementary Figure 21**	Characteristics of the simultaneous low- and high-SNR confocal imaging system.
**Supplementary Figure 22**	Evaluate DeepSeMi on experimental high- and low-SNR confocal fluorescent recordings.
**Supplementary Figure 23**	Benchmarking DeepSeMi on experimental high- and low-SNR confocal recordings.
**Supplementary Figure 24**	Benchmarking DeepSeMi on experimental high- and low-SNR confocal recordings, part II.
**Supplementary Figure 25**	Denoising performance benchmark of DeepSeMi and other methods on experimental data procured through simultaneous high- and low-SNR confocal system.
**Supplementary Figure 26**	Assessment of the ability in maintaining intensity linearity through simultaneous low- and high-SNR confocal imaging system.
**Supplementary Figure 27**	Evaluating denoising performance of DeepSeMi under different imaging speeds through simultaneous low- and high-SNR confocal imaging system.

**Supplementary Figure 28**	Evaluation of photobleaching of mitochondria under different laser dosages.
**Supplementary Figure 29**	Laser power calibration on the Nikon A1 confocal microscopy.
**Supplementary Figure 30**	DeepSeMi helps automated segmentation and skeletonization of mitochondria under low power dosage.
**Supplementary Figure 31**	DeepSeMi enables high-SNR imaging of tri-color labeled L929 cells in low light.
**Supplementary Figure 32**	15-fold increment of photon budgets by DeepSeMi.
**Supplementary Figure 33**	DeepSeMi significantly enhances organelle imaging results with the dye dilution.
**Supplementary Figure 34**	DeepSeMi strongly reduced phototoxicity on imaging FM464 labeled cells.
**Supplementary Figure 35**	Significant photobleaching brought by dual-color confocal imaging.
**Supplementary Figure 36**	Statistics of mitochondrial segmentation and skeletonization under different illumination powers with and without DeepSeMi enhancement.
**Supplementary Figure 37**	Automated analysis of recorded mitochondria with DeepSeMi enhancement.

**Supplementary Figure 38**	Cellular segmentation benchmark of DeepSeMi and other denoising methods enhanced experimental data produced by simultaneous high- and low-SNR confocal system.
**Supplementary Figure 39**	Evaluating of DeepSeMi enhanced segmentation performance on mitochondria under different imaging speeds with simultaneous low- and high-SNR confocal imaging system.
**Supplementary Figure 40**	DeepSeMi-enhanced imaging results of L929 cells treated with Lat-A.
**Supplementary Figure 41**	DeepSeMi unveiled migrating cells interacting with a migrasome, producing migrasomes, and expelling mitochondria in low light dosage.
**Supplementary Figure 42**	Evaluation of phototoxicity in imaging *Dictyostelium* cells.
**Supplementary Figure 43**	Evaluation of phototoxicity in imaging *Dictyostelium* cells with a bright-field microscope imaging.
**Supplementary Figure 44**	DeepSeMi enables high-SNR imaging of contractile vacuole generation in photosensitive *Dictyostelium* cells.
**Supplementary Figure 45**	Time-lapse imaging of photo-sensitive Dictyostelium cells with simultaneous low- and high-SNR confocal imaging system.

**Supplementary Figure 46**	DeepSeMi enhanced cellular observation in scattering *C. elegans* in vivo.
**Supplementary Figure 47**	DeepSeMi enhances observation of zebrafish larvae in a low light dosage.
**Supplementary Figure 48**	DeepSeMi effectively recovers functional data on open-sourced two-photon Neurofinder datasets.
**Supplementary Figure 49**	Evaluation of DeepSeMi on hybrid high and low-SNR functional imaging.
**Supplementary Figure 50**	Evaluation of DeepSeMi on hybrid high and low-SNR dendritic imaging.

Video captions

**Supplementary Video 1**	Evaluation and segmentation of DeepSeMi enhancement over a triple-color labeled L929 cell in low light. First 18 seconds: comparison of the raw noisy captured video (top) and the DeepSeMi-enhanced video (bottom) of a triple-color labeled L929 in a commercial confocal microscope, with a panel indicating intensity profiles along the green line attached in the bottom right corner.18 to 37 seconds: comparison of raw (top) and DeepSeMi-enhanced zoom-in video outlined by the white box. 38 seconds to the end: comparison of mitochondrial segmentation and keypoint detection of raw (top) and DeepSeMi-enhanced (bottom) video (Methods), where red points for junction points, yellow points for endpoints, green lines for mitochondrial skeletons, and gray area for mitochondrial segments.
**Supplementary Video 2**	Evaluation of photobleaching of mitochondria under different laser dosages. Mitochondria in L929 cells were captured by a commercial confocal microscopy with five different excitation laser intensity (0.5% 14.6 μW, 1% 23.1 μW,2% 45.3 μW, 4% 80.4 μW, 8% 152.3 μW). The first row presented the raw (top) and DeepSeMi-enhanced (bottom) mitochondria movie. The second row presented corresponding photobleaching curves of each power dosage (Methods).
**Supplementary Video 3**	Evaluation of DeepSeMi enhancement in a quadruple-color labeled L929 cell in low light over 13,000 frames.

	The L929 cell was imaged in a commercial confocal microscope for a half-hour long session and presented in the left, and intensity profiles along the green and yellow lines were dynamically presented on the right, where the first and third rows for raw and the second and fourth rows for DeepSeMi. The first 12 seconds: raw noisy captured video of a L929 cell. 12 seconds to the end: DeepSeMi enhanced video of a L929 cell. Frame numbers and time stamps were annotated on the right bottom.
**Supplementary Video 4**	Evaluation of DeepSeMi enhancement in observation of cell migrations in low light over 12 hours. Two L929 cells were imaged in a commercial confocal microscope over 80,000 frames. The first 40 seconds: raw (left) and DeepSeMi-enhanced (right) videos representing migrations of two L929 cells in a global view. 40 seconds to the end: raw (left) and DeepSeMi-enhanced (right) video representing migrations of two L929 cells in a zoom-in view, where generation of migrasomes was clearly presented. Frame numbers and time stamps were annotated on the right bottom.
**Supplementary Video 5**	Evaluation of DeepSeMi enhancement in observation of retractosomes generation. Two L929 cells were imaged in a commercial confocal microscope for over 24,000 seconds. Raw (left) and DeepSeMi-enhanced (right) videos representing generation of retractosomes were

	presented. Frame numbers and time stamps were annotated on the right bottom.
**Supplementary Video 6**	Evaluation of DeepSeMi enhancement in observation of intercell interactions in low light over 2 hours. Two L929 cells were imaged in a commercial confocal microscope. The first 40 seconds: raw (top) and DeepSeMi-enhanced (bottom) video representing the generation of migrasomes on retraction fibers in a global view. 40 seconds to 1 minute 5 seconds: raw (top) and DeepSeMi-enhanced (bottom) videos representing the generation of migrasomes on retraction fibers in a zoom-in view. 1 minute 5 seconds to 1 minute 20 seconds: raw (top) and DeepSeMi-enhanced (bottom) video representing interactions between a cell and a migrasome. 1 minute 20 seconds to the end: raw (top) and DeepSeMi-enhanced (bottom) videos representing a long-distance movement of mitochondria. Frame numbers and time stamps were annotated on the right bottom.
**Supplementary Video 7**	Evaluation of DeepSeMi enhancement in observation of *Dictyostelium* cells in low light. Three *Dictyostelium* cells were imaged in a commercial confocal microscope over 1800 seconds and presented in 0~192 seconds, 0~276 seconds, and 7~1308 seconds, respectively.

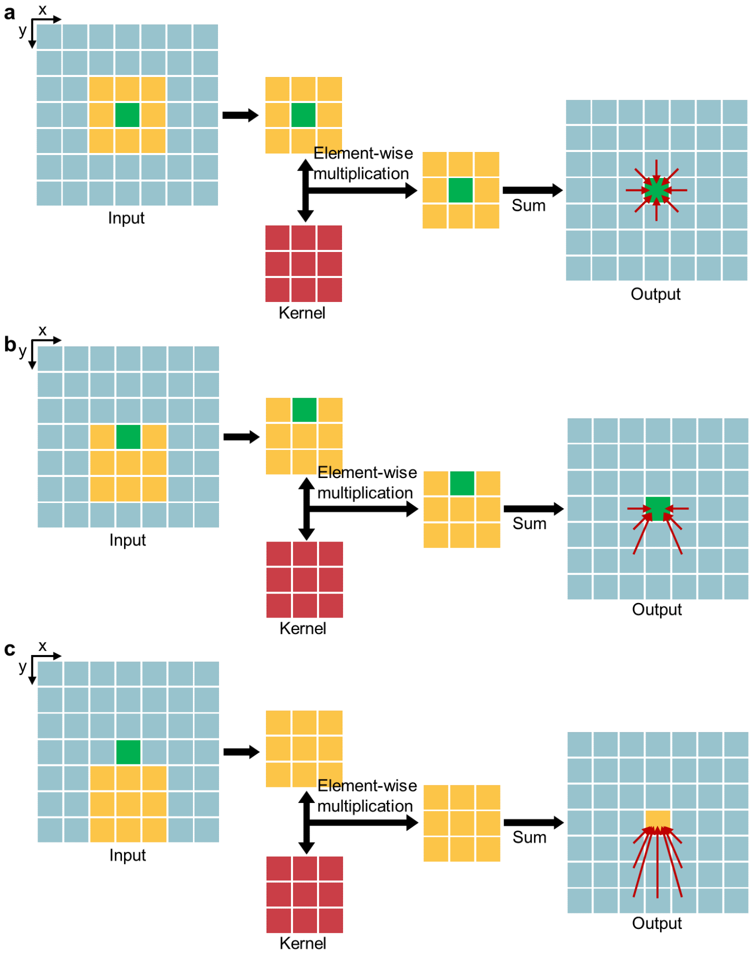

Supplementary Figure 1

Comparison of the proposed eccentric convolutional filter with the traditional 2D convolution. **a**, Processing of the traditional convolutional filter. The convolution procedure takes place around the target pixel (green) with a kernel size of 3x3 (red), and the receptive field is marked by yellow pixels and the green pixel. **b**, Processing of the eccentric convolutional filter. The symbols are the same as **a**. Note the receptive field is biased off the target pixel but does not entirely miss the target pixel. Due to the concatenated fashion of the convolutional neural network, the receptive field of following convolutional layers is also limited. **c**, Processing of the eccentric blind-spot convolutional filter. The symbols are the same as **a**. Note the receptive field is entirely off the target pixel.

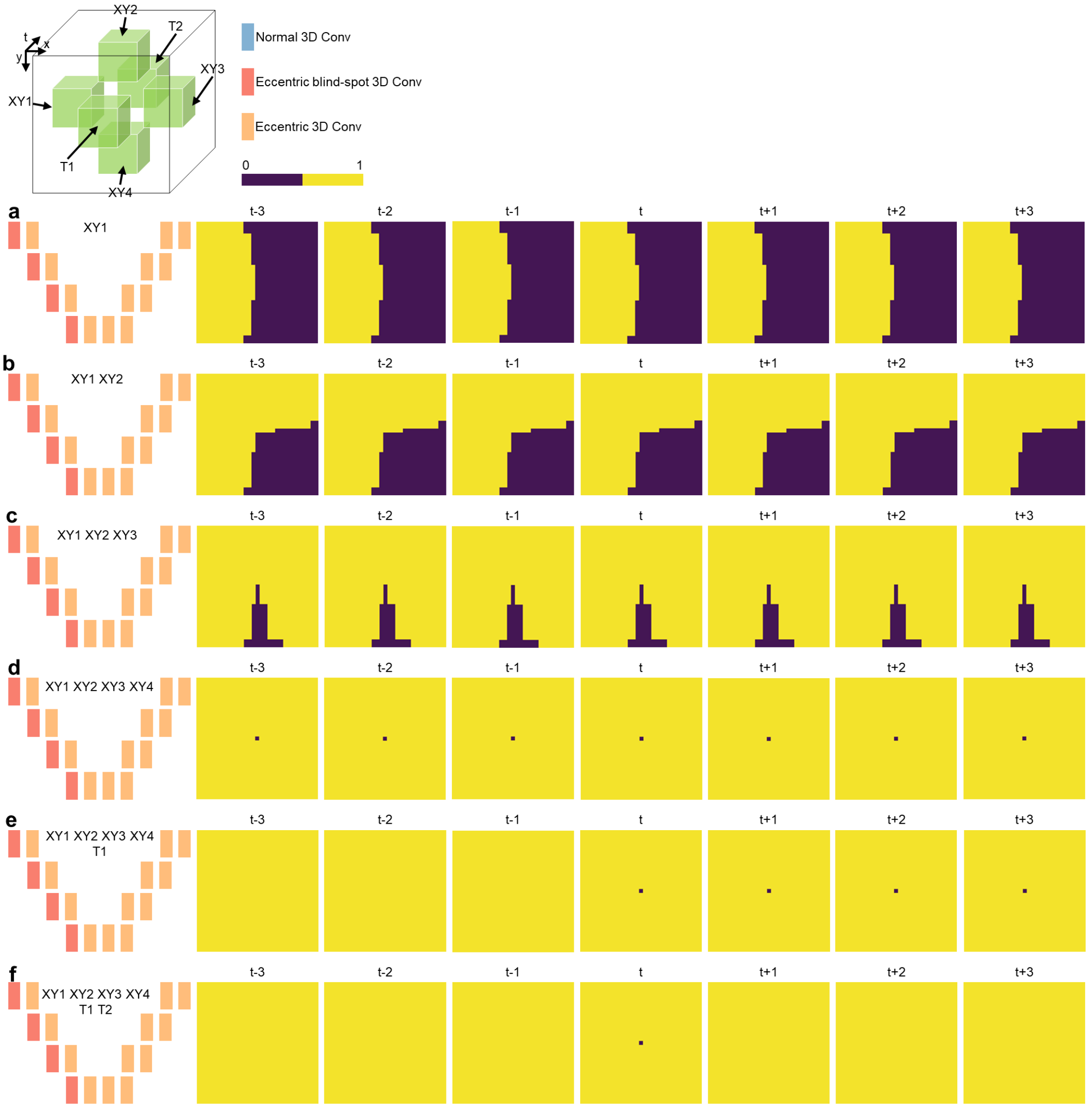

Supplementary Figure 2

Receptive field visualization of networks with various branches. As the meaning of the receptive field is how many pixels around the target noisy pixel participate in the inference of the denoising pixel, we binarized the receptive field to better display the receptive field range. We term the four spatial branches as XY1, XY2, XY3, and XY4, which represent the surrounding four pixels in the same frame, and we term the two temporal branches as T1 and T2, which represents the pixels at the same position but from the previous and the next frame. Three different convolutional layers are differently colored. **a**, Receptive field of the network with only one spatial branch (XY1). Left: the

simplified diagram of neural network structure. Right: the receptive field in three dimensions (XYT). The corresponding time stamp is marked at the top of each frame. **b**, Receptive field of the network with two spatial branches (XY1, XY2). **c**, Receptive field of the network with three spatial branches (XY1, XY2, XY3). **d**, Receptive field of the network with all four spatial branches (XY1, XY2, XY3, XY4). **e**, Receptive field of the network with four spatial branches and one temporal branch (XY1, XY2, XY3, XY4, T1). **f**, Receptive field of the network with four spatial branches and two temporal branches (XY1, XY2, XY3, XY4, T1, T2), which we term as DeepSeMi.

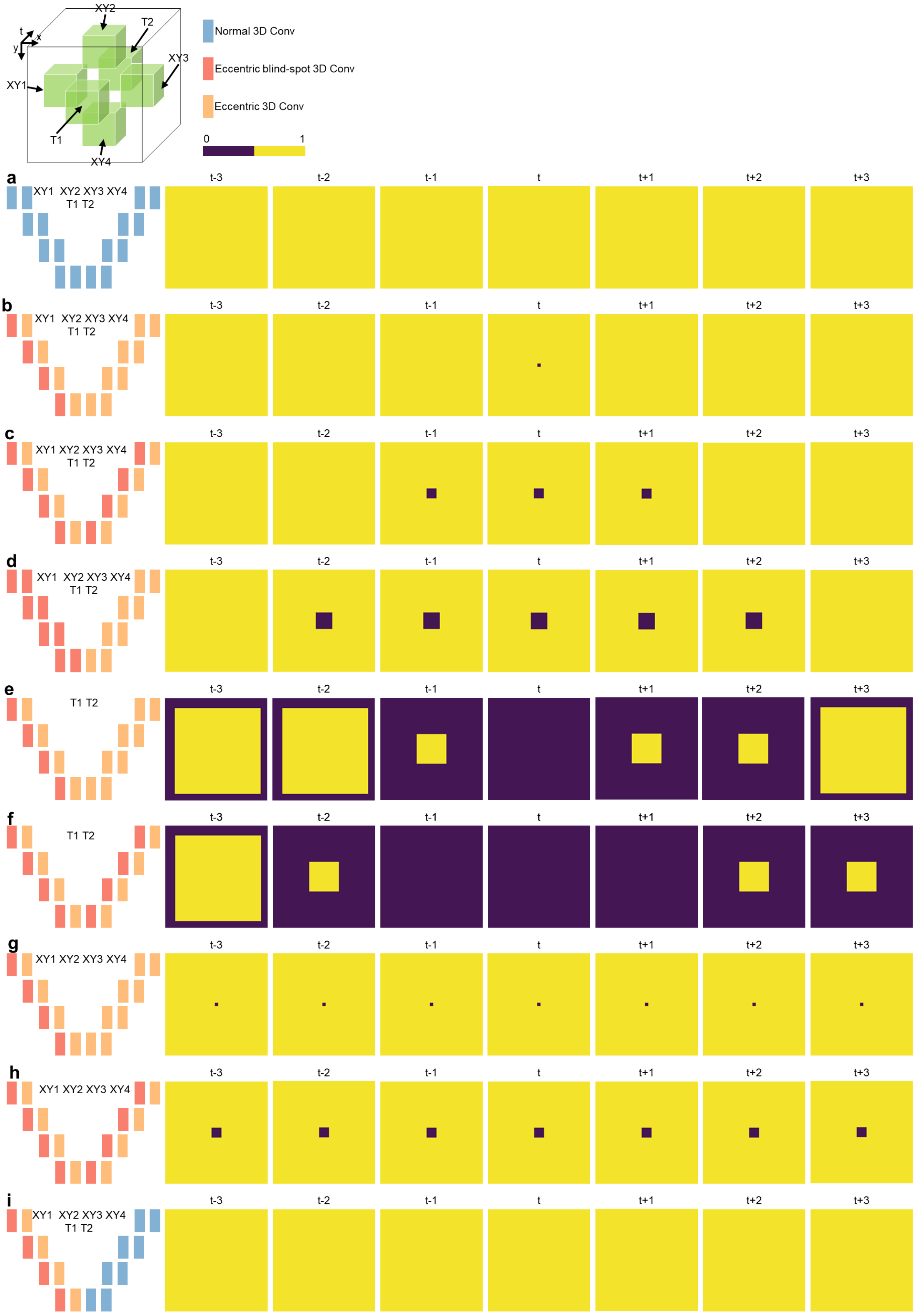

50 **Supplementary Figure 3**

Receptive field visualization of networks with various convolutional filters and various branches. **a**, Receptive field of a neural network composed of normal 3D convolutions (N3Dconv, i.e. not eccentric or eccentric blind-spot convolutions) and 6 branches (4 spatial branches: XY1, XY2, XY3, XY4; 2 temporal branches: T1, T2). Left: the simplified diagram of the network structure where each block represents a convolutional layer. Right: the receptive field in three dimensions. The corresponding time stamp is marked in the top of the image. **b**, Receptive field of the network with 6 branches composed by eccentric blind-spot 3D convolution (EB3Dconv) and eccentric 3D convolution (E3Dconv). EB3Dconv only appears in the encoding path. As a result, $1\times1\times1$ blind spot is achieved in the receptive field. **c**, Receptive field of the network with 6 branches composed by EB3Dconv and E3Dconv. EB3Dconv appears both in the encoding and decoding paths. As a result, $3\times3\times3$ blind spot is achieved in the receptive field. **d**, Receptive field of the network with 6 branches composed by EB3Dconv and E3Dconv. Encoding paths are fully made by EB3Dconv. As a result, $5\times5\times5$ blind spot is achieved in the receptive field. **e**, Receptive field of the network with 2 temporal branches composed by EB3Dconv and E3Dconv. EB3Dconv appears only in the encoding paths. As a result, $n\times n\times1$ blind spot is achieved in the receptive field, where n is the patch size. **f**, Receptive field of the network with 2 temporal branches composed by EB3Dconv and E3Dconv. EB3Dconv appears both in the encoding and decoding paths. As a result, $n\times n\times3$ blind spot is achieved in the receptive field, where n is the patch size. **g**, Receptive field of the network with 4 spatial branches composed by EB3Dconv and E3Dconv. EB3Dconv appears only in the encoding path. As a result, $1\times1\times t$ blind spot is achieved in the receptive field, where t is the length of the sequence. **h**. Receptive field of the network with 4 spatial branches composed by EB3Dconv and E3Dconv. EB3Dconv appears both in the encoding and decoding paths. As a result, $3\times3\times t$ blind spot is achieved in the receptive field, where t is the length of the sequence. **i**. Receptive field of the network with 6 branches composed by EB3Dconv, E3Dconv, and normal 3D

convolutions. EB3Dconv and E3Dconv only appear in the encoding path, and normal 3D convolutions only appears in the decoding path. As a result, no blind spot is achieved in the receptive field.

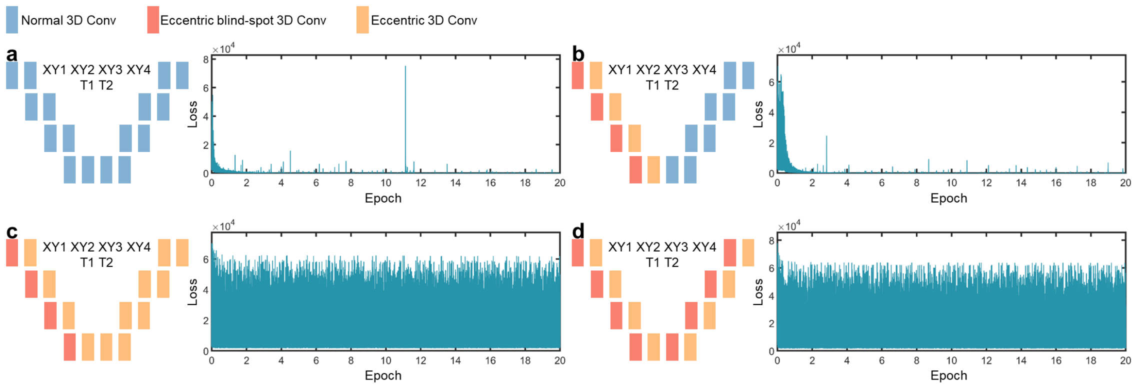

Supplementary Figure 4

Analysis of network characteristics with varying blind spots by evaluating their inability to learn a trivial identity map. The training process was implemented using unlabeled, noisy data, with each image serving as its own reference. **a**, Loss fluctuation of a network composed of normal 3D convolutions (N3Dconv, i.e. not eccentric or eccentric blind-spot convolutions) and 6 branches (4 spatial branches: XY1, XY2, XY3, XY4; 2 temporal branches: T1, T2). The receptive field of such a network is shown in Supplementary Fig. 3**a**. Left: the simplified diagram of neural network structure, where symbols and notions are the same as Supplementary Figs. 2 and 3. Right: The training loss during a 20-epoch training. **b**, Loss fluctuation of a network with 6 branches composed by eccentric blind-spot 3D convolution (EB3Dconv), eccentric 3D convolution (E3Dconv), and normal 3D convolutions. EB3Dconv and E3Dconv only appear in the encoding path, and normal 3D convolutions only appear in the decoding path. The receptive field of such a network is shown in Supplementary Fig. 3**i**. **c**, Loss fluctuation of a network with 6 branches composed by EB3Dconv and E3Dconv. EB3Dconv only appears in the encoding path. The receptive field of such a network is shown in Supplementary Fig. 3**b**. **d**, Loss fluctuation of a network with 6 branches composed by EB3Dconv and E3Dconv. EB3Dconv appears both in the encoding and decoding paths. The receptive field of such a network is shown in Supplementary Fig. 3**c**.

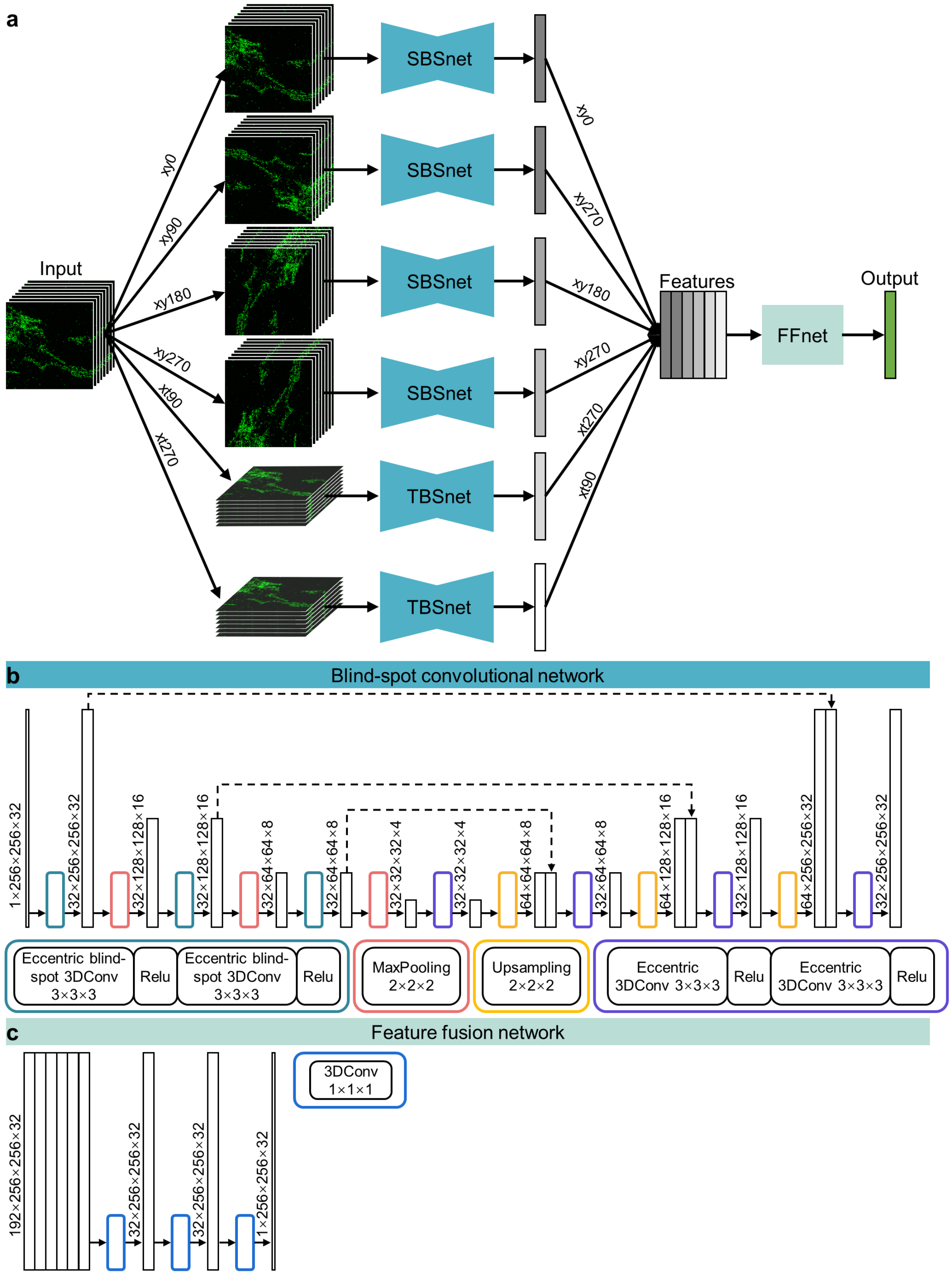

Supplementary Figure 5

The network structure of DeepSeMi. **a**, Six branches of hybrid blind-spot 3D neural network which has different preferential directions of reception field compose the DeepSeMi. Among the six branches, four networks are spatial blind-spot 3D neural

networks (SBSnets) and two networks are temporal blind-spot 3D neural networks (TBSnets). The four SBSnets and the two TBSnets share the same parameters, respectively. Output features from the six branches are concatenated and input to a feature fusion network (FFnet) for the final output. **b**, Detailed structure of the blind-spot 3D neural network in each branch. **c**, Detailed structure of the feature fusion network. Feature fusion network takes advantage of 1×1×1 3D convolutions to merge features from each branch.

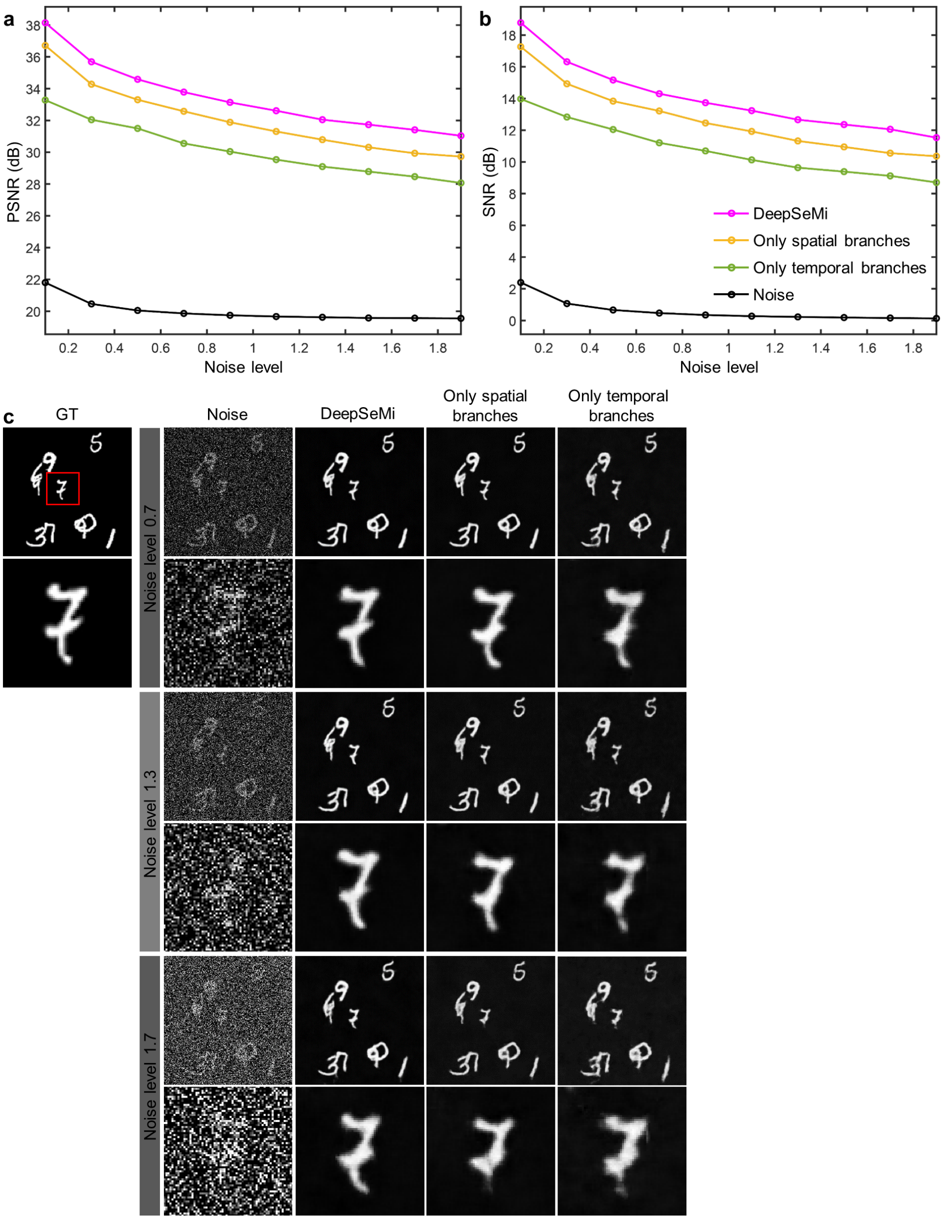

Supplementary Figure 6

Ablation study of DeepSeMi in Moving MNIST datasets over different noise scales. **a-b,** Peak-signal-to-noise-ratio (PSNR) and signal-to-noise-ratio (SNR) comparisons of DeepSeMi, DeepSeMi with only spatial branches, DeepSeMi with only temporal branches, raw images at different noise levels. The motion speed is set as 5, which means the handwritten digits in the next frame are shifted by 5 pixels relative to the previous frame. **c**, Exemplary denoising results of DeepSeMi and other ablated versions of DeepSeMi over three noise levels. The first row on each noise level represents the full

field and the second row represents the zoom-in area marked by the red box (in this case, the number “7”).

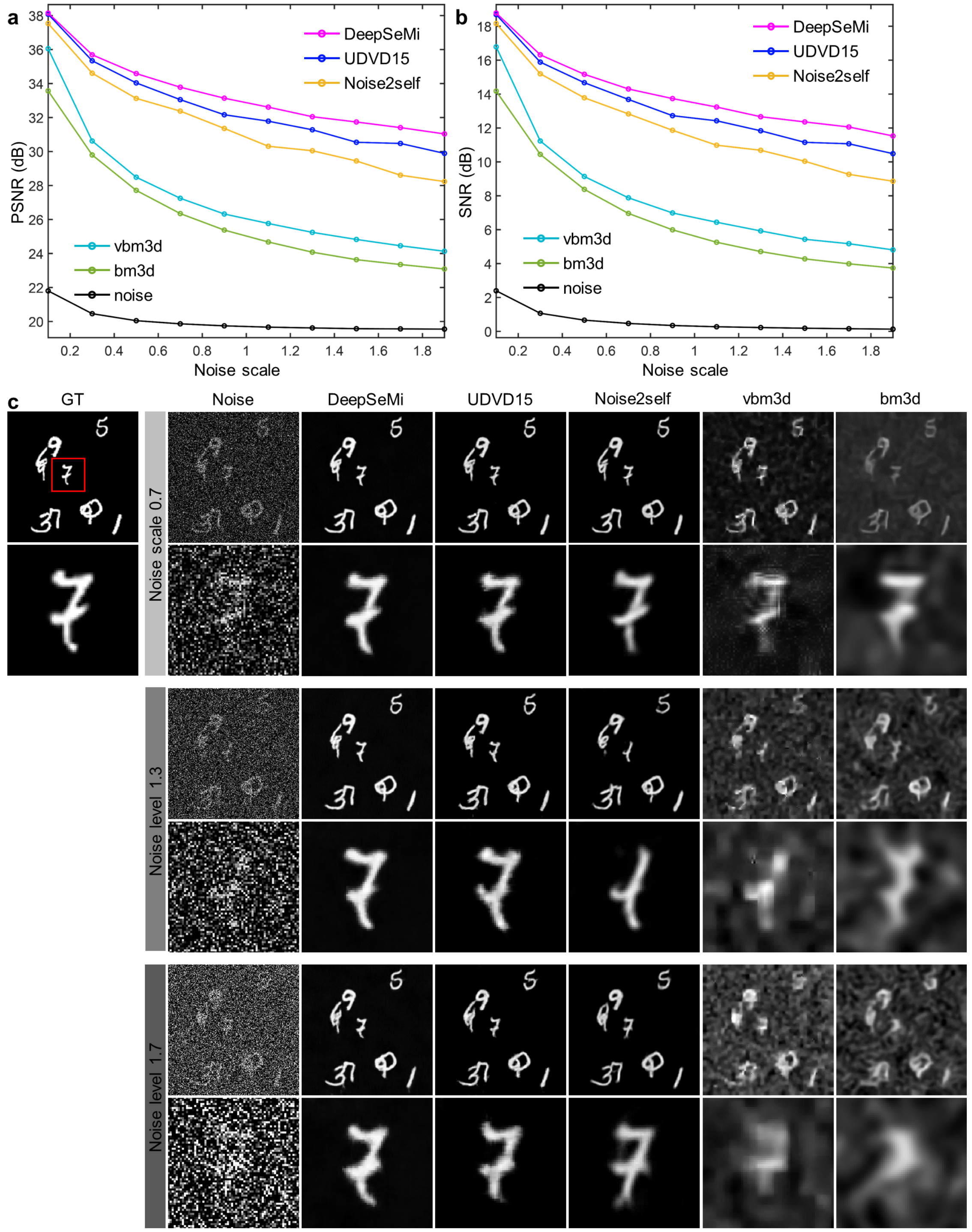

Supplementary Figure 7

Denoising benchmark of DeepSeMi and other methods on Gaussian-noise corrupted Moving MNIST datasets over different noise scales, part I. Comparative methods encompass UDVD15 [1], Noise2Self [2], VBM3D [3], and BM3D [4]. See Supplementary Fig. 8 for additional comparison with Noise2same [5], Noise2void [6],

Probabilistic Noise2Void [7], and DivNoising [8]. **a-b,** PSNR and SNR comparisons of DeepSeMi, UDVD15 [1], Noise2Self [2], VBM3D [3], and BM3D [9] at different noise levels. The motion speed is set as 5, which means the handwritten digits in the next frame are shifted by 5 pixels relative to the previous frame. **c**, Exemplary denoising results of DeepSeMi and other methods over three noise levels. The first row on each noise level represents the full field and the second row represents the zoom-in area marked by the red box (in this case, the number “7”).

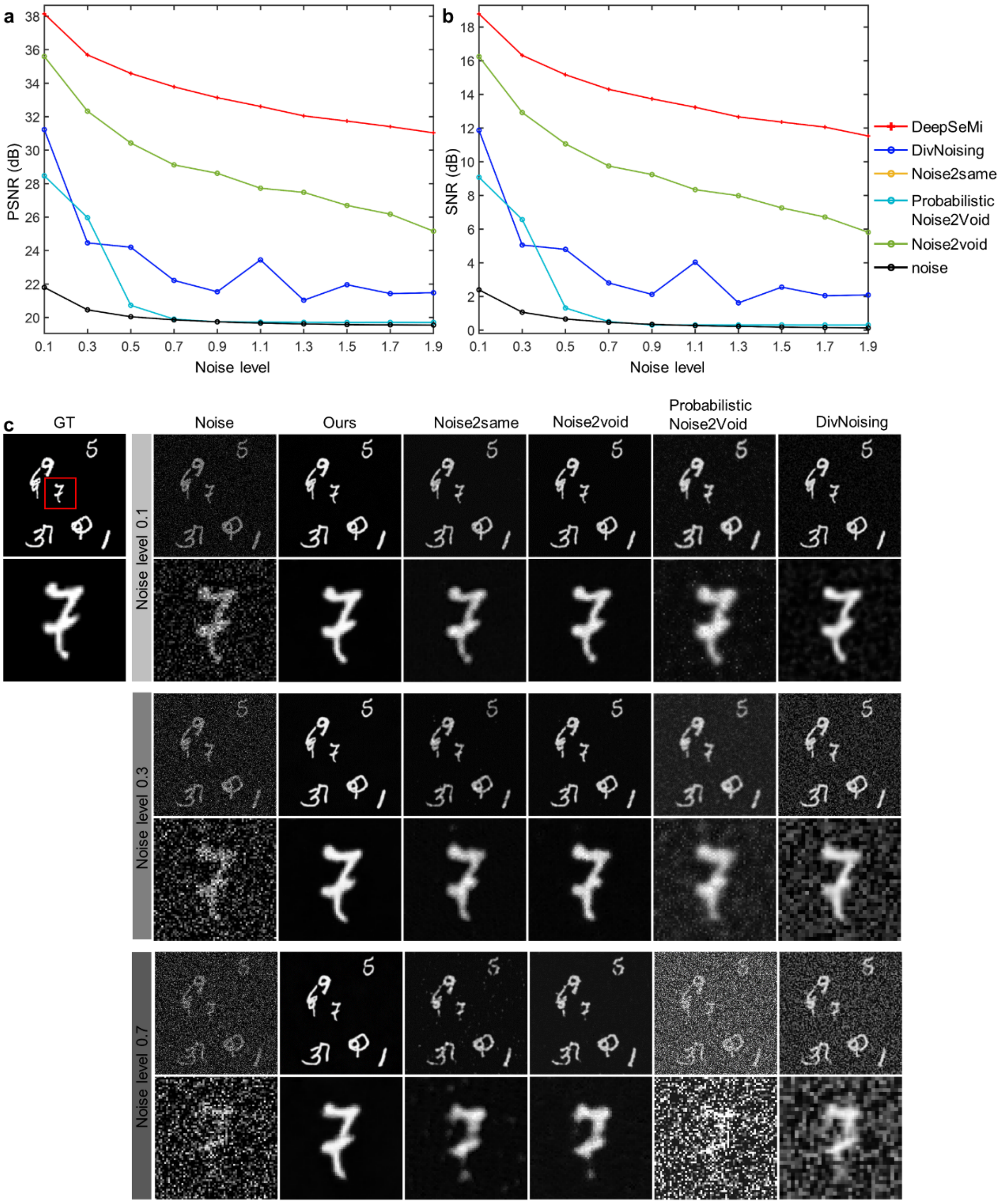

Supplementary Figure 8

Denoising benchmark of DeepSeMi and other methods on Gaussian-noise corrupted Moving MNIST datasets over different noise scales, part II. Comparative methods encompass Noise2same [5], Noise2void [6], Probabilistic Noise2Void [7], and DivNoising [8]. See Supplementary Fig. 7 for additional comparison with VBM3D [3], BM3D [4], Noise2Self (N2S) [2], and UDVD [1]. **a-b,** PSNR and SNR comparisons of Noise2same, Noise2void, Probabilistic Noise2Void, and DivNoising at different noise

levels. The motion speed is set as 5, which means the handwritten digits in the next frame are shifted by 5 pixels relative to the previous frame. **c**, Exemplary denoising results of DeepSeMi and other methods over three noise levels. The first row on each noise level represents the full field and the second row represents the zoom-in area marked by the red box (in this case, the number “7”).

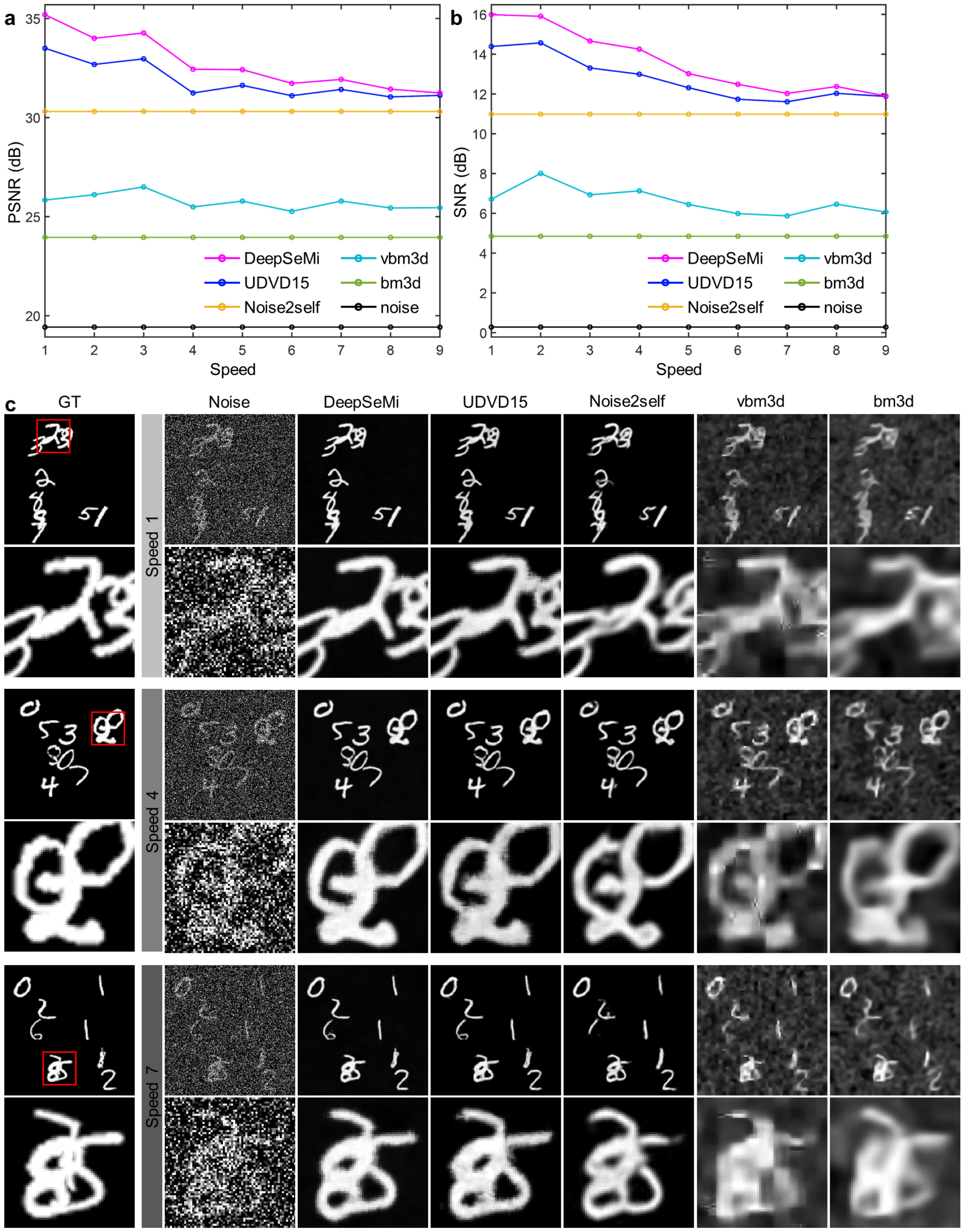

Supplementary Figure 9

Denoising benchmark of DeepSeMi and other methods on Gaussian-noise corrupted Moving MNIST datasets over different content speeds. a-b, PSNR and SNR comparisons of DeepSeMi, UDVD15 [1], Noise2Self [2], VBM3D [3], and BM3D [9] at

different content motion speeds. The motion speed N is defined as the relative shift step in pixel between adjacent frames of each handwritten digit. **c**, Exemplary denoising results of DeepSeMi and other methods over three motion speeds. The first row on each speed represents the full field and the second row represents the zoom-in area marked by the red box.

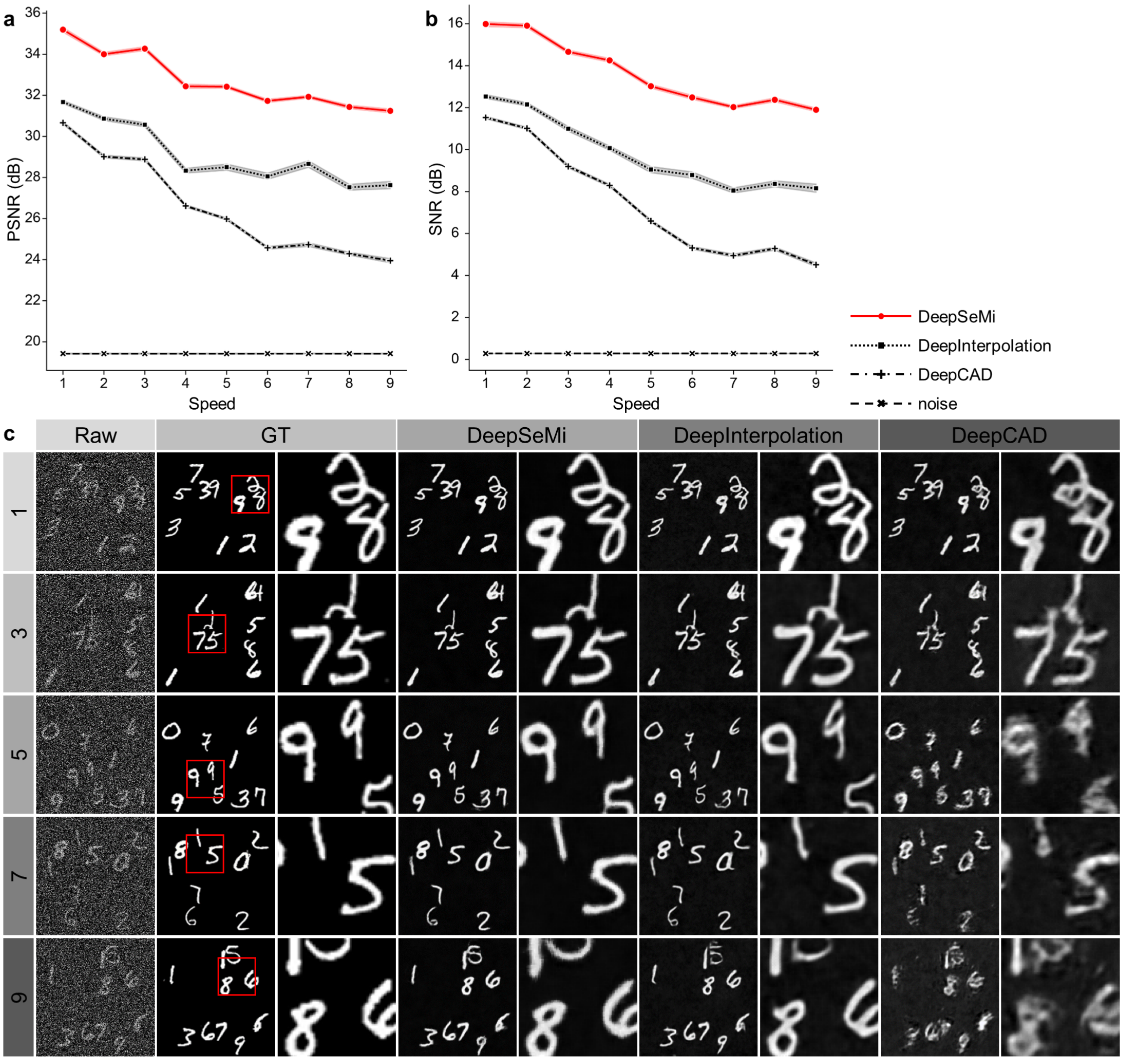

Supplementary Figure 10

Comparison of DeepSeMi with DeepCAD and DeepInterpolation on Gaussian-noise corrupted Moving MNIST datasets over different content speeds. **a-b**, PSNR and SNR comparisons of DeepSeMi, DeepCAD [10], and DeepInterpolation [11]. The motion speed N is defined as the relative shift step in pixel between adjacent frames of each handwritten digit. The noise level is set as 1.1. **c**, Exemplary denoising results of DeepSeMi and other methods over five motion speeds. The first column on each speed represents the full field and the second column represents the zoom-in area marked by the red box.

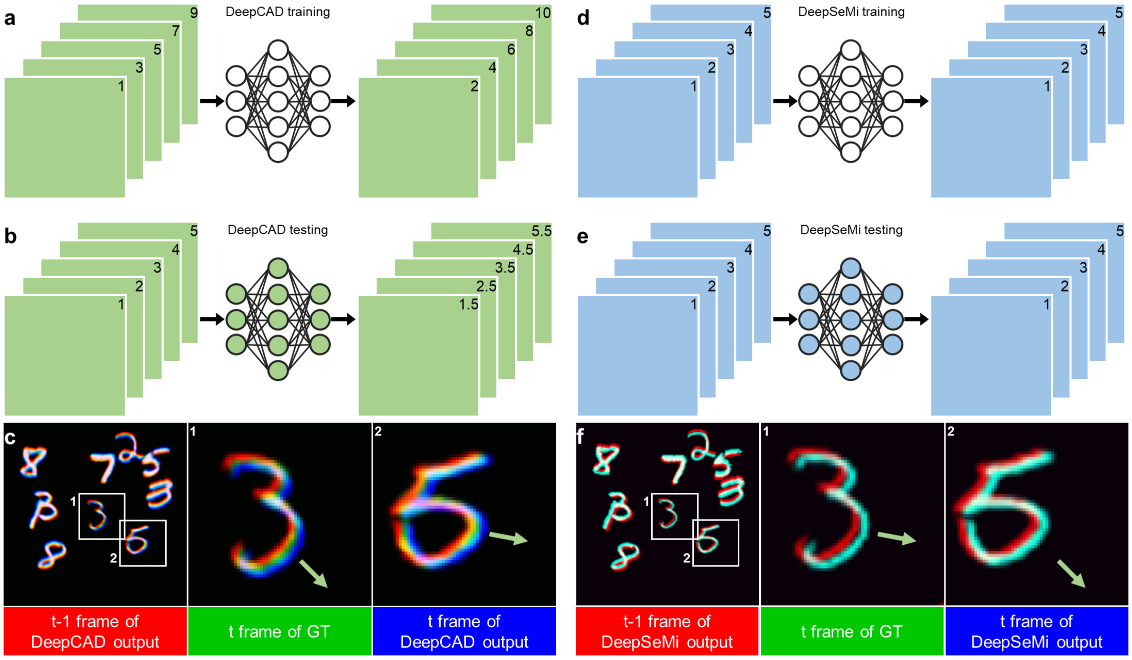

Supplementary Figure 11

DeepSeMi corrects motion artifacts that defile DeepCAD. a, Schematic diagram represents the DeepCAD training process with the frame number labeled. **b,** Schematic diagram represents the DeepCAD testing process. Note the inferred frames are the interpolations between captured adjacent frames that are not physically captured. **c,** Motion artifacts of DeepCAD on Moving MNIST datasets. A DeepCAD model was trained and tested on Moving MNIST datasets (left), and temporal-color-coded digits were presented in zoom-in panels (right). The motion directions are labeled by the arrows. The red channel represents the output of DeepCAD in frame *t-1*, the green channel represents the ground truth in frame *t*, and the blue channel represents the output of DeepCAD in frame *t*. Blue and green channels were obviously separated, indicating prediction bias in DeepCAD over moving contents. **d-f**, The same as **a-c** but from results by DeepSeMi. Blue and green channels were closely matched in **f**, indicating no motion artifacts generated by DeepSeMi.

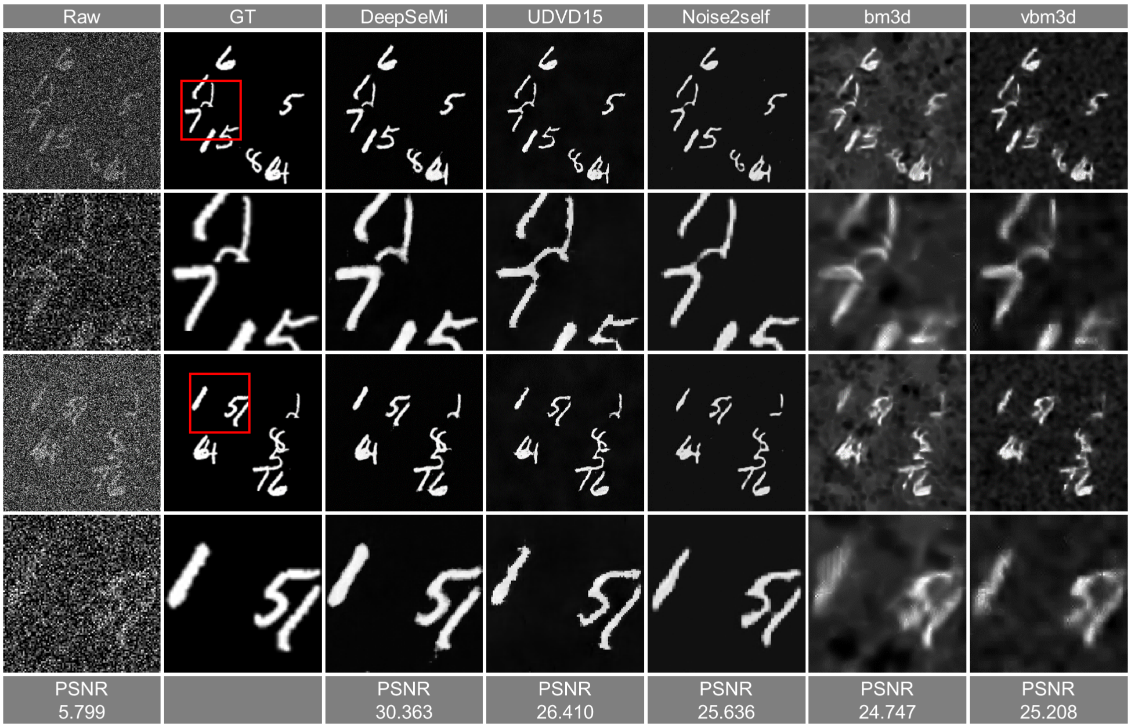

Supplementary Figure 12

Denoising benchmark of DeepSeMi and other methods on Poission-noise corrupted Moving MNIST datasets. We test the denoising performance of DeepSeMi, UDVD 15 [1], Noise2self [2], BM3D [9], and VBM3D [3]. The motion speed is set as 5. The second and fourth rows are magnified images of the red box in the first and third rows, respectively. Among these five algorithms, DeepSeMi has the best denoising performance with clean details.

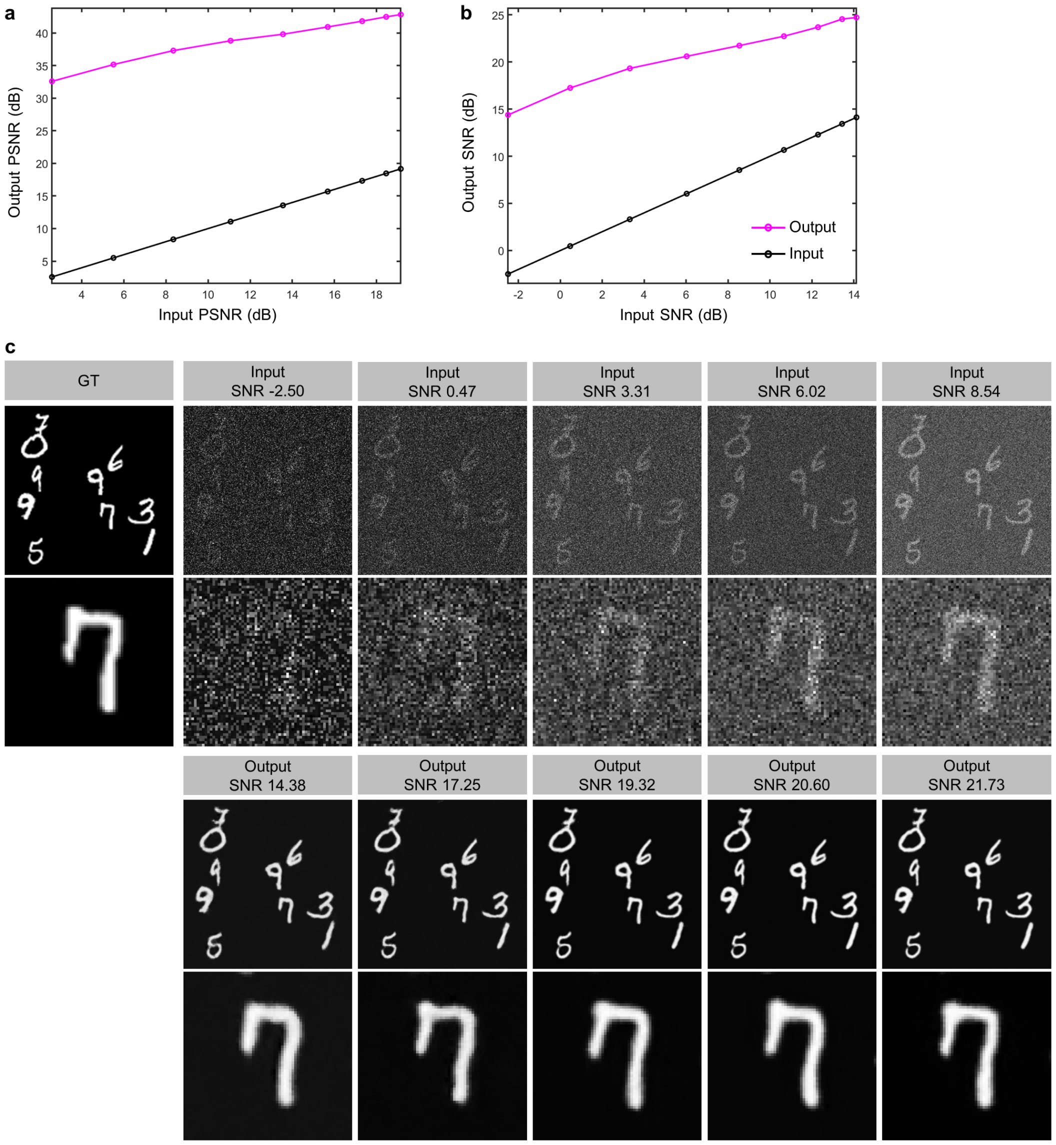

Supplementary Figure 13

Evaluating denoising performance of DeepSeMi on mixed Gaussian and Poisson-noise corrupted scales. a-b, PSNR and SNR of DeepSeMi enhanced images at different noise levels. The motion speed is set as 5, which means the handwritten digits in the next frame are shifted by 5 pixels relative to the previous frame. **c**, Exemplary denoising results of DeepSeMi over 5 noise levels. The first row on each noise level represents the full field and the second row represents the zoom-in area(in this case, the number “7”).

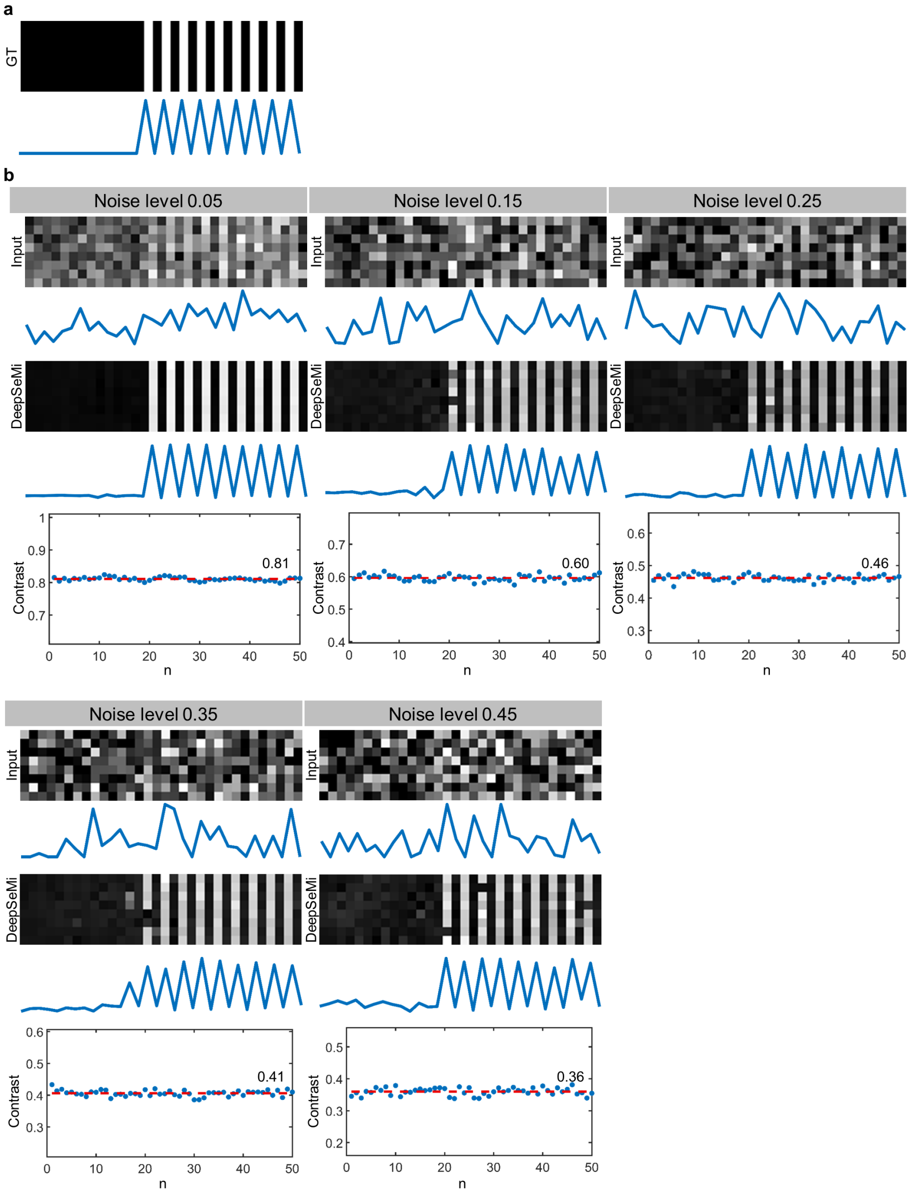

Supplementary Figure 14

Characteristics of DeepSeMi in preserving spatial resolution and contrast during denoising. a, At the top, the ground truth (GT) image displays parallel lines separated by equidistant spaces (1 pixel). At the bottom, corresponding intensity profiles along the horizontal axis across the ground truth are shown. **b**, Characteristics of DeepSeMi's ability in preserving spatial resolution and contrast under varying noise conditions. Five noise scales are examined, ranging from 0.05 to 0.45. For each noise scale, the first row reveals the noisy image with its corresponding intensity profile charted, the second row

shows the DeepSeMi denoised image with its corresponding intensity profile charted, and the third row exhibits the bar contrast across 50 sets of line pairs (blue dots) alongside their average (red dashed line).

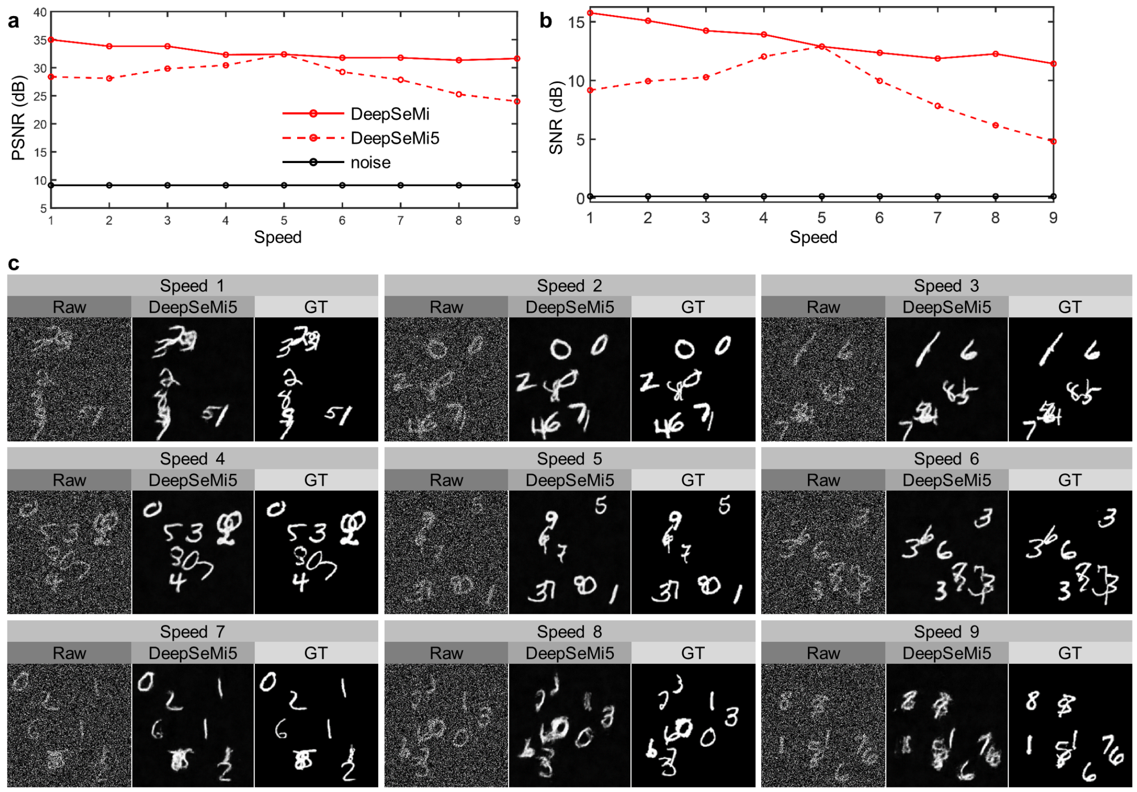

Supplementary Figure 15

Evaluation of generalization ability of DeepSeMi on simulated datasets. DeepSeMi is trained through the simulation data with a speed of 5 (the handwritten digit in the next frame is shifted by 5 pixels relative to the previous frame) and a noise level of 1.1, and is termed as DeepSeMi5. The trained DeepSeMi5 is used to denoise simulation data with different motion speeds in the following panels. **a-b**, The generalization ability of DeepSeMi5 over different content speeds but the same noise level measured by PSNR and SNR comparison. The red solid line represents results by DeepSeMi trained on datasets with current content speed and noise level. The red dashed line represents results by DeepSeMi5. The black solid line represents the characteristics of noise. **c**. Exemplary plots of denoising results of DeepSeMi5 over content speed 1 to 9.

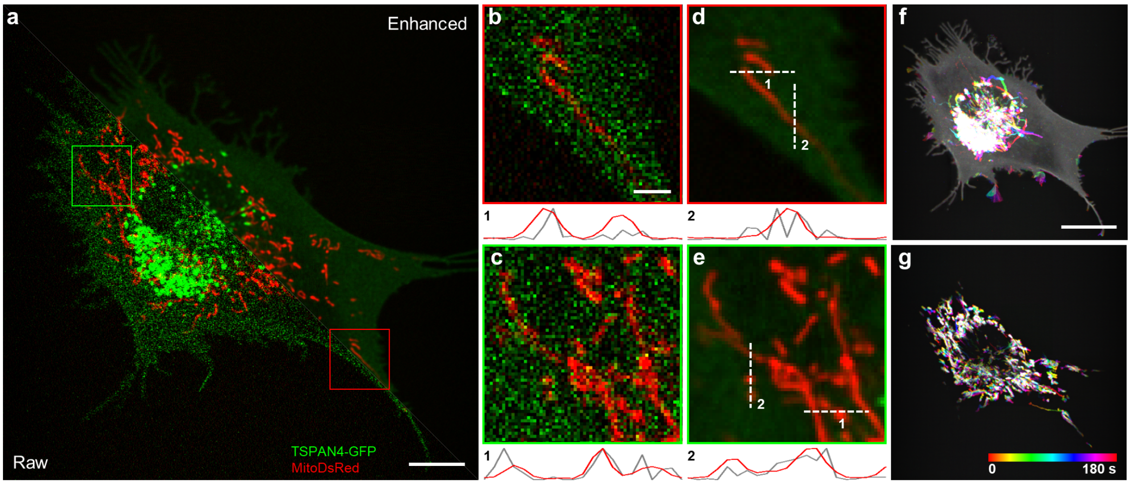

Supplementary Figure 16

Mitochondrial membrane-trained DeepSeMi effectively works on mitochondrial matrix and plasma membrane imaging. a, Raw (left) and DeepSeMi enhanced (right) images of simultaneously captured plasma membrane (green) and mitochondrial matrix (red). The dual color-labeled cells were captured by a commercial confocal imaging system. To test the generalization of the proposed method, we trained DeepSeMi on the green channel only (i.e. contains only the plasma membranes), but tested on both the green and the red channel (i.e. both the plasma membranes and the mitochondrial matrix). Tested images are exhibited in this figure. Scale bar, 10 µm. **b-e**, Zoom-in panels of the box-enclosed regions in **a**. Intensity profiles along dashed lines are plotted at the bottom. Scale bar, 2 µm. **f-g**, Temporal-color coded plasma membrane and mitochondrial dynamics, respectively. Scale bar, 20 µm.

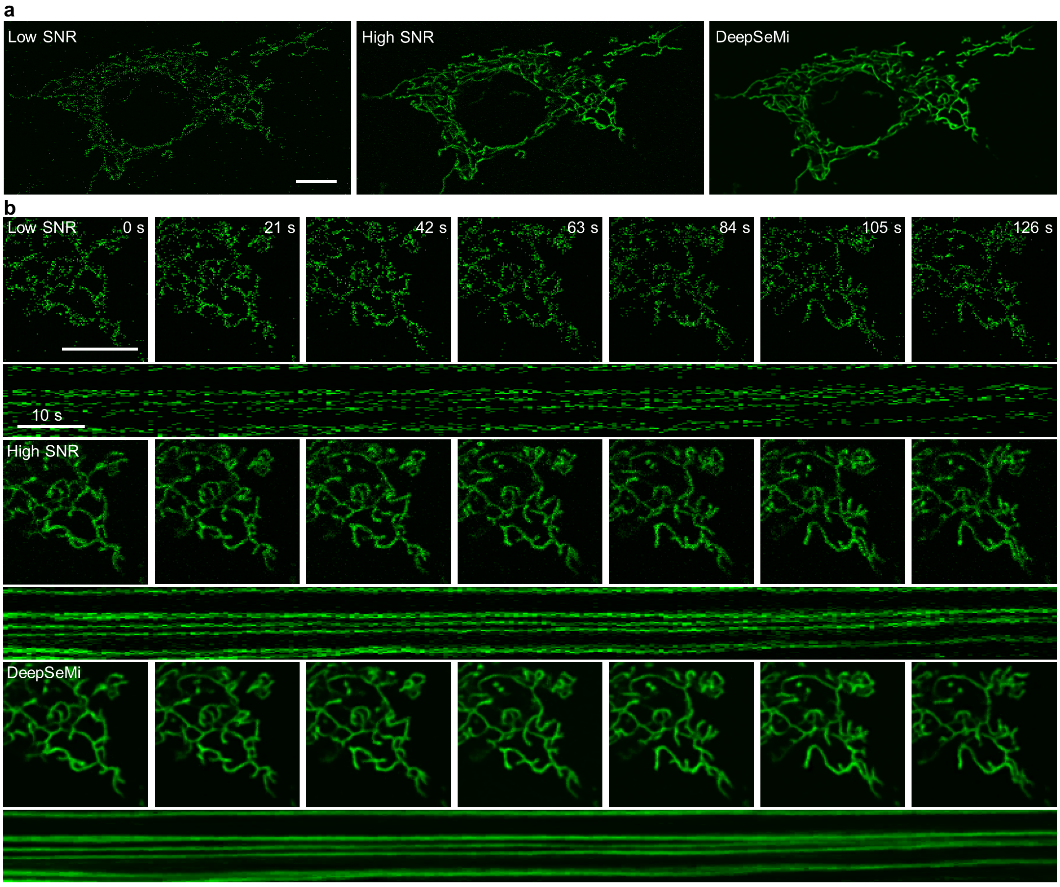

Supplementary Figure 17

Corroborating the efficacy of mitochondrial membrane-trained DeepSeMi on mitochondrial matrix and cell membrane imaging through simultaneous low- and high-SNR confocal imaging system. As in Supplementary Fig. 9, DeepSeMi was trained on experimental data from the mitochondrial membrane and subsequently applied to both the cell membrane and mitochondrial matrix data. **a**. Low-SNR (left), high-SNR (middle), and DeepSeMi enhanced (right) mitochondrial matrix imaging. Scale bar 10um. **b**. Time-lapse imaging of mitochondrial matrix with simultaneous low- and high-SNR confocal imaging system. For each timestamp, low-SNR frames, DeepSeMi recovered frames, and high-SNR frames were presented in different rows, respectively. Seven columns represented 7 time points as labeled in the top right corner. Scale bar 10um.

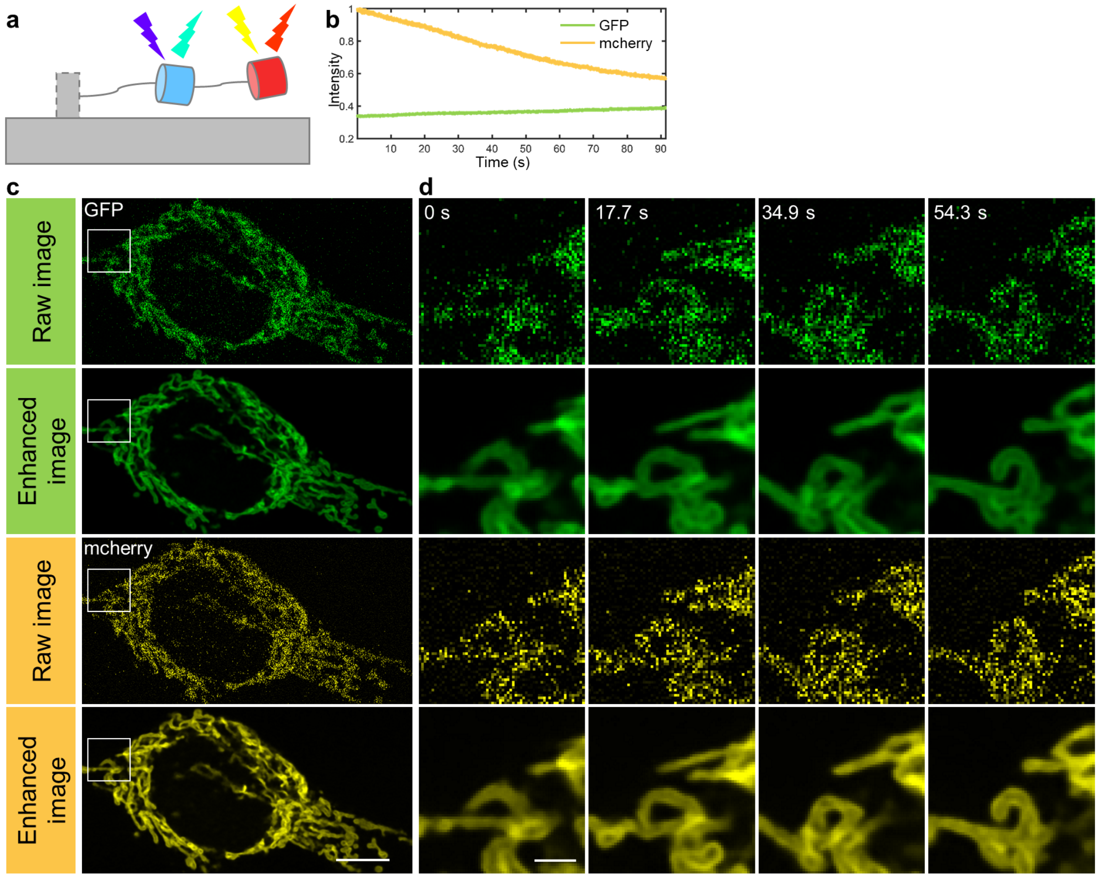

Supplementary Figure 18

Denoising of Tom20-GFP-mCherry-labeled mitochondria via DeepSeMi. **a**, Schematic diagram of Tom20-GFP-mCherry-labeled mitochondria which carries two fluorescence tag. **b**, Different photobleaching rates of GFP and mCherry in the co-labeled mitochondria. **c**, Raw and DeepSeMi denoised results labeled by GFP and mCherry. Scale bar, 10 µm. **d**, Zoom-in view of the white box in **c** at different time points. Scale bar, 2 µm.

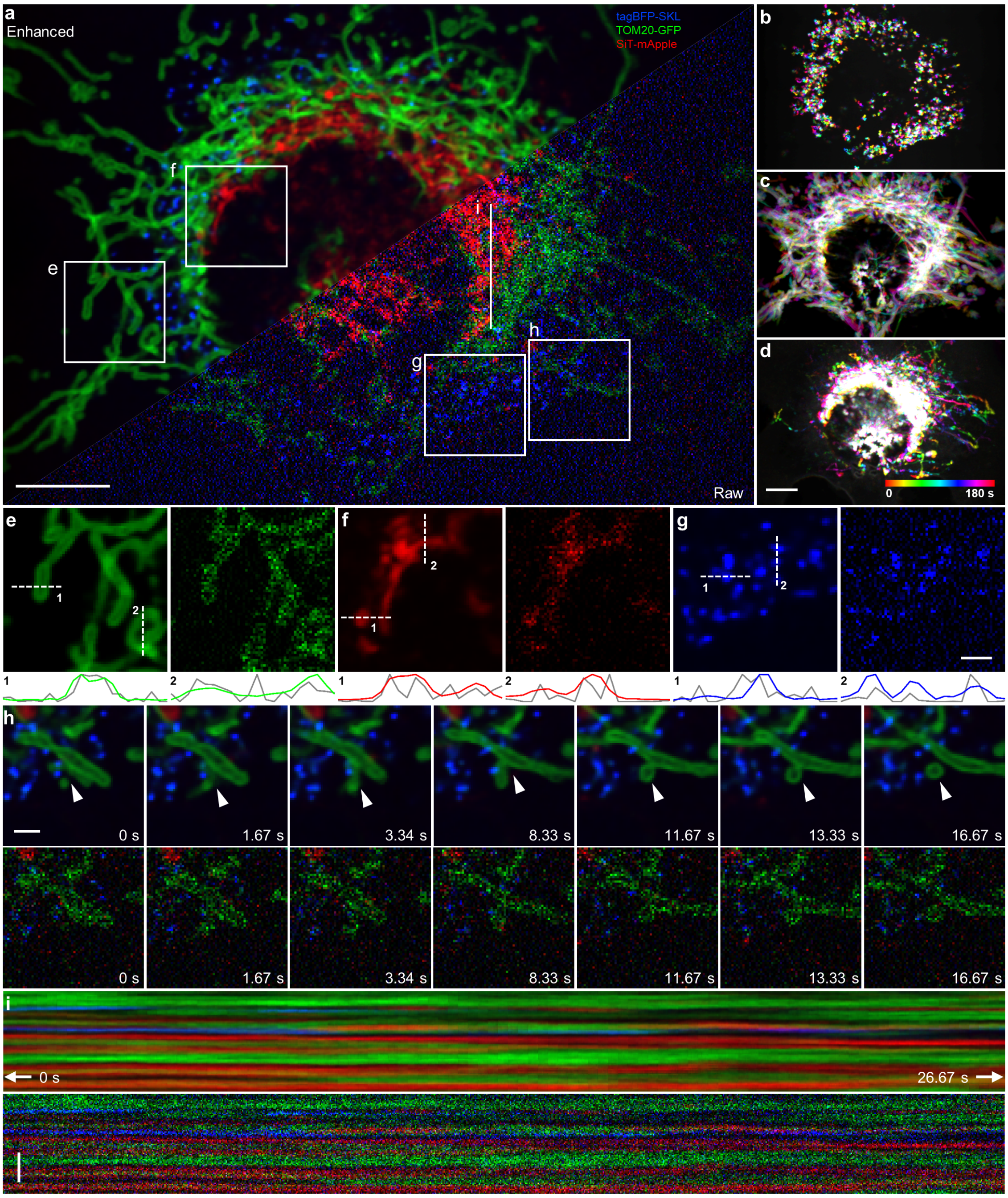

Supplementary Figure 19

DeepSeMi effectively enhances SNR of triple-color labeled multiple organelles. a, Raw (right) and DeepSeMi denoised (left) images of mitochondria (green), peroxisomes (blue), and Golgi (red) in an L929 cell in 1,800 frames per channel during 180 seconds. Scale bar, 10 μm. **b-d**, Temporal color coding of denoised mitochondria, peroxisomes, Golgi images to reflect organelle dynamics. Scale bar, 10 μm. **e-g**, Zoom-in panels of

white boxes marked in **a**. The membranous structures of mitochondria and the punctate structures of peroxisomes were clearly retrieved by DeepSeMi, and the structure profiles along the white dashed lines (colorful, bottom of the figure) are more reasonable compared to raw images (dark, bottom of the figure). Scale bar, 2 μm. **h**, Time-lapse presentation of a vesicle fission event by DeepSeMi (top) and raw (bottom), where a globular mitochondria split from another rod-shaped mitochondrion. Scale bar, 2 μm. **i**, Kymographs (y-t view) of raw images (bottom) and DeepSeMi enhanced images (top) along the white solid line in **a**. Scale bar, 4 μm.

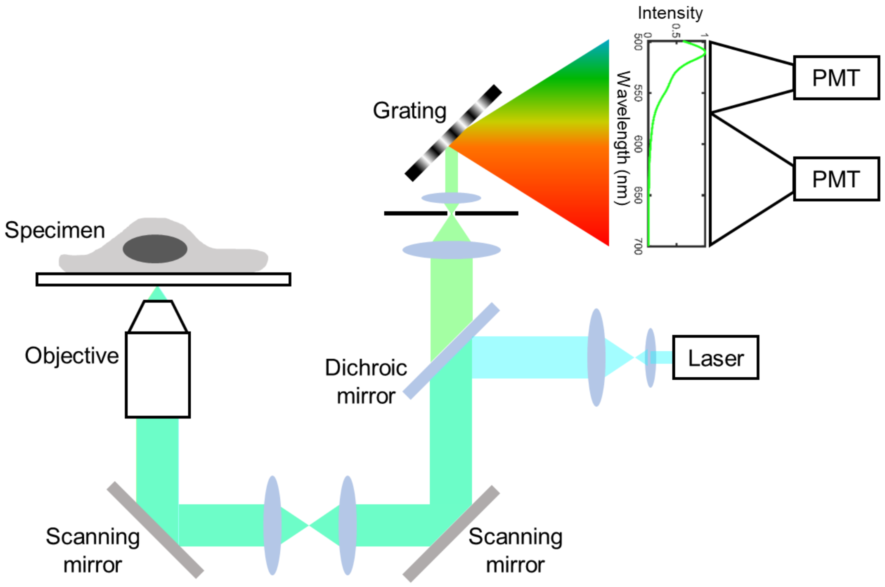

Supplementary Figure 20

Simultaneous low- and high-SNR confocal imaging system. To perform a direct and quantitative validation of performance and accuracy of DeepSeMi, we modified a commercial confocal system for acquiring simultaneous high- and low-SNR cell images. To achieve this, we modified a commercial confocal microscope (Nikon AX), which can acquire hyperspectral images with up to 1 nm spectral resolution through a grating and multiple photomultiplier tubes (PMTs). We enlisted two PMTs for acquiring emitted photons from solitary fluorescent dye/protein. Since each PMT covered different portions of the emission spectrums, the total emitted fluorescent photons were captured by the two PMTs in portions of 6% and 94%, respectively. The captured images from these two PMTs thus formed low- and high-SNR image pairs. The calibration of the SNRs of two PMT arms can be found in Supplementary Fig. 21.

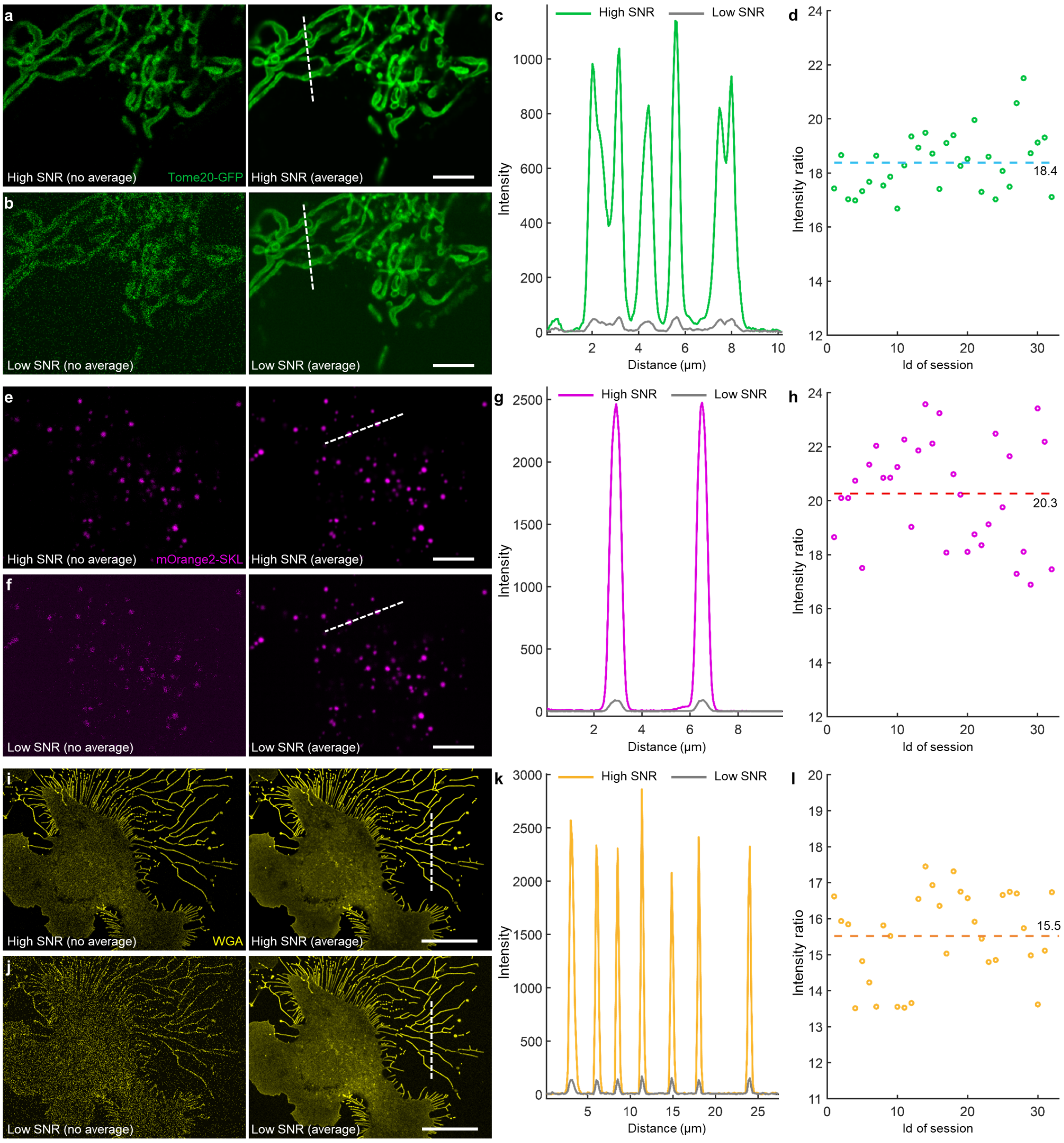

Supplementary Figure 21

Characteristics of the simultaneous low- and high-SNR confocal imaging system. We utilized fixed samples to calibrate the low- and high-SNR simultaneous acquisition system. **a**, The high-SNR frame (left) and averaged 200 high-SNR frames (right) of Tom20-GFP labeled mitochondria. **b**, The low-SNR frame (left) and averaged 200 low-SNR frames (right) of the same Tom20-GFP labeled mitochondria, which was simultaneously captured with high-SNR results in **a**. **c**, Intensity profiles along the white dashed lines in high-SNR (green, **a**) and low-SNR (gray, **b**) images. **d**, Scatter plot of net

intensity ratio between high- and low-SNR images, across 32 sessions. For both high- and low-SNR images, background intensity has already been subtracted and the ratio was calculated between averaged pixels (see Methods in the manuscript for details). In such a way, the intensity ratio acts as an approximation of the ratio of collected fluorescent photons from the high-SNR and low-SNR arms. The average ratio across 32 imaging sessions was 18.4, indicating the high-SNR arm held an 18.4-fold of photon enrichment compared to the low-SNR arm. **e-h** are the same as **a-d** but with imaging results of mOrange2-SKL labeled peroxisomes. The averaged photon enrichment ratio between the high-SNR and low-SNR arms across 32 imaging sessions was 20.3. **i-l** are the same as **a-d** but with imaging results of WGA-labeled L929 cells. The averaged photon enrichment ratio between the high-SNR and low-SNR arms across 32 imaging sessions was 15.5. Scale bars 5 μm in **a**, **b**, **e**, **f**, and 10 μm in **i**, **j**.

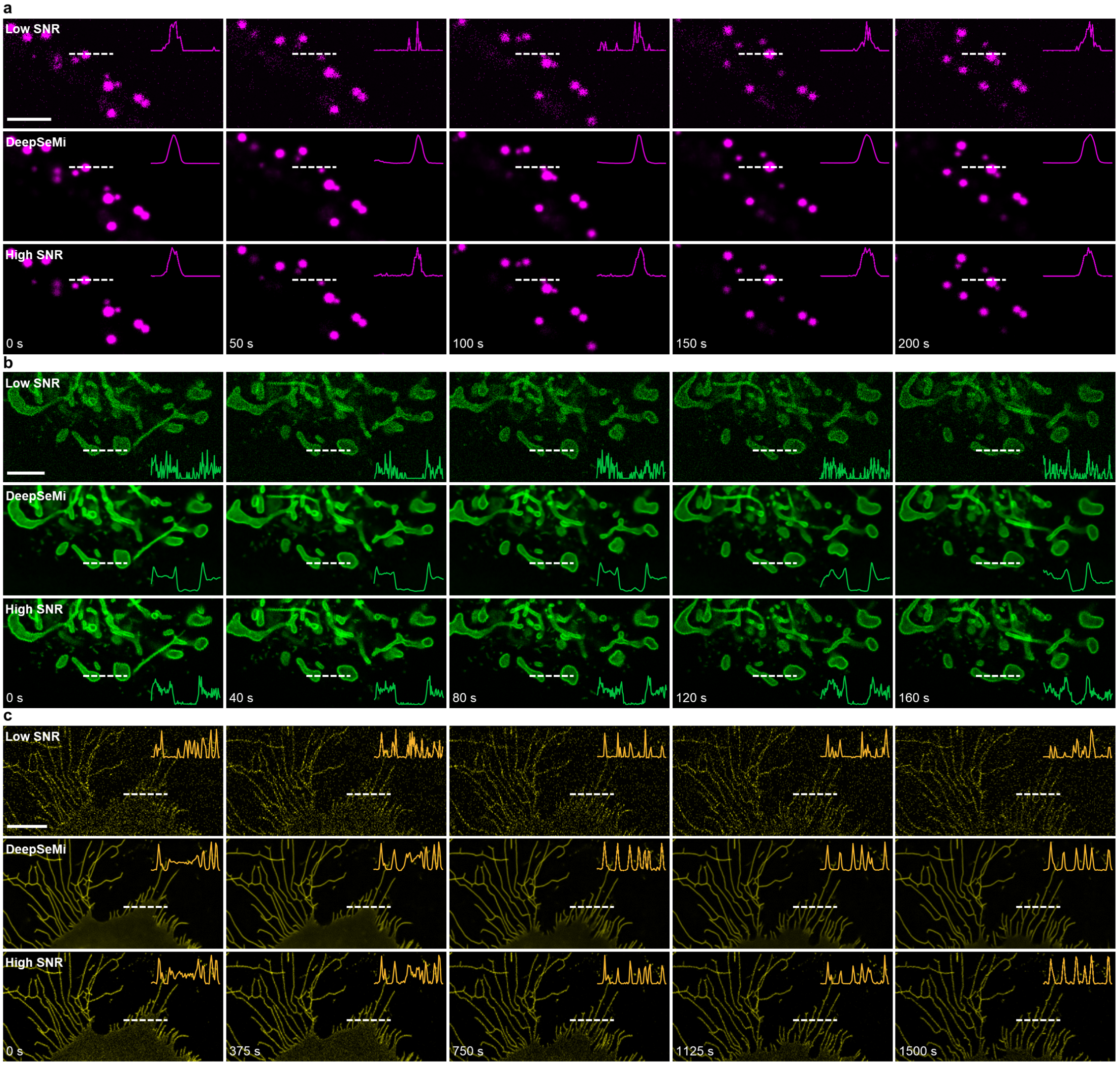

Supplementary Figure 22

Evaluate DeepSeMi on experimental high- and low-SNR confocal fluorescent recordings. a, Evaluation of DeepSeMi over mOrange2-SKL labeled peroxisomes. Low-SNR frames, DeepSeMi recovered frames, and high-SNR frames were presented in different rows, respectively. Five columns represented 5 time points as labeled in the bottom. Intensity profiles across the white dashed lines were decorated in the top right corner for each panel. **b** and **c** are the same as a but with imaging results of Tom20-GFP-labeled mitochondria and WGA-labeled cells, respectively. Scale bar 3 μm in **a**, 5 μm in **b**, and 10 μm in **c**.

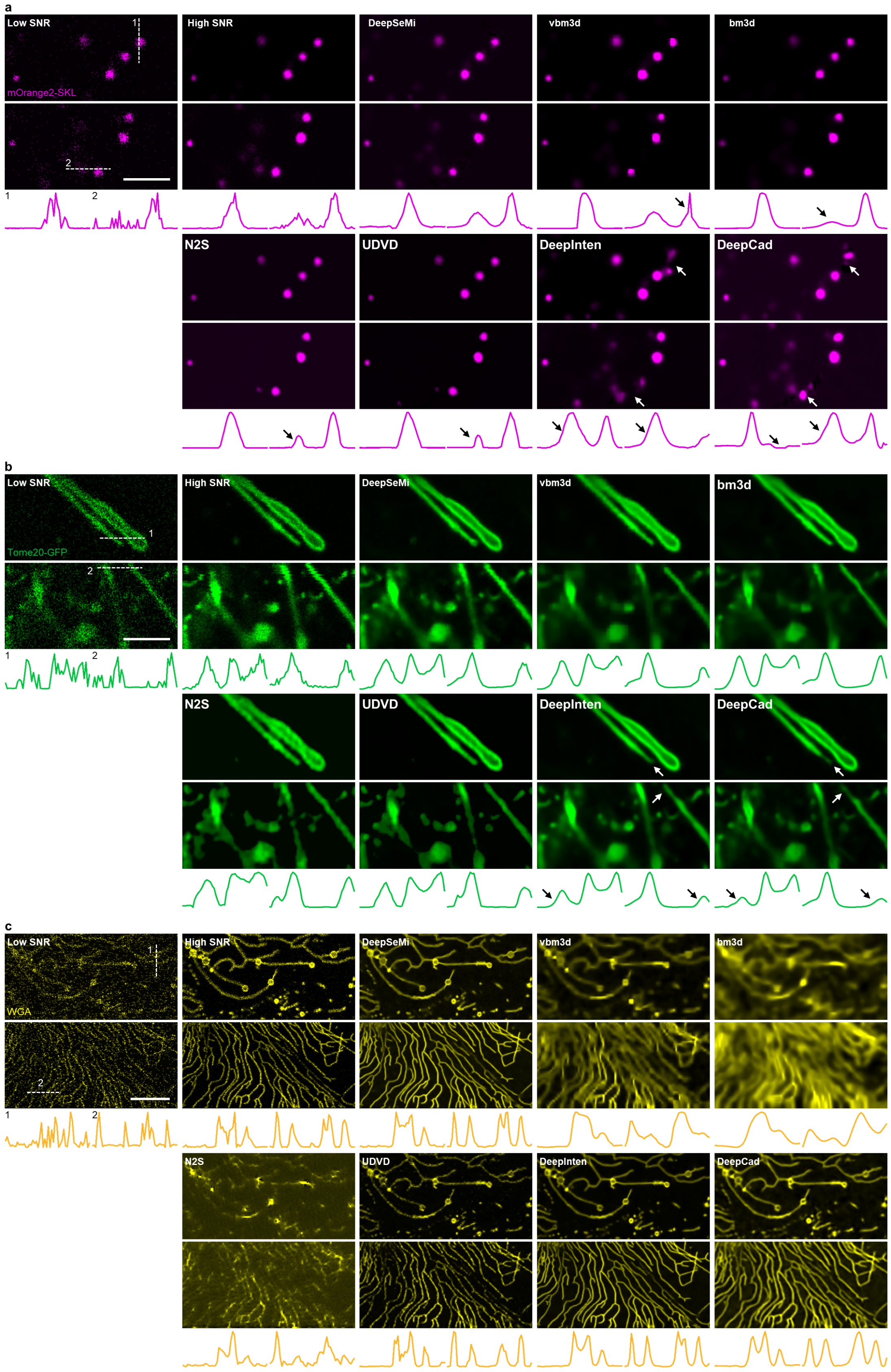

298 **Supplementary Figure 23**

Benchmarking DeepSeMi on experimental high- and low-SNR confocal recordings, part I. After calibrating the simultaneous high- and low-SNR confocal imaging system, we conducted comparison of DeepSeMi and other six denoising methods (VBM3D, BM3D, N2S, UDVD, DeepIntern, and DeepCAD). See Supplementary Fig. 24 for additional comparison with a supervised method, Noise2Void, DivNoising, Probabilistic Noise2Void, and Noise2same. **a**, Comparisons over mOrange2-SKL labeled peroxisomes. Two representative cases were presented in the first and the second rows for each method, respectively. Two intensity profiles at those two images were plotted in the bottom and labeled by numbers. While the peroxisomes were moving, frame-interpolation-based methods (DeepIntern and DeepCAD) generated apparent artifacts that were highly similar with morphologies of peroxisomes (white arrows), which might strongly alternate potential biological conclusions. Black arrows marked intensity artifacts along the white dashed lines across these denoising methods. Among those denoising methods, DeepSeMi achieved mostly accurate denoised results compared to the high-SNR ground truth. Scale bar 3 μm. **b**, Comparison over Tom20-GFP labeled mitochondria. The first row and the second row presented two views of stretching mitochondria. Two intensity profiles at those two frames were plotted in the bottom and labeled by numbers. Frame-interpolation-based methods (DeepIntern and DeepCAD) could not catch the fast dynamics of the stretching mitochondria and generated confusing artifacts, as marked by the white arrows. Black arrows marked intensity artifacts along the white dashed lines across these denoising methods. Among those denoising methods, DeepSeMi achieved mostly accurate denoised results compared to the high-SNR ground truth. Scale bar 3 μm. **c**, Comparison over WGA labeled cells. Two intensity profiles at those two frames were plotted in the bottom and labeled by numbers. Across those methods, VBM3D, BM3D, N2S, UDVD and DeepCAD generated apparent artifacts or uncleaned backgrounds. Results by DeepSeMi and DeepIntern were the closest to the high-SNR ground truth. Scale bar 10 μm.

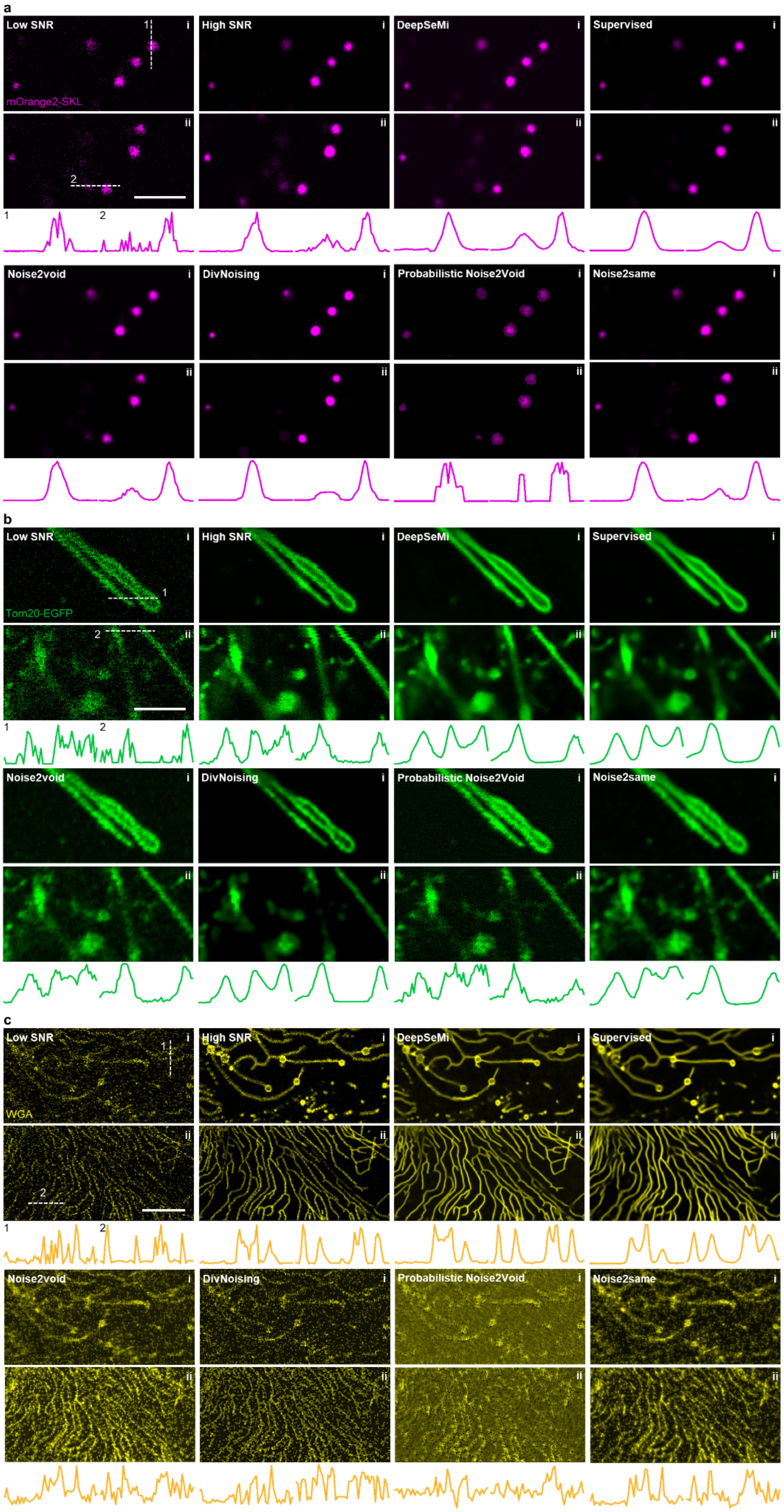

326 **Supplementary Figure 24**

Benchmarking DeepSeMi on experimental high- and low-SNR confocal recordings, part II. After calibrating the high- and low-SNR imaging results, we conducted comparison of DeepSeMi and other 5 denoising methods (supervised method, Noise2Void, DivNoising, Probabilistic Noise2Void, and Noise2same). See Supplementary Fig. 23 for additional comparison with VBM3D, BM3D, N2S, UDVD, DeepIntern, and DeepCAD. **a**, Comparisons over mOrange2-SKL labeled peroxisomes. Two representative cases were presented in the first and the second rows for each method, respectively. Two intensity profiles in those two images were plotted at the bottom and labeled by numbers. Scale bar 3 μm. **b**, Comparison over Tom20-GFP labeled mitochondria. The first row and the second row presented two views of stretching mitochondria. Two intensity profiles at those two frames were plotted at the bottom and labeled by numbers. Scale bar 3 μm. **c**, Comparison over WGA labeled cells. Scale bar 10 μm.

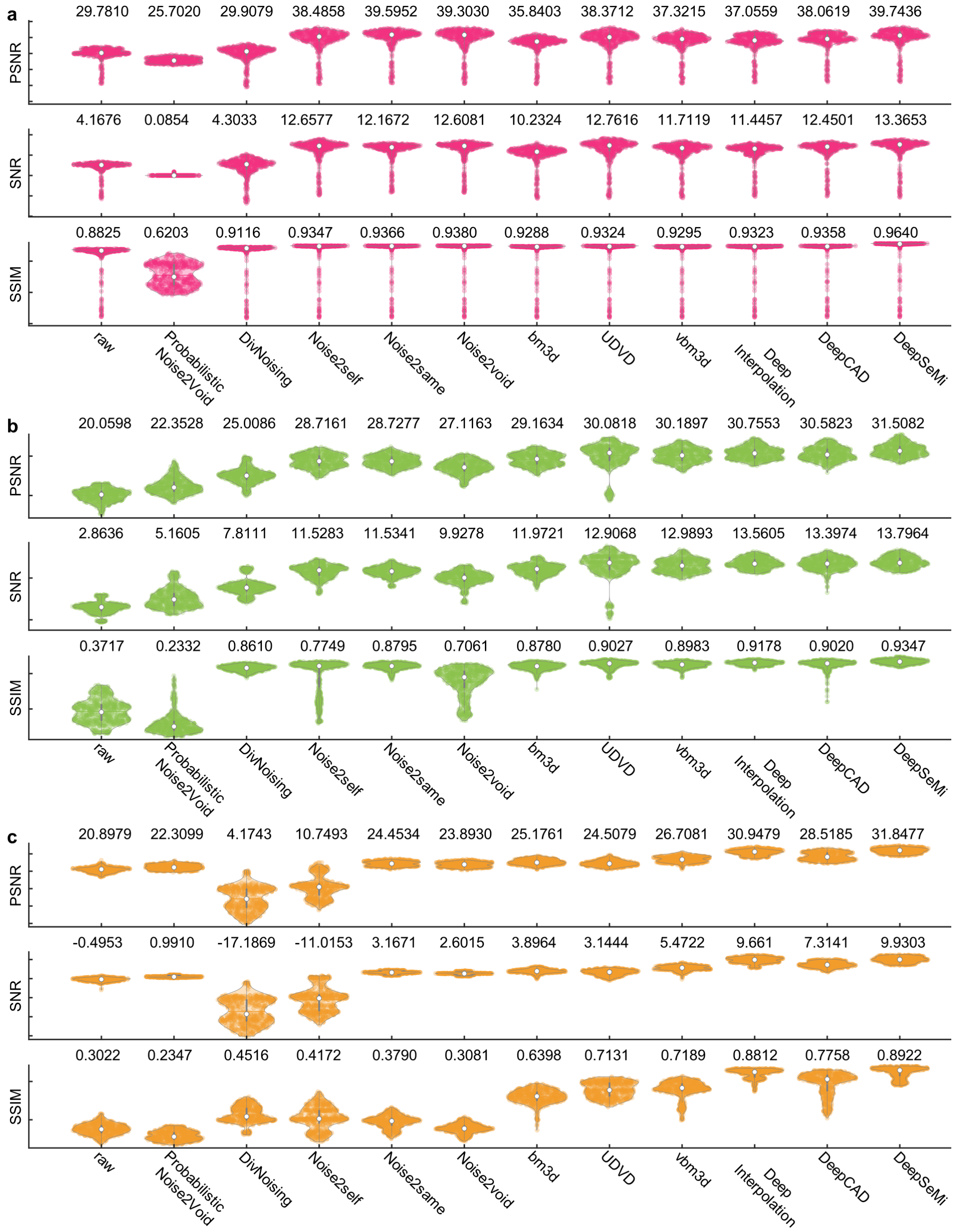

Supplementary Figure 25

Denoising performance benchmark of DeepSeMi and other methods on experimental data procured through simultaneous high- and low-SNR confocal system. a, A comparative analysis of PSNR, SNR, and structure similarity index (SSIM) metrics for probabilistic Noise2Void[7], DivNoising[8], Noise2Self[2], Noise2same[5], Noise2void[6], BM3D[9], UDVD[1], VBM3D[3], DeepInterpolation[11],

DeepCAD[10], and DeepSeMi across peroxisome (mOrange2-SKL labeled) data samples. High-SNR data is established as the ground truth for metric calculations. White circles indicate median values, thin vertical lines represent upper and lower proximal values, while the violin-shaped area displays kernel density estimates of data distribution. n = 600 samples. **b** and **c** replicate the procedure in panel **a** but with imaging of mitochondria (Tom20-GFP labeled) and cell membrane (WGA labeled), respectively.

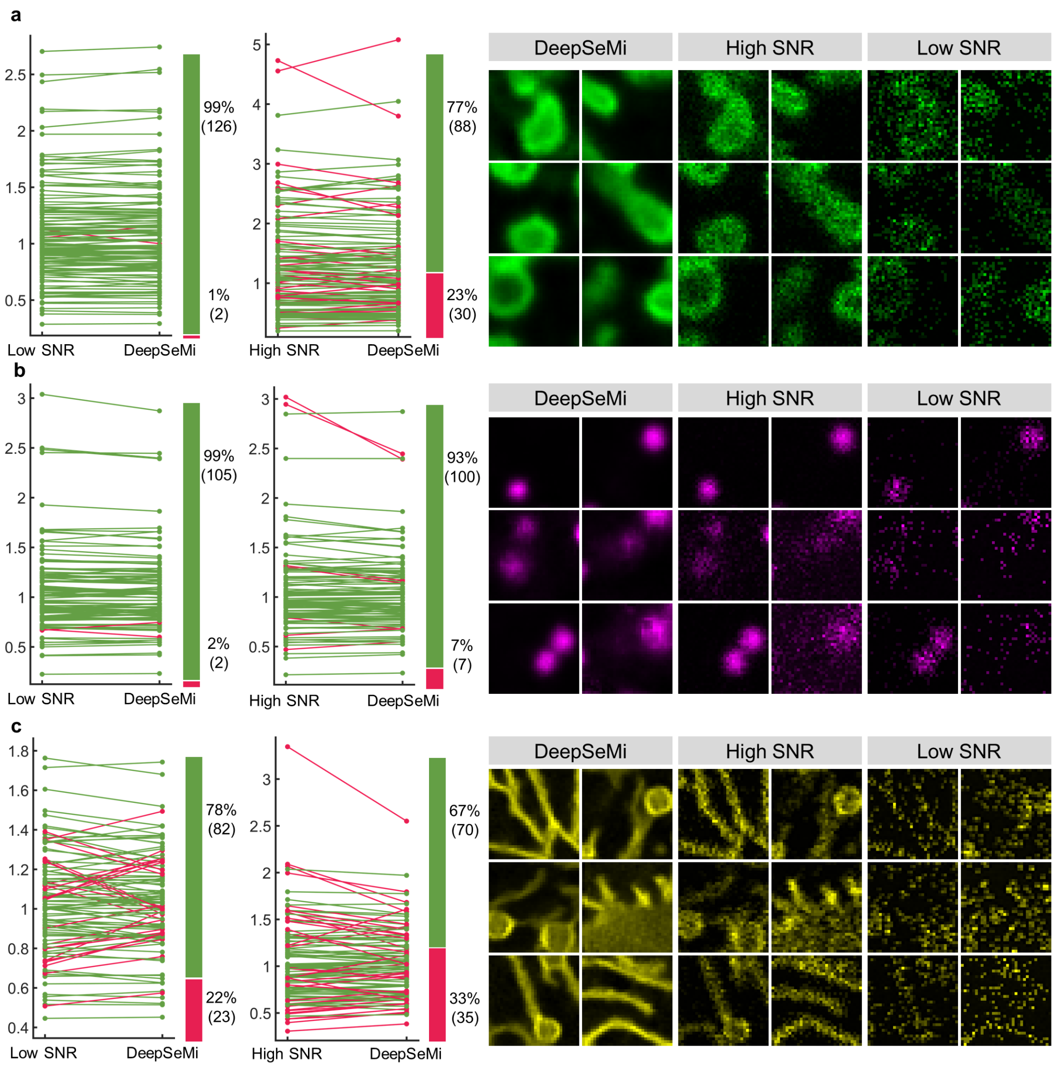

Supplementary Figure 26

Assessment of the ability in maintaining intensity linearity through simultaneous low- and high-SNR confocal imaging system. We built patch groups G_{noise}, $G_{denoise}$ and G_{gt} from low SNR images, high SNR images and DeepSeMi denoised images. Each patch group contains two patches $G_{noise} = \{P^1_{noise}, P^2_{noise}\}$, $G_{denoise} = \{P^1_{denoise}, P^2_{denoise}\}$, $G_{gt} = \{P^1_{gt}, P^2_{gt}\}$, where the size of each patch is 32 pixels. We evaluate the intensity linearity maintaining by computing the ratio of the intensity between the two patches (RITP): $R_{noise} = P^1_{noise}/P^2_{noise}$, $R_{denoise} = P^1_{denoise}/P^2_{denoise}$, $R_{gt} = P^1_{gt}/P^2_{gt}$. We used R_{noise} and R_{gt} as the standard to evaluate the change of $R_{denoise}$. **a**. Lininarity maintaining ability assessment in Tom20-GFP labeled mitochondria. Left, grouped column scatter plot of R_{noise} versus $R_{denoise}$ and R_{gt} versus

$R_{denoise}$. Those exhibiting a change of less than 10% are deemed satisfactory, signifying robust intensity linearity maintenance ability, and are denoted in green in the figure. Those with changes exceeding 10% are indicated in red in the figure. Right, exemplary images of DeepSeMi enhanced captures, high-SNR captures, and low-SNR captures. Three samples are presented in three rows. **b** and **c** are the same as a but with mOrange2-SKL labeled peroxisome and WGA labeled cell membrane, respectively.

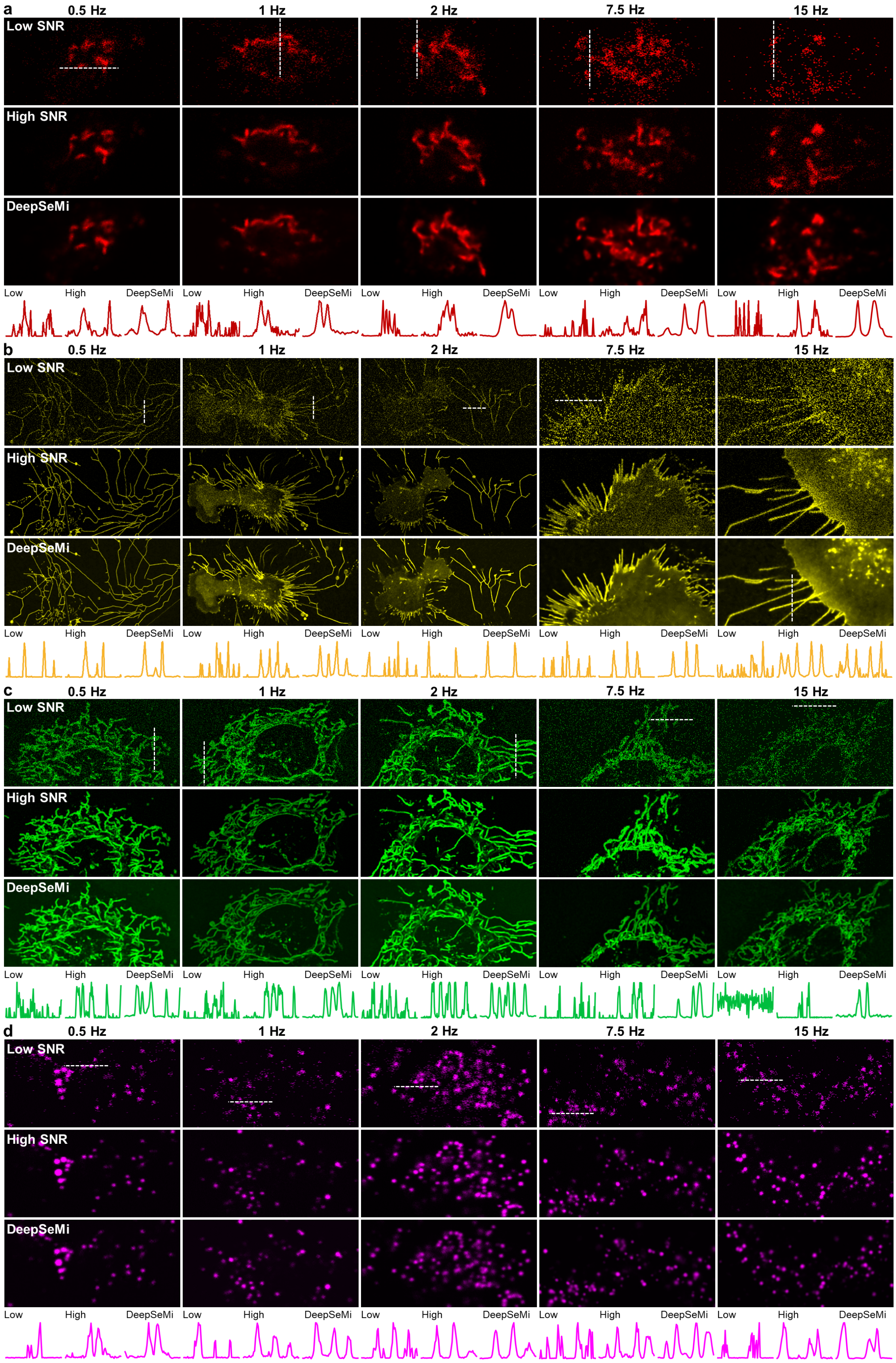

369 **Supplementary Figure 27**

Evaluating denoising performance of DeepSeMi under different imaging speeds through simultaneous low- and high-SNR confocal imaging system. a. Evaluation over SiT-mApple labeled Golgi. Five different imaging speed (0.5Hz，1Hz，2Hz，7.5Hz，15Hz) are evaluated. The sample was imaged through our simultaneous high- and low-SNR confocal imaging system, and corresponding low-SNR, high-SNR, and DeepSeMi enhanced images were exhibited in three rows, respectively. Intensity profiles across three modalities (low-SNR, high-SNR, and DeepSeMi) along the white dashed lines were plotted for evaluation. **b**, **c**, **d** are the same as **a** but with WGA labeled cell membrane, Tom20-GFP labeled mitochondria, and mOrange2-SKL labeled peroxisome, respectively.

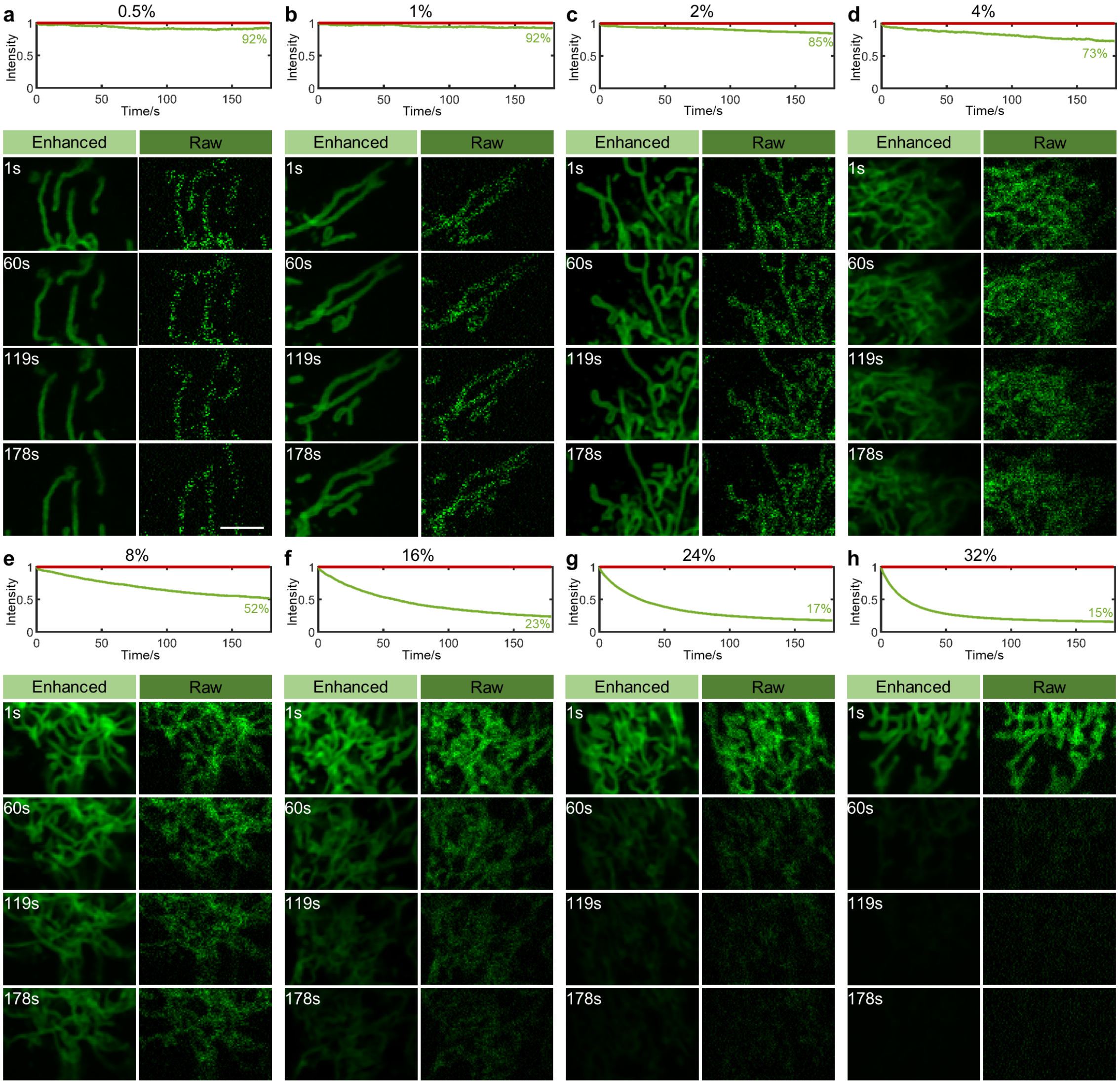

Supplementary Figure 28

Evaluation of photobleaching of mitochondria under different laser dosages. All images are captured under the resonant-scanning mode by commercial confocal microscopy (Nikon A1). **a-h**, Intensity statistics and time-lapse images of mitochondria under different laser intensities (488 nm: 0.5%, 1%, 2%, 4%, 8%, 16%, 24%, 32%). For each laser intensity, 5,400 frames are captured within a 3-minute session window. Top, intensity fluctuations during the laser illumination, where the red line is the reference representing zero photobleaching. Bottom, DeepSeMi enhanced (left) and raw (right) data at four different time points. Scale bar, 5 μm.

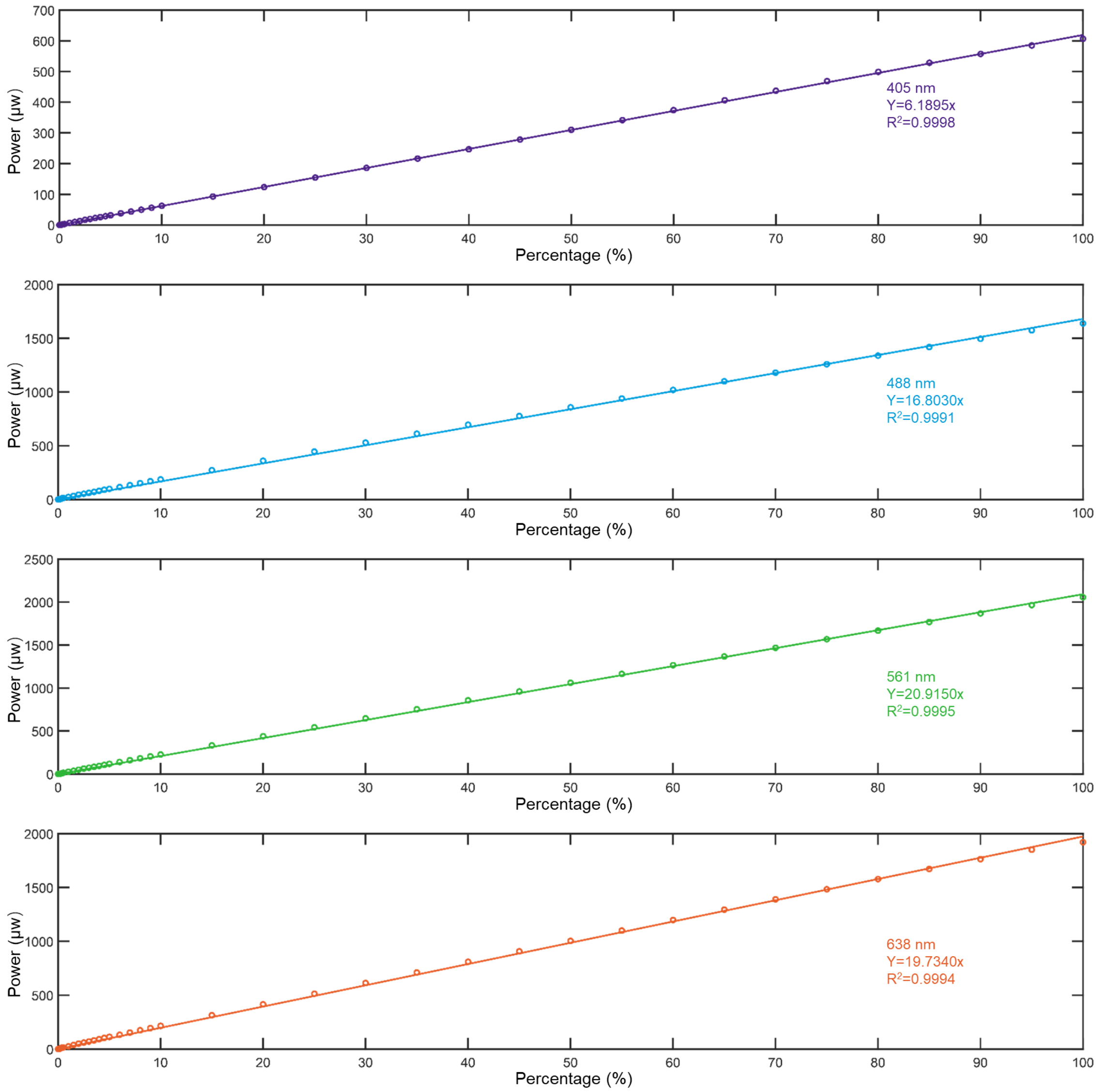

Supplementary Figure 29

Laser power calibration on the Nikon A1 confocal microscopy. We calibrated the laser power of the commercial confocal microscope (Nikon A1) which was used for experiments presented in this research. The laser power was measured at the exit of the objective through a power meter (Thorlabs, PM100D) for four wavelengths separately (405 nm, 488 nm, 561 nm, 638 nm). The linearity of powers across all wavelengths was higher than 0.999.

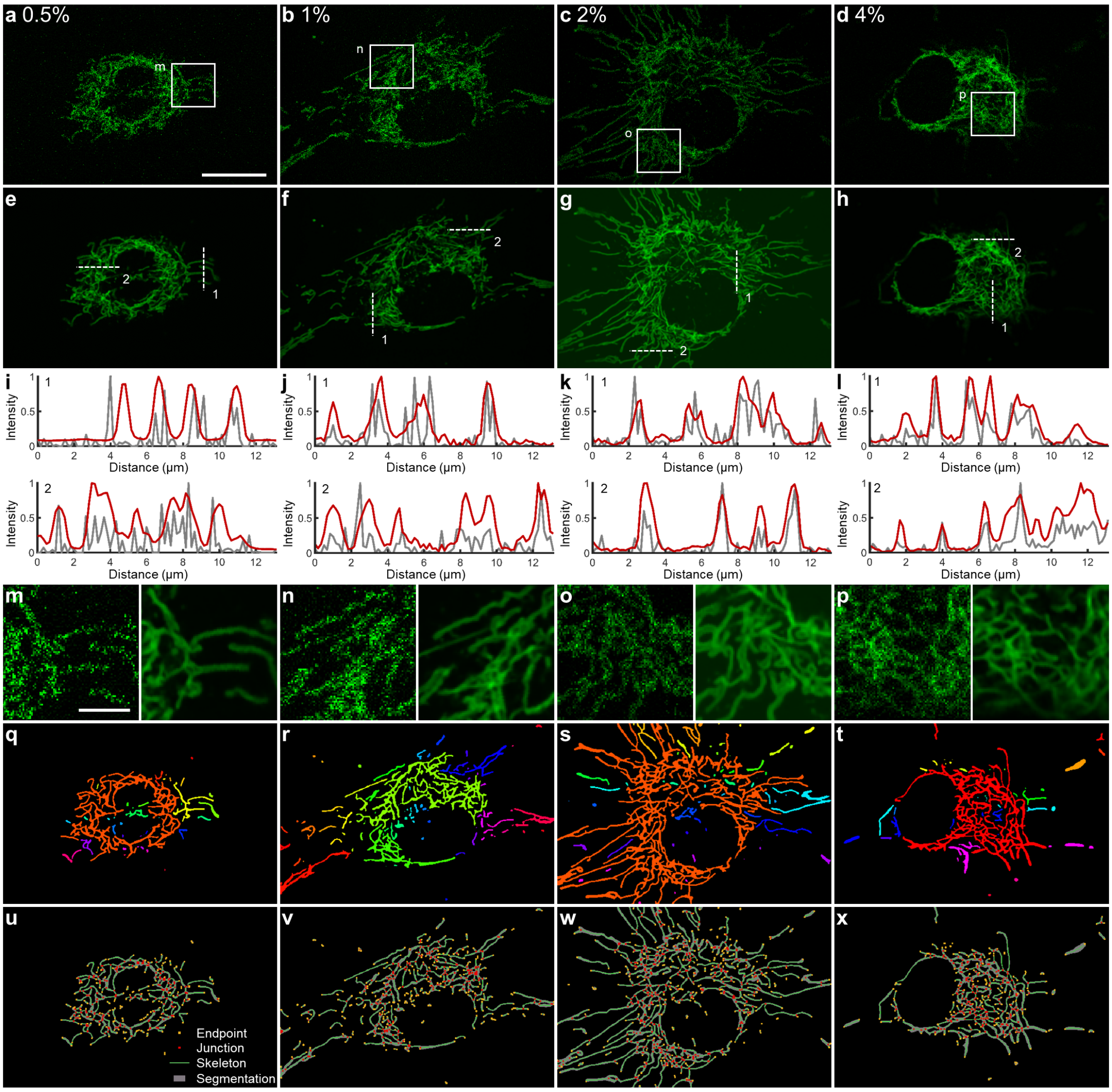

Supplementary Figure 30

DeepSeMi helps automated segmentation and skeletonization of mitochondria under low power dosage. a-d, Raw captures of mitochondria at four light intensities (488 nm:0.5%, 1%, 2%, 4%). Scale bar, 20 μm. **e-h**, DeepSeMi enhanced results of mitochondrial corresponding to **a-d**, respectively. It is obvious that DeepSeMi results in clear structures and cleaner backgrounds across all intensities with delicate mitochondrial details recovered. **i-l**, Intensity profiles along the white dashed line in **e-h**, respectively. The profiles by DeepSeMi enhancement (red) are more reasonable. **m-p**, Zoom-in panels of white boxes in **a-d**, respectively. DeepSeMi reunites fragmented structures due to noise contaminations and unveils rich structures of mitochondria. Scale bar, 5 μm. **q-t**, Instance

segmentation of DeepSeMi enhanced mitochondrial images through a simulation-supervision machine learning algorithm (Methods). Different colors represent different connected regions. **u-x**, Segmentation (gray), skeletonization (green), and key point detection (yellow for the end point, red for junction point) after mitochondrial segmentation (Methods).

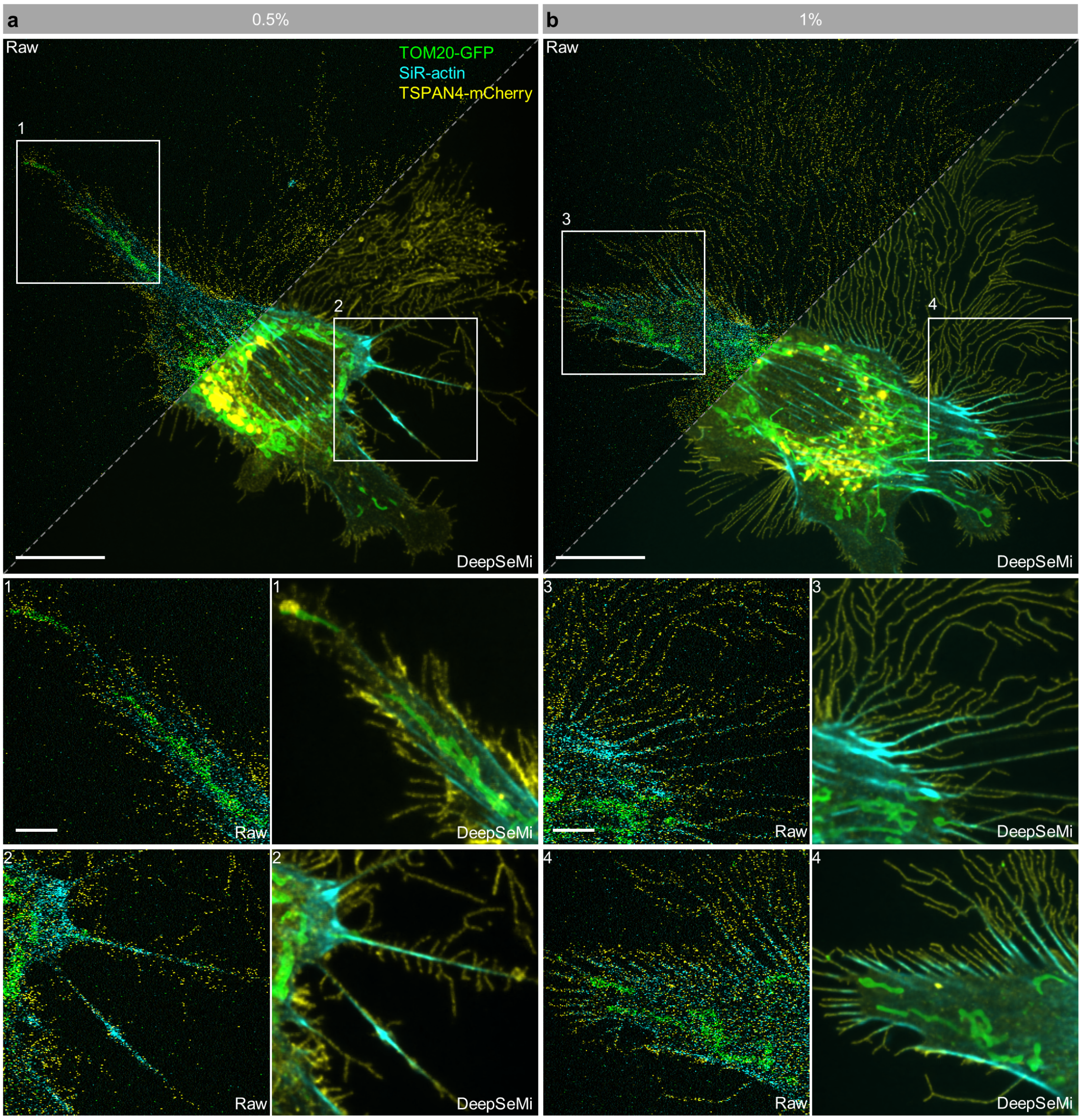

411 **Supplementary Figure 31**

412 **DeepSeMi enables high-SNR imaging of tri-color labeled L929 cells in low light**. **a-**
413 **b**, Raw (left) and DeepSeMi-enhanced (right) tri-color labeled L929 cells in 0.5% and
414 1% laser power (488 nm, 561 nm, and 638 nm), respectively. The second row and the
415 third row presented the zoom-in panels of the white box outlined area in the raw (left)
416 and DeepSeMi-enhanced (right) global view global. Scaler bars are 20 μm in the global
417 views, and 5 μm in the zoom-in views.

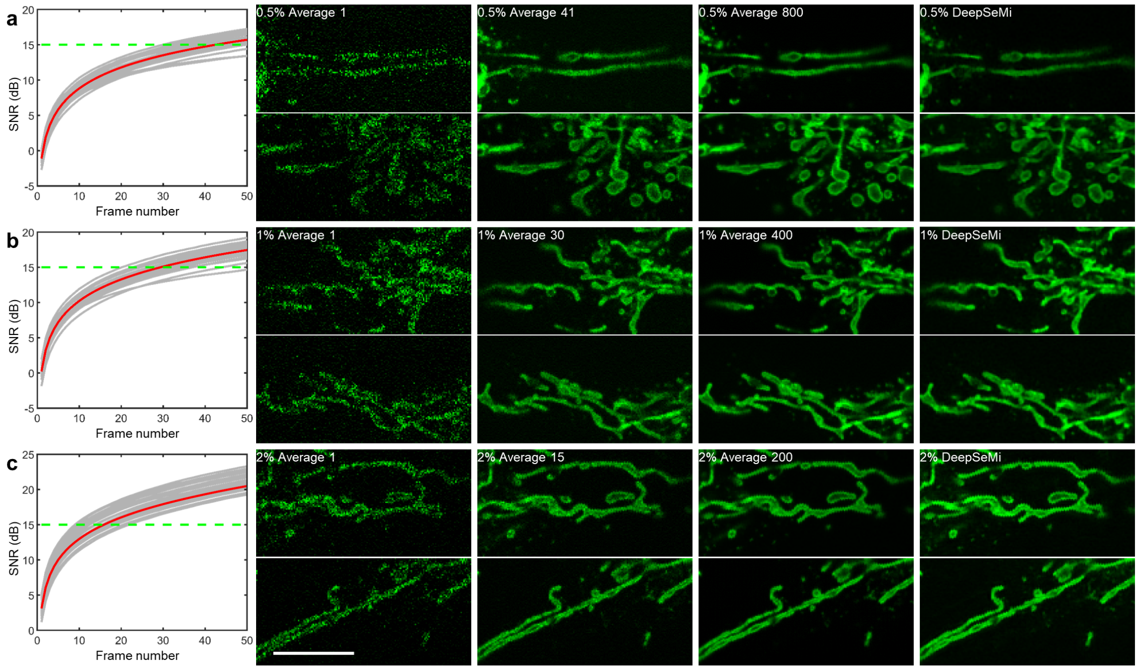

Supplementary Figure 32.

15-fold increment of photon budgets by DeepSeMi.

We designed a special experiment to calibrate the photon budge enhancement through DeepSeMi in a commercial confocal microscope, which is defined as the multiplication of excitation power in raw captures while reaching the same SNR after DeepSeMi enhancement. To acquire the ground truth image in the experiment, we averaged 800 frames of statistic mitochondria at extremely low excitation laser intensity (0.5% at 488 nm). With the synthetic ground truth, we found that DeepSeMi-enhanced mitochondria with a single frame at 0.5% excitation power reached 15 dB SNR. Towards the bar of 15 dB, we found at least 41 raw frames were required to produce 15 dB SNR through averaging **a**. Considering the fluorescence yield are linearly proportional to the one-photon excitation power, the number of raw frames used for averaging to some extent represents the multiplication of excitation power for raw capture to catch up with the imaging quality of DeepSeMi enhanced results. We repeated the same procedure for excitation power 1% **b** and 2% **c**. We found the number of enlisted raw frames for reaching 15 dB gradually decayed to 30 frames **b** and 15 frames **c** as the excitation power increased to 1% and 2%, respectively. Given the fact that DeepSeMi achieves higher SNR

as the excitation power, it is safe to state that DeepSeMi increase the photon budget at least 15 times.

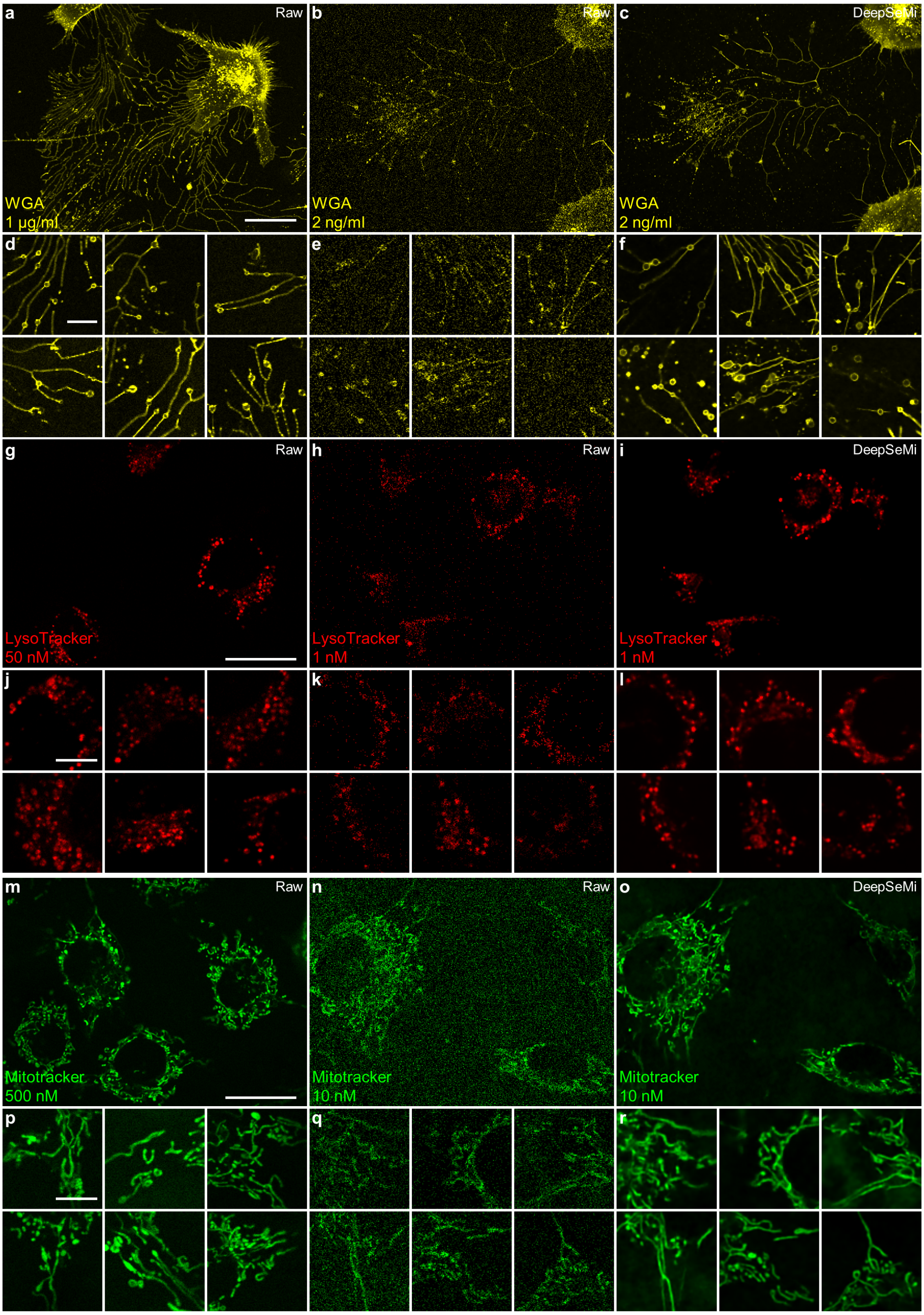

437 **Supplementary Figure 33**

DeepSeMi significantly enhances organelle imaging results with the dye dilution. a, The raw confocal imaging results of cells labeled by WGA with a standard concentration (1 μg/ml). The Nikon A1 was at galvano mode and the excitation laser was at 488 nm (2% power intensity). Scale bar, 30 μm (3 independent trials, each covering more than 10 cells). **b**, Imaging results of the same kind of cells and in the same condition as **a** but labeled by WGA with a diluted concentration (2 ng/ml, 500 times diluted compared to **a**). **c**, DeepSeMi enhanced results of **b**. **d**, Magnified views about migrasomes from raw recordings with standard WGA concentration as in **a**. Scale bar, 10 μm. **e**, Magnified views about migrasomes from raw recordings with 500 times diluted WGA as in **b**. **f**, DeepSeMi enhanced results in **e**. **g**, The raw confocal imaging results of cells labeled by LysoTracker with a standard concentration (50 nM). The excitation laser was at 638 nm (3% power intensity). Scale bar, 30 μm (3 independent trials, each covering more than 10 cells). **h**, The imaging results of the same kind of cells and in the same condition as **g** but labeled by LysoTracker with a diluted concentration (1 nM, 50 times diluted). **i**, The DeepSeMi enhanced results of **h**. **j**, Magnified views about lysosomes from raw recordings with standard LysoTracker concentration in as **g**. Scale bar, 10 μm. **k**, Magnified views about lysosomes from raw recordings with 50 times diluted LysoTracker as in **h**. **l**, DeepSeMi enhanced results in **h**. **m**, The raw confocal imaging results of cells labeled by MitoTracker with a standard concentration (500 nM). The excitation laser was at 561 nm (0.5% power intensity). Scale bar, 30 μm (3 independent trials, each covering more than 10 cells). **n**, The imaging results of the same kind of cells and in the same condition as **m** but labeled by MitoTracker with a diluted concentration (10 nM, 50 times diluted). **o**, The DeepSeMi enhanced results of **n**. **p**, Magnified views about mitochondrial from raw recordings with standard MitoTracker concentration as in **m**. Scale bar, 10 μm. **q**, Magnified views about mitochondrial from raw recordings with 50 times diluted MitoTracker concentration as in **n**. **r**, DeepSeMi enhanced results in **n**.

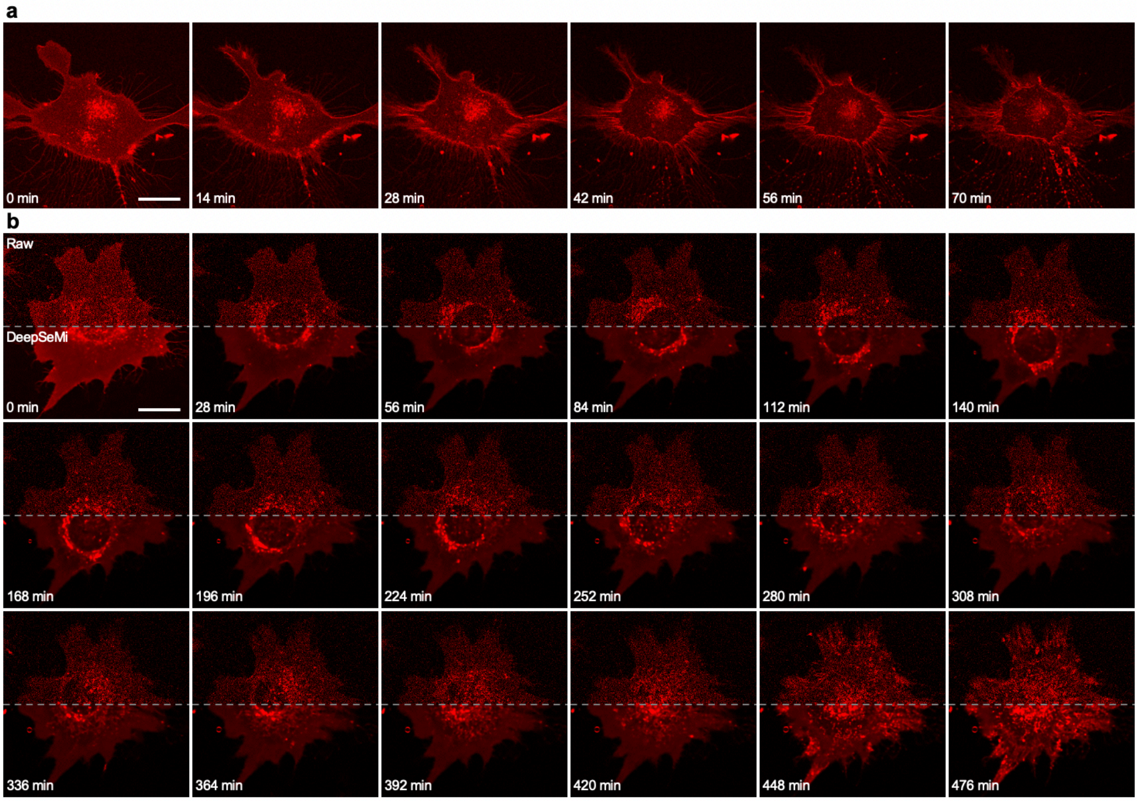

Supplementary Figure 34

DeepSeMi strongly reduced phototoxicity on imaging FM4-64 labeled cells. a, Imaging FM464-labelled L929 cells with 10% laser power at 561 nm in 21 seconds frame intervals. The cell death appeared in ~70 minutes after the start of the imaging session, probably because of phototoxicity. **b**, Imaging FM4-64-labelled L929 cells with 0.5% laser power at 561 nm in 21 seconds frame intervals. At the cost of manifest noise and contaminations, phototoxicity was strongly reduced and the necrosis due to phototoxicity appeared in ~476 minutes after the start of the imaging session, as shown in the top part of each panel. On the other hand, the DeepSeMi enhancement not only inherited the privilege of low phototoxicity but also enabled high-fidelity recovery that was comparable with the results from high power dosage in **a**, as shown in the bottom part of each panel. Scale bar 20 μm.

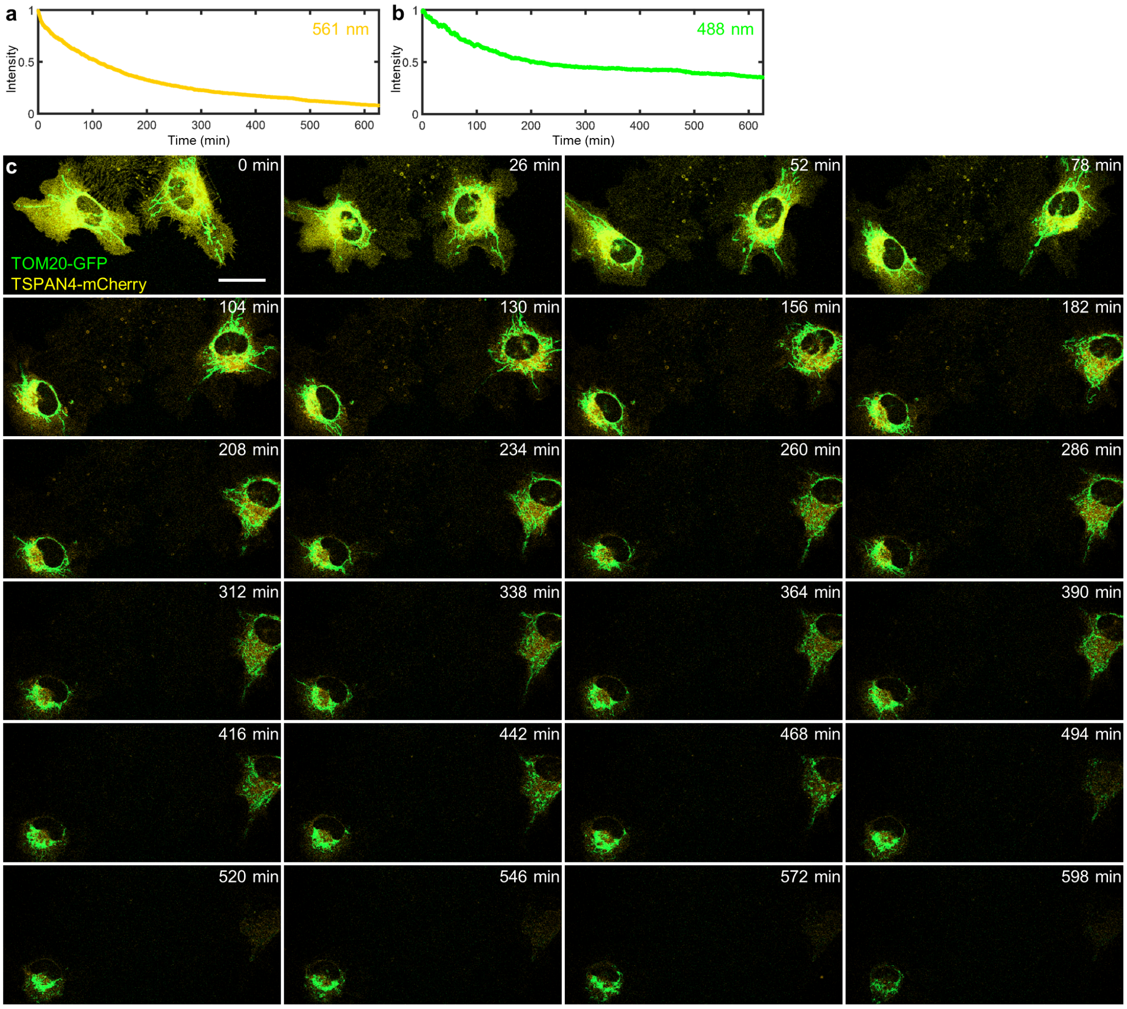

Supplementary Figure 35

Significant photobleaching induced by dual-color confocal imaging. a-b, Normalized fluorescence intensity over continuous 10 hours confocal imaging under 488 nm (right) and 561 nm excitation (left), respectively. 0.36 mW 488 nm and 0.44 mW 561 nm lasers were used for imaging dual-labeled L929 cells at 1.92Hz. **c**, Exemplary time-lapse dual-color images at different time points over 10 hours of imaging clearly show photobleaching. All images are raw captured without DeepSeMi enhancement. Scale bar, 30 um.

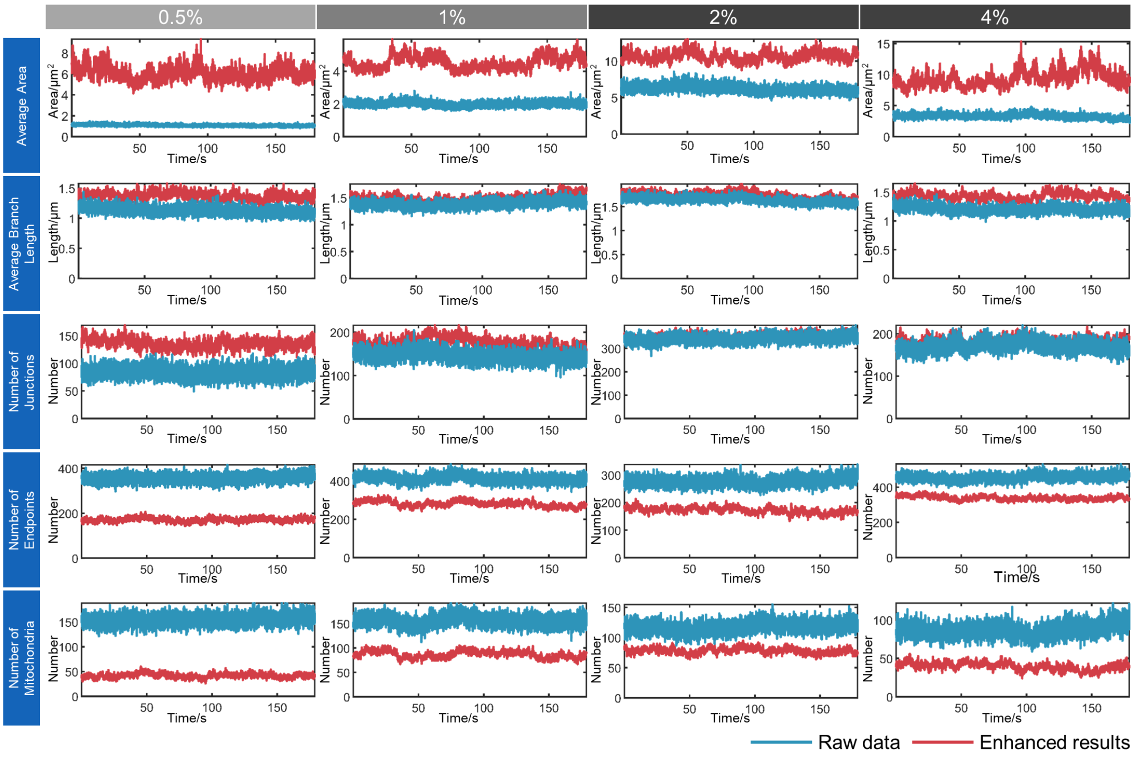

Supplementary Figure 36

Statistics of mitochondrial segmentation and skeletonization under different illumination powers with and without DeepSeMi enhancement. The average area, branch length, number of junctions, number of endpoints, and number of mitochondria were calculated before (blue) and after DeepSeMi enhancement (red) for a 180-seconds imaging session at 30 Hz. Since the DeepSeMi effectively reunited fragment mitochondria under noise contamination, the average area, branch length, and the number of junction points are increased after DeepSeMi enhancement, while the number of endpoints and the number of mitochondria are accordingly reduced.

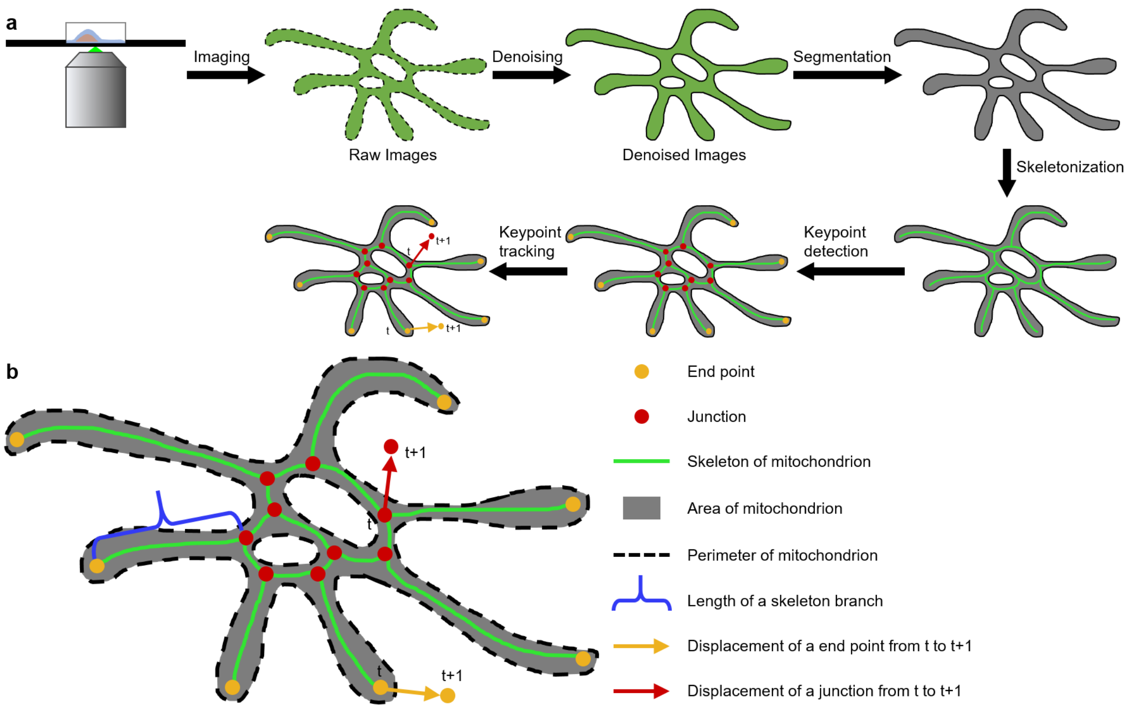

Supplementary Figure 37

Automated analysis of recorded mitochondria with DeepSeMi enhancement. **a**, The captured videos of mitochondria are firstly denoised by DeepSeMi to remove noise contaminations, and then segmented with a simulation-supervision machine learning algorithm [12]. The binary mask is then skeletonized (Methods) with key points (including the end point, junction point) highlighted. Those key points are used for tracking the motion of the mitochondria. **b**, Illustrations of extracted features and associated measurements from the mitochondria, including the end point, junction point, skeleton, area, perimeter, and branch length.

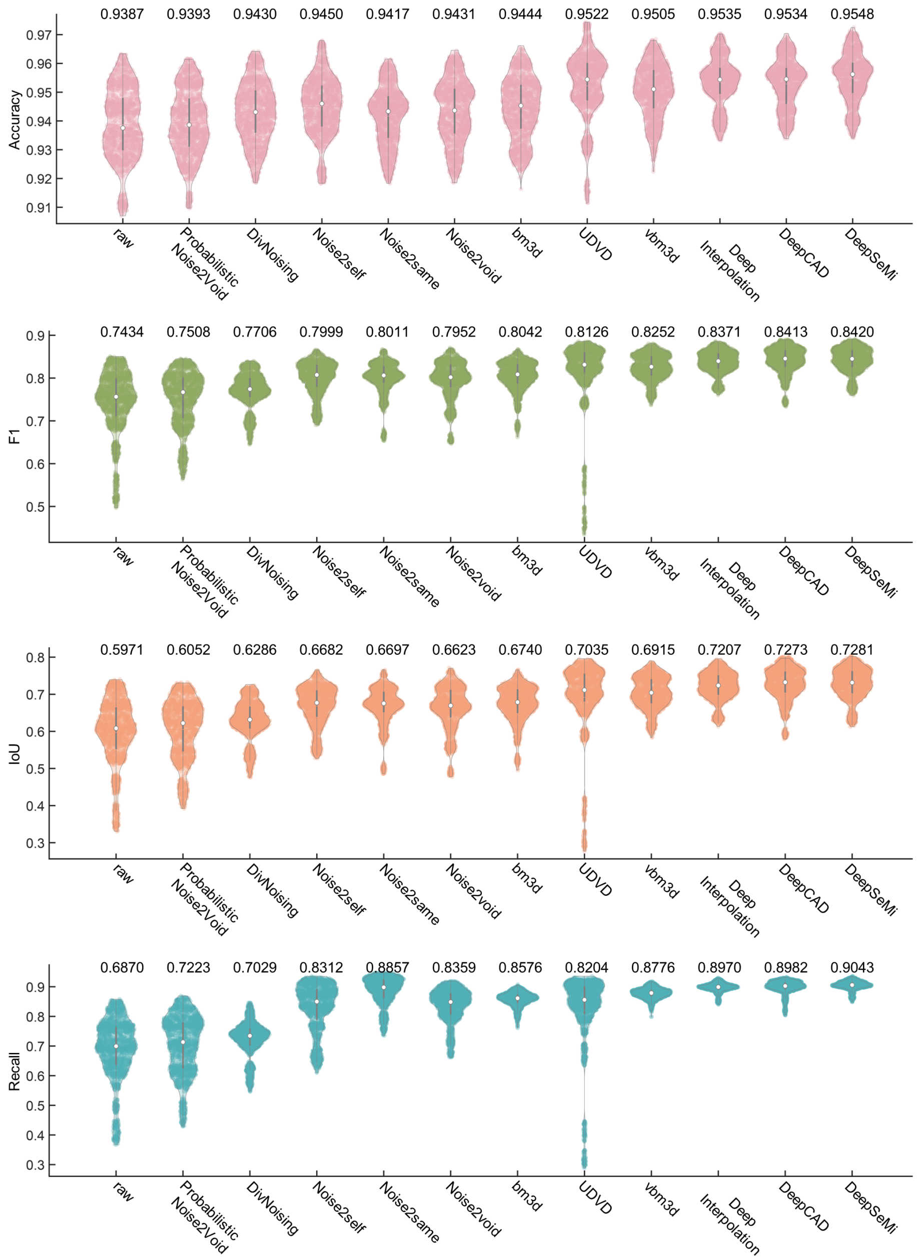

Supplementary Figure 38

Cellular segmentation benchmark of DeepSeMi and other denoising methods enhanced experimental data produced by simultaneous high- and low-SNR confocal system. Segmentation accuracy, F1, intersection over union (IoU), and recall scores of probabilistic Noise2Void[7], DivNoising[8], Noise2Self[2], Noise2same[5] , Noise2viod[6], BM3D[9], UDVD[1], VBM3D [3], DeepInterpolation[11], DeepCAD[10], and DeepSeMi across EGFP-labeled mitochondrial data. The

segmentation derived from high-SNR data is established as the ground truth for the computation of these metrics. White circle: median. Thin vertical lines: upper and lower proximal values. Violin-shaped area: kernel density estimates of data distribution. n = 600 samples. Segmentation methods are described in Methods.

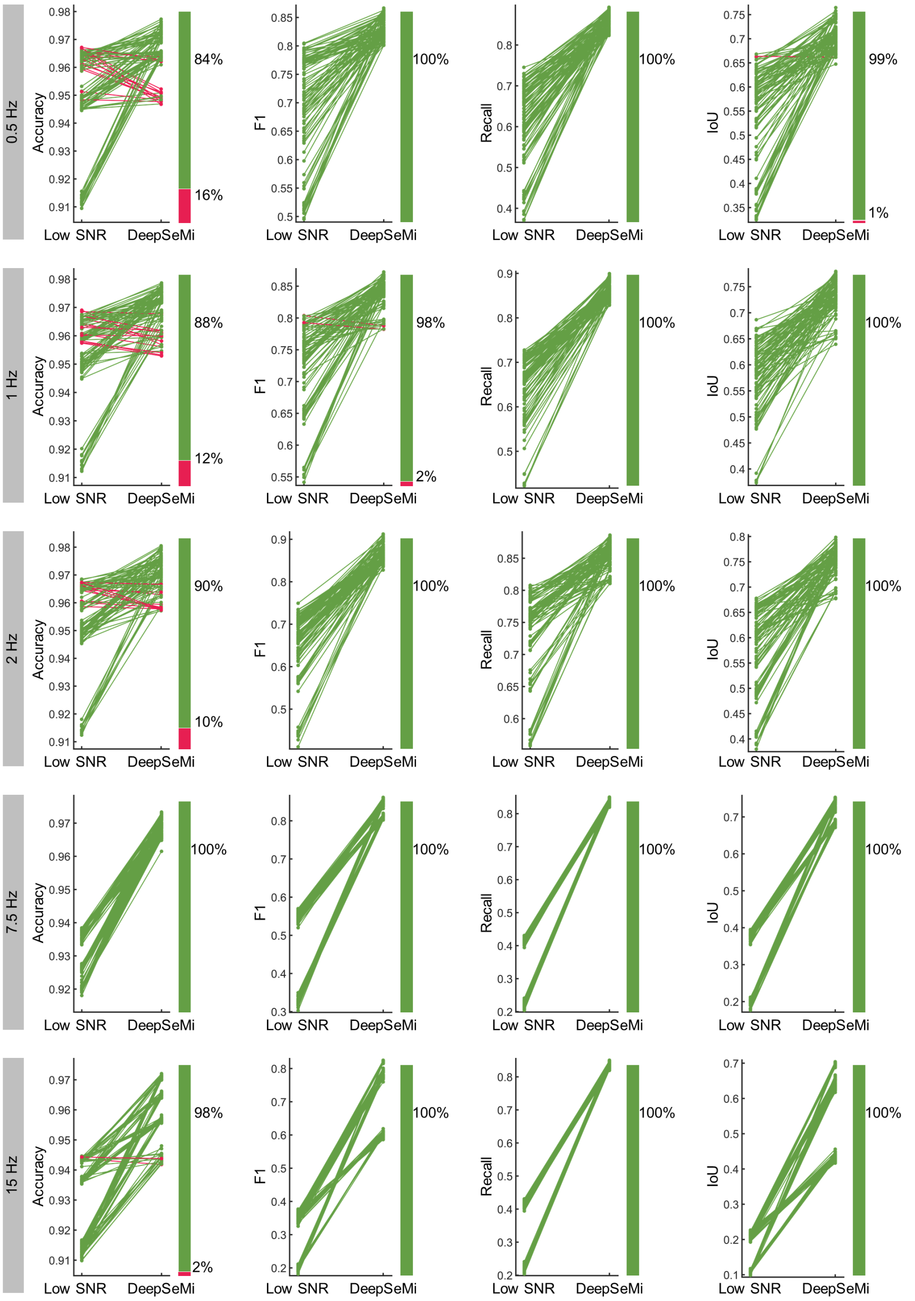

Supplementary Figure 39

Evaluating of DeepSeMi enhanced segmentation performance on mitochondria under different imaging speeds with simultaneous low- and high-SNR confocal imaging system. Five different imaging speeds (0.5Hz, 1Hz, 2Hz, 7.5Hz, 15Hz) are presented as different rows. For each imaging speed, segmentation accuracy, F1, recall, and intersection over union (IoU) scores are evaluated. The high-SNR data segmentation is utilized as the ground truth for these metrics calculations. Each panel displays the

fluctuation in scores, with individual lines representing distinct samples out of the 200 totals. The colour code signifies an increase in segmentation scores (green) or a decrease (red). The segmentation methods are elaborated in the manuscript's Methods section.

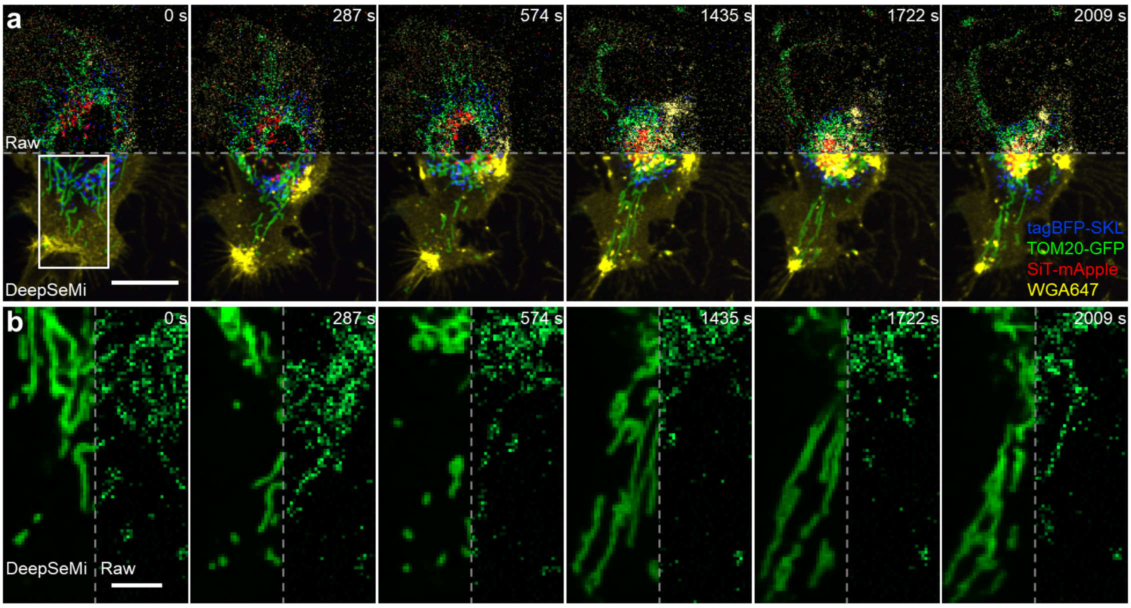

Supplementary Figure 40

DeepSeMi-enhanced imaging results of L929 cells treated with Lat-A. a, Raw (top) and DeepSeMi-enhanced (bottom) long-term imaging of L929 cells with four organelles labeled colorfully (TOM20–GFP, WGA647, TagBFP–SKL, and SiT-mApple). Latrunculin-A (Lat-A) was added to the cell culture medium at 0s to decompose the cytoskeleton (Methods). Scale bar 20 μm. **b**, Mitochondria deformation during 33 minutes-long time-lapse imaging after treatment with Lat-A. For each panel, the left part represents DeepSeMi enhancement and the right panel represents the raw image. Scale bar 5 μm.

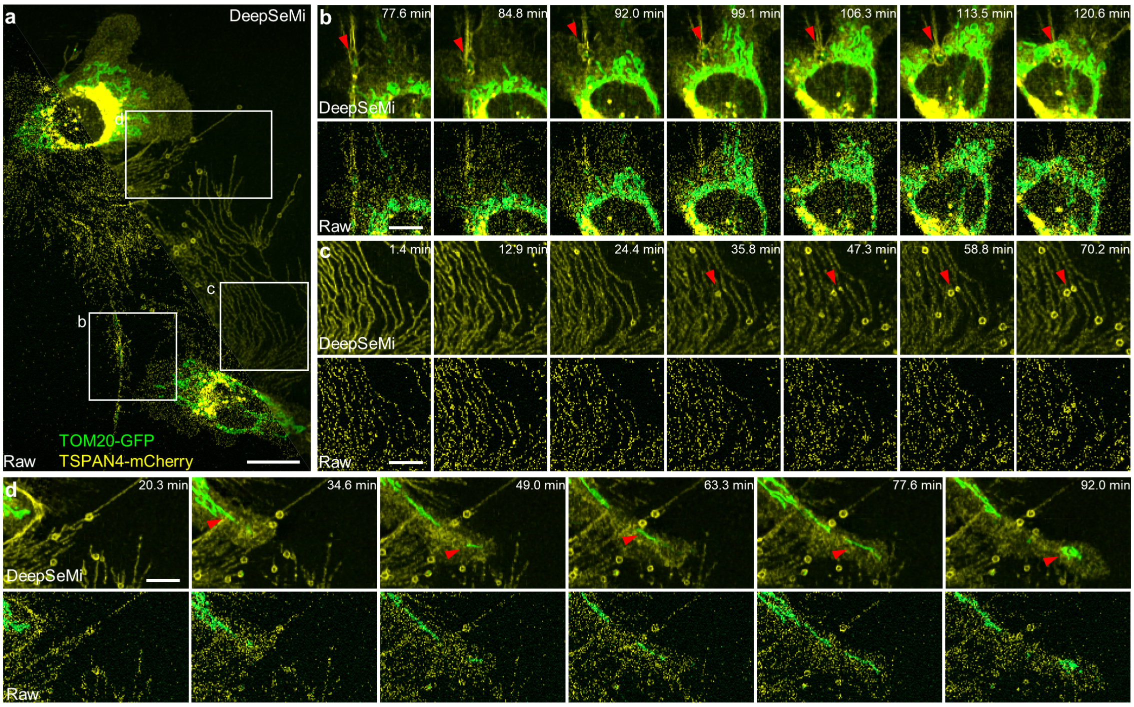

Supplementary Figure 41

DeepSeMi unveiled migrating cells interacting with a migrasome, producing migrasomes, and expelling mitochondria in a low light dosage. a, Raw (left) and DeepSeMi enhanced (right) observation of two-cell interactions. Cells with mitochondria (green, TOM20-GFP) and migrasomes (yellow, TSPAN4-mCherry) labeled were imaged at 95.1 μW for 2 hours at 1.16 Hz. Scale bar, 20 μm. **b**, Time-lapse process of cell interacting with a migrasome (marked by red arrows) during migration by DeepSeMi enhanced (top) and raw (bottom) captures. The migrasome is almost invisible in the raw movie. Scale bar, 10 μm. **c,** Time-lapse process of a cell producing migrasomes during migration by DeepSeMi enhanced (top) and raw (bottom) captures. The retraction fibers were produced when the cell was crawling, and spherical migrasomes (marked by red arrows) were generated on the retraction fibers by the regulation of the cell. Scale bar, 10 μm. **d**, Time-lapse process of cell expelling mitochondria (marked by red arrows) during migration by DeepSeMi enhanced (top) and raw (bottom) captures. Scale bar, 10 μm.

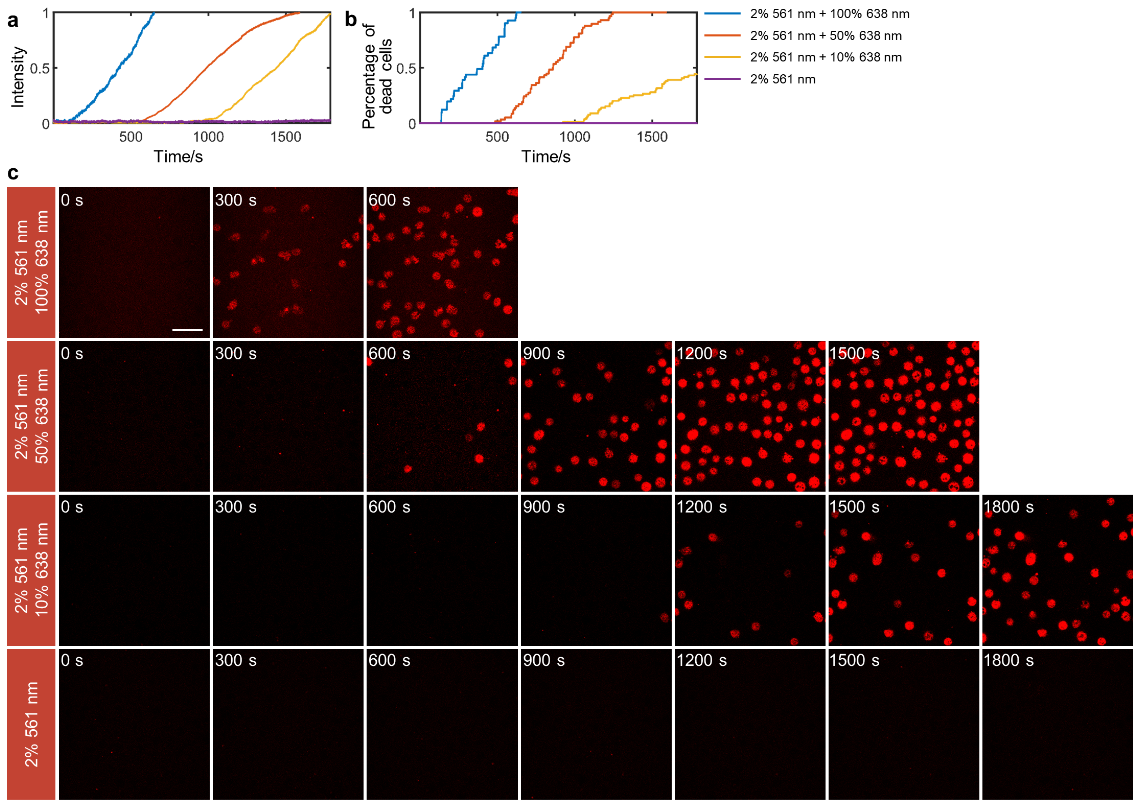

Supplementary Figure 42

Evaluation of phototoxicity in imaging *Dictyostelium* cells. The *Dictyostelium* cells were simultaneously illuminated by the 638 nm laser and the 561 nm laser. The 638 nm laser was used for generating phototoxicity on *Dictyostelium* cells. The 561 nm laser was used for imaging to evaluate the phototoxicity brought by 638 nm illumination. When *Dictyostelium* cells died because of phototoxicity, the permeability of the membrane changed and the propidium iodide in the micro-environment entered into *Dictyostelium* cells which facilitated fluorescence imaging under 561 nm excitation. Four laser powers at 640 nm were assessed (0%, 10%, 50%, 100%). **a**, Statistics of fluorescence intensity change under 561 nm excitation during imaging as a function of time in four conditions. The fluorescence intensity is normalized into 0 to 1. **b**, The number of *Dictyostelium* cell deaths during imaging as a function of time in four conditions. The number of cell deaths is normalized into 0 to 1, which is highly correlated with curves in **a**. **c**, Exemplary results of fluorescence imaging of *Dictyostelium* cells under 561 nm excitation during phototoxicity experiments at different time points. Scale bar, 20 μm.

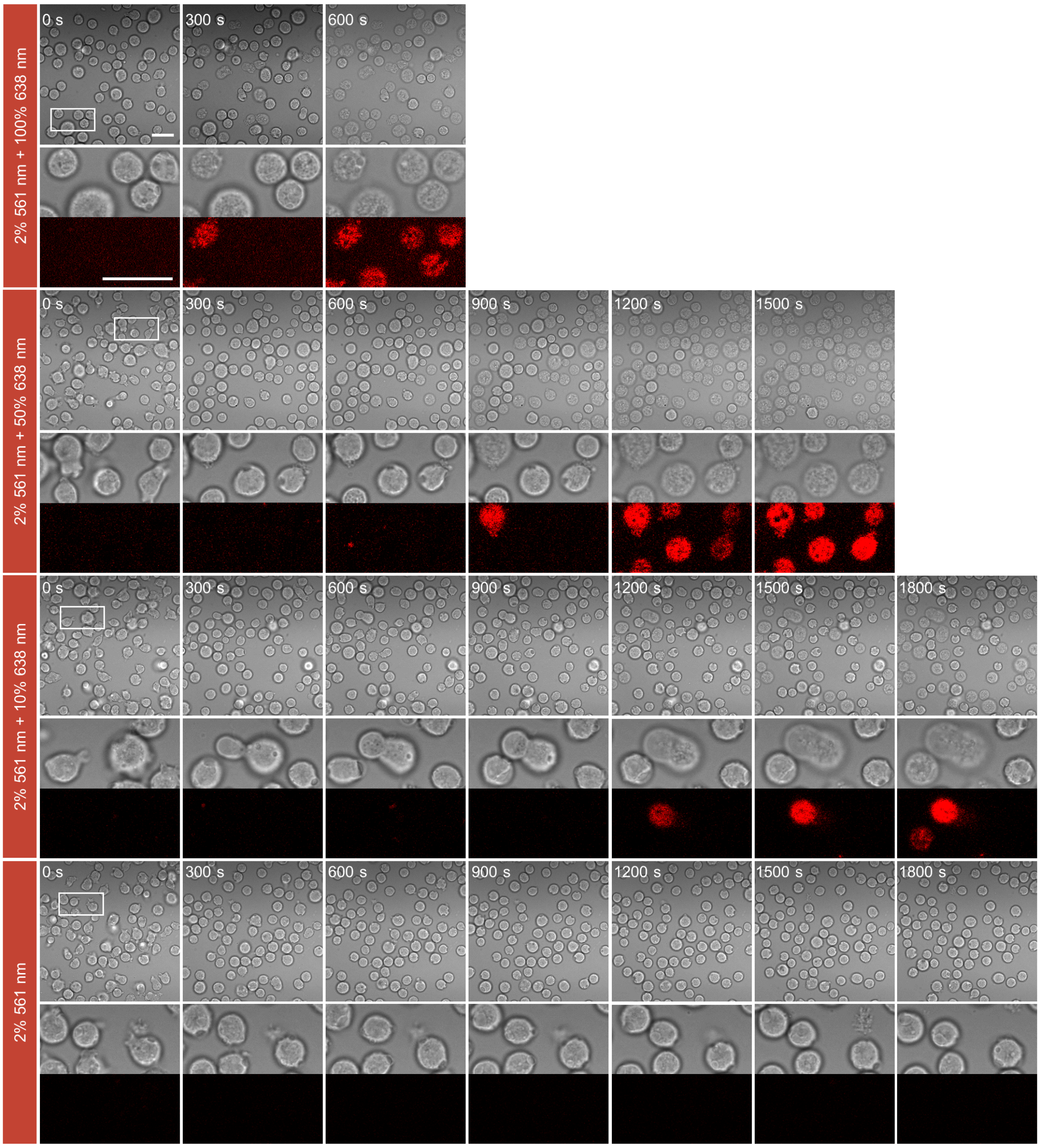

Supplementary Figure 43

Evaluation of phototoxicity in imaging *Dictyostelium* cells with a bright-field microscope imaging. The imaging setup is the same as Supplementary Figure 42. The boundaries of the cells lose sharpness as the cell suffers phototoxicity, which provides another clue to monitor the health status of the cell. Four laser powers at 638 nm were assessed. For each power, the first row shows a global view of the bright field microscope capture, and the top part of the second row shows a zoom-in image of the white box in

the global view, and the bottom part of the second row shows the corresponding fluorescence image under 561 nm excitation. Scale bar, 20 μm.

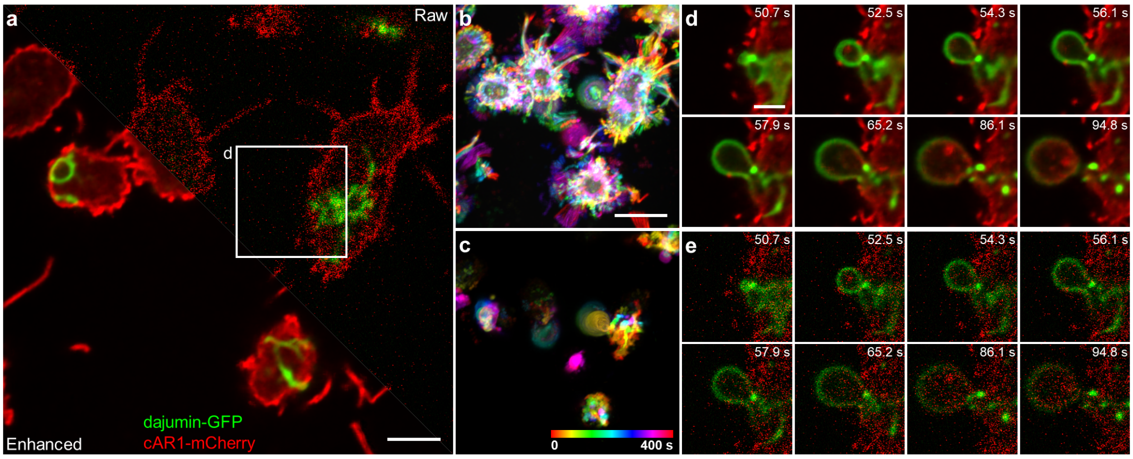

Supplementary Figure 44

DeepSeMi enables high-SNR imaging of contractile vacuole dynamics in photosensitive *Dictyostelium* cells. **a**, DeepSeMi enhanced (left) and raw (right) images of *Dictyostelium* cells, where membranes are labeled in red and contractile vacuoles are labeled in green. 6000 frames are recorded in 400 seconds. Scale bar, 5 μm. **b-c**, Temporal-color coded DeepSeMi enhanced and raw images in **a**, respectively. Scale bar, 10 μm. **d-e**, Recorded time-lapse process of a contractile vacuole generation enclosed by the white box in **a** by DeepSeMi enhanced and raw images, respectively. Scale bar, 3 μm.

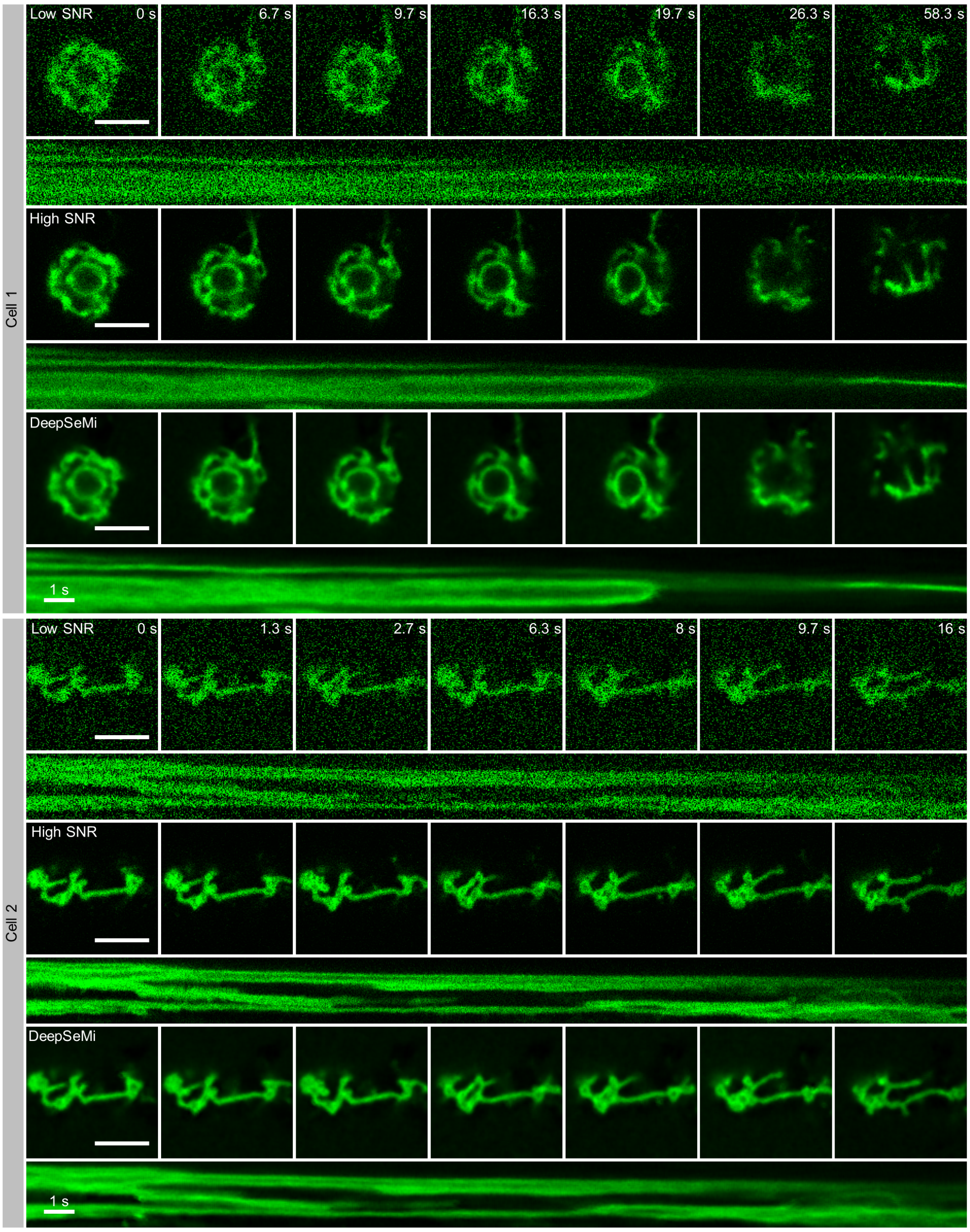

Supplementary Figure 45

Time-lapse imaging of photo-sensitive Dictyostelium cells with simultaneous low- and high-SNR confocal imaging system. Two cells as representatives are presented. For each cell, low-SNR frames, DeepSeMi recovered frames, and high-SNR frames were presented in different rows, respectively. Seven columns represented 7 time points as labeled in the top right corner. Scale bar 2 μm.

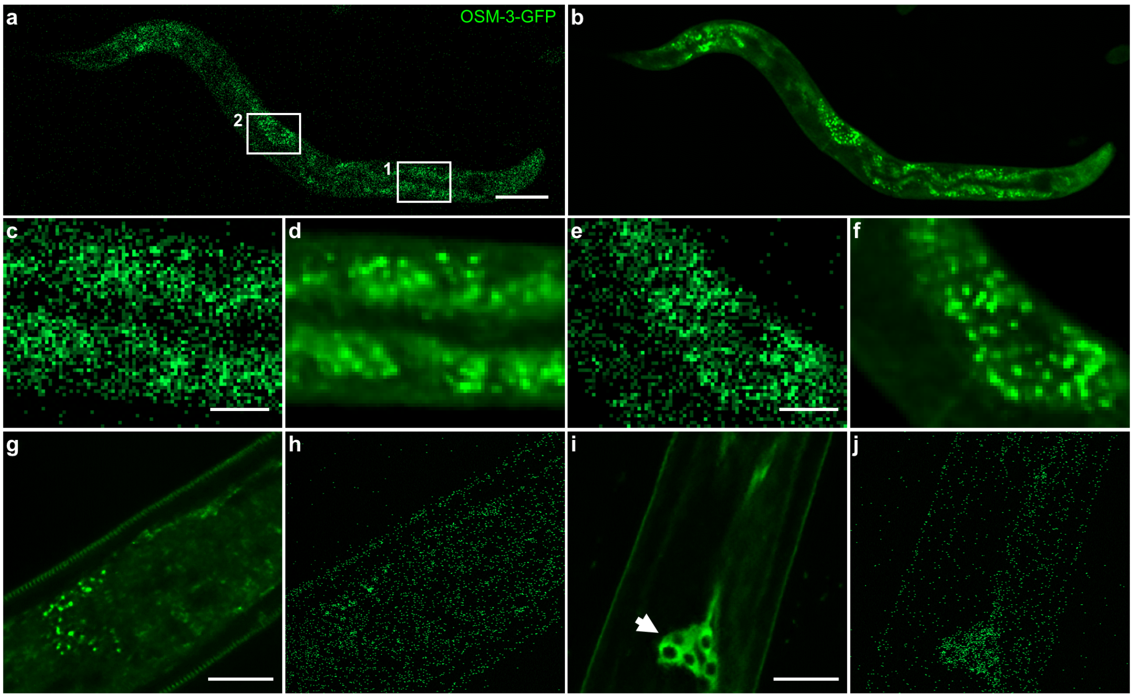

Supplementary Figure 46

DeepSeMi enhanced cellular observation in scattering *C. elegans* in vivo. a-b, *In vivo* imaging of *C. elegans* in a millimeter-scale field-of-view (FOV) with a 10× objective by raw and DeepSeMi-enhanced captures, respectively. Scale bar, 100 µm. **c-d**, Zoom-in panels in the white box ("1") outlined area in **a**, respectively. Scale bar 20 µm. **e-f**, the same as **c-d** but for the white box ("2"). Scale bar 20 µm. **g-h**, *C. elegans* imaging with a 100× objective by DeepSeMi enhanced and raw captures, respectively. Scale bar, 15 µm. **i-j**, Position where the hole shape structure was clearly recovered through DeepSeMi. Scale bar, 15 µm.

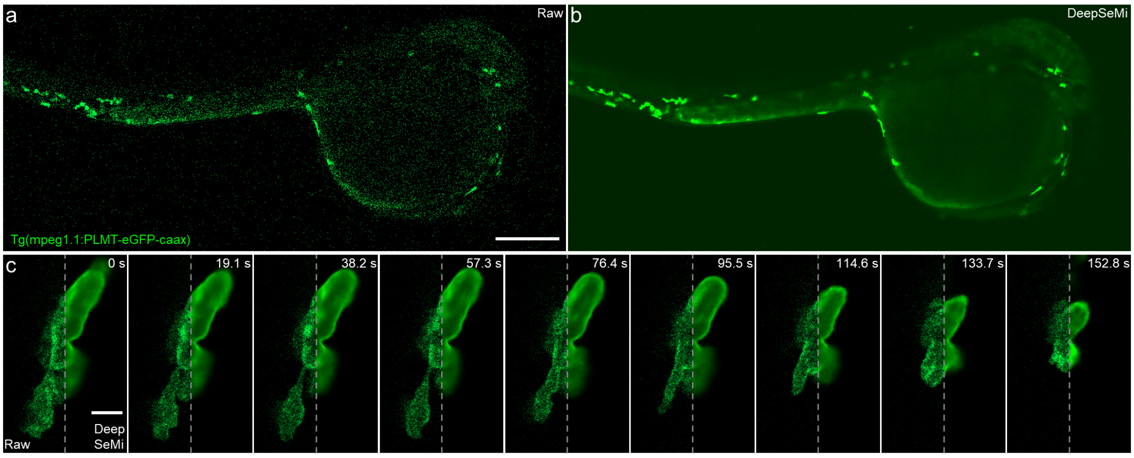

Supplementary Figure 47

DeepSeMi enhances the observation of zebrafish larvae in a low light dosage. **a-b**, Raw and DeepSeMi enhanced observation of zebrafish larvae, respectively. The larva was observed in a commercial confocal microscope with a low magnification objective (10×, NA 0.45). Scale bar, 200μm. **c**, Time-lapse cellular imaging of macrophage in the zebrafish through a high magnification objective (100×, NA 1.45). For each time point, the left part is raw image and the right part is DeepSeMi enhanced image. Scale bar, 5μm.

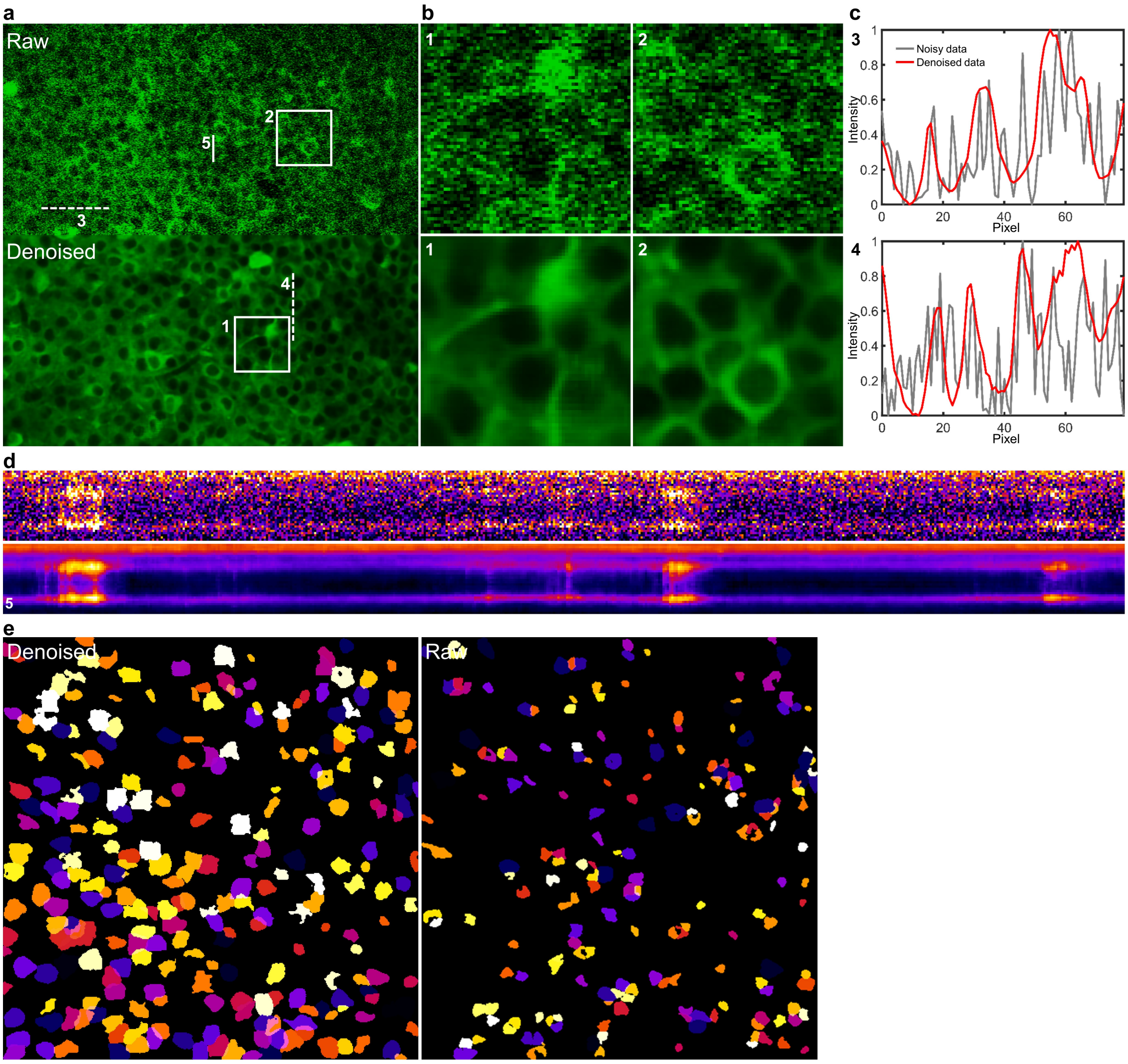

Supplementary Figure 48

DeepSeMi effectively recovers functional data on open-sourced two-photon Neurofinder datasets. **a**, Comparison of denoising results of DeepSeMi (bottom) with raw frame (top) in Neurofinder datasets. The neuronal structures are clearly recovered by DeepSeMi. **b**, Zoom-in panels of the white box outlined regions in a. **c**, Cross-sectional intensity profiles along the white dashed lines in **a**, where red represents the DeepSeMi denoising and the gray represents raw data. **d**, Kymographs (*x*-*t* data) of raw data (top) and DeepSeMi denoised data (bottom) origins from the white solid line in **a**. **e**, Neuron segmentation results of raw (right) and DeepSeMi denoised data (left) through open-source CaImAn package [13]. 196 neurons were extracted from the raw image, and 252 neurons were extracted from the DeepSeMi denoised image.

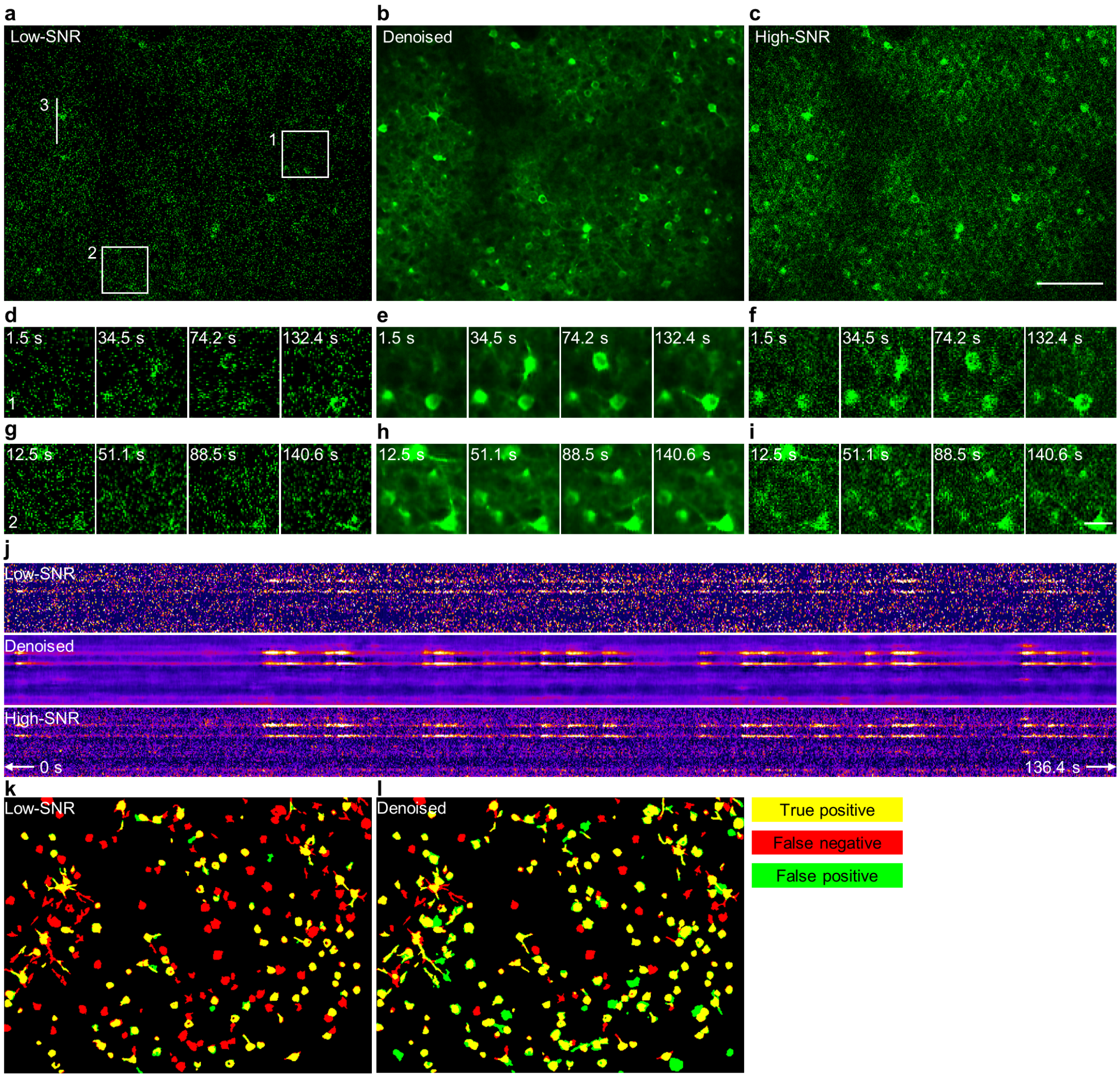

Supplementary Figure 49

Evaluation of DeepSeMi on hybrid high and low-SNR functional imaging. We set up a hybrid two-photon microscope with two channels where one achieves 10-fold SNR compared to the other one. **a-c**, Left to right, low SNR image, DeepSeMi restored image, the high SNR image as a reference. Scale bar, 100 µm. **d-i**, Zoom-in panels of the white box outlined regions in **a-c** at different time points. Scale bar, 20 µm. **j**, Kymographs (x-t data) from the lines in **a-c**. **k-i**, The CNMF segmentation results of low SNR images, restored images and high SNR images. True positives, false positives, and false negatives are annotated.

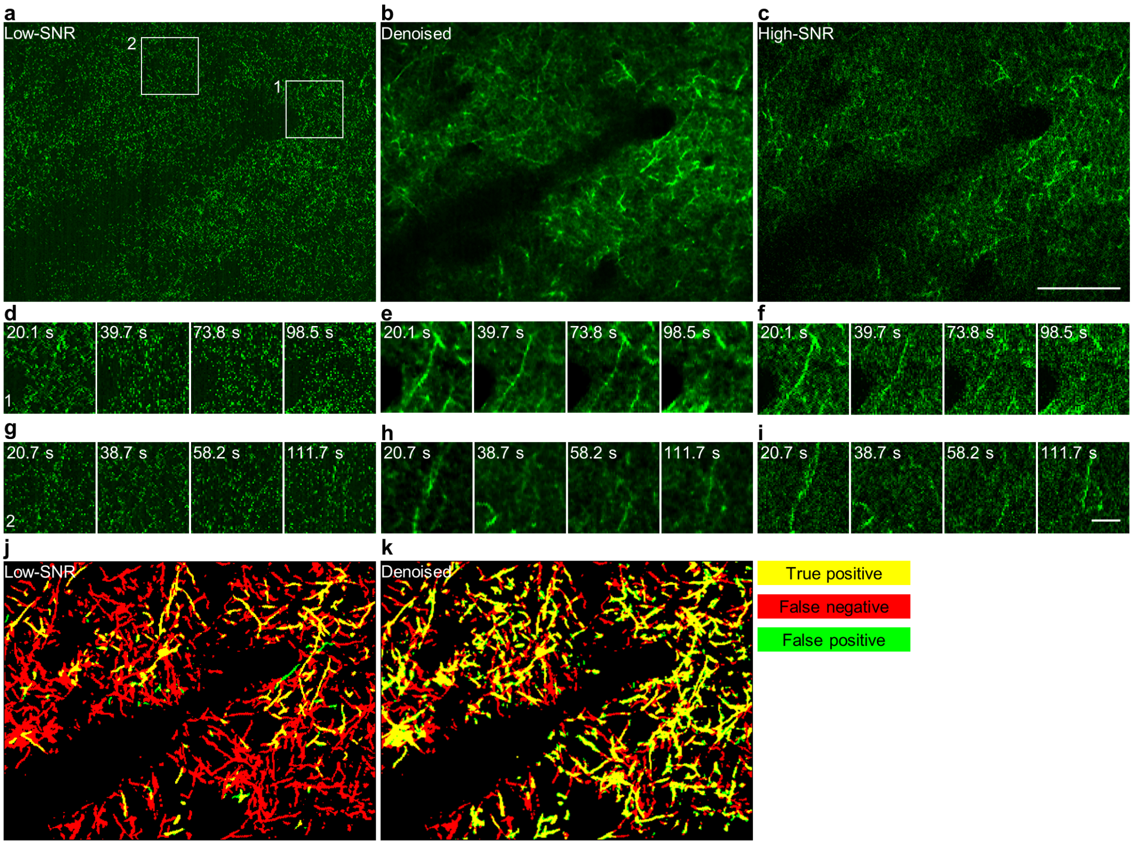

Supplementary Figure 50

Evaluation of DeepSeMi on hybrid high and low-SNR dendritic imaging. **a-c**, Left to right, low SNR image, DeepSeMi restored image, the high SNR image as a reference. Scale bar, 100 μm. **d-i**, Zoom-in panels of the white box outlined regions in **a-c** at different time points. Scale bar, 20 μm. **j-k**, Dendritic segmentation of low-SNR recordigns based on CaImAn. Segmentation based on the high-SNR recording was set as the ground truth. Yellow segments represent true positives, red segments represent false negatives, and green segments represent false negatives. **k** is the same as **j** but by DeepSeMi denoised low-SNR recordings.

References

1. Sheth, D. Y. et al. Unsupervised deep video denoising. In *Proc. IEEE/CVF Int. Conf. Comput. Vis. (ICCV)* (2021).
2. Joshua Batson and Loic Royer. Noise2Self: Blind denoising by self-supervision. In *Proceedings of the 36th International Conference on Machine Learning, volume 97 of Proceedings of Machine Learning Research (PMLR)* (2019).
3. *<Video denoising by sparse 3D transform-domain collaborative filtering.pdf>.*
4. Dabov, K., Foi, A. & Katkovnik, V. Video denoising by sparse 3D transform-domain collaborative filtering. In *Proc. 15th Eur. Signal Process. Conf.* (2007).
5. Yaochen Xie, Zhengyang Wang, and Shuiwang Ji. Noise2Same: Optimizing a self-supervised bound for image denoising. In *Advances in Neural Information Processing Systems*, 20320–30 (2020).
6. Krull, A., Buchholz, T. O. & Jug, F. Noise2void-learning denoising from single noisy images. In *Proceedings of the IEEE/CVF Conference on Computer Vision and Pattern Recognition*, 2129–2137 (2019).
7. Krull, A., Vicar, T., Prakash, M., Lalit, M. & Jug, F. Probabilistic Noise2Void: unsupervised content-aware denoising. *Front. Comput. Sci.* **2**, 5 (2020).
8. Prakash, M., Krull, A. & Jug, F. Fully unsupervised diversity denoising with convolutional variational autoencoders. In *International Conference on Learning Representations* (2021).
9. Dabov, K., et al., Image denoising by sparse 3-D transform-domain collaborative filtering. *IEEE Trans. Image Process.* **16**, 2080–2095 (2007).
10. Li, X., et al. Reinforcing neuron extraction and spike inference in calcium imaging using deep self-supervised denoising. *Nat. Methods* **18**, 1395–1400 (2021)
11. Lecoq, J., et al. Removing independent noise in systems neuroscience data using DeepInterpolation. *Nat. Methods* **18**, 1401–1408 (2021).
12. Sekh, A.A., et al. Physics-based machine learning for subcellular segmentation in living cells. *Nat. Mach. Intell.* **3**, 1071–1080 (2021).
13. Giovannucci, A. et al. CaImAn an open source tool for scalable calcium imaging data analysis. *eLife* **8**, e38173 (2019).
